# Mutation signatures implicate aristolochic acid in bladder cancer development

**DOI:** 10.1186/s13073-015-0161-3

**Published:** 2015-04-28

**Authors:** Song Ling Poon, Mi Ni Huang, Yang Choo, John R McPherson, Willie Yu, Hong Lee Heng, Anna Gan, Swe Swe Myint, Ee Yan Siew, Lian Dee Ler, Lay Guat Ng, Wen-Hui Weng, Cheng-Keng Chuang, John SP Yuen, See-Tong Pang, Patrick Tan, Bin Tean Teh, Steven G Rozen

**Affiliations:** Laboratory of Cancer Epigenome, Division of Medical Sciences, National Cancer Centre Singapore, 11 Hospital Drive, Singapore, 169610 Singapore; Program in Cancer and Stem Cell Biology, Duke-NUS Graduate Medical School, 8 College Road, Singapore, 169857 Singapore; Centre for Computational Biology, Duke-NUS Graduate Medical School, 8 College Road, Singapore, 169857 Singapore; National University of Singapore, Graduate School for Integrative Sciences and Engineering, 28 Medical Drive, Singapore, 117456 Singapore; Department of Urology, Singapore General Hospital, Outram Road, Singapore, 169608 Singapore; Department of Chemical Engineering and Biotechnology, Graduate Institute of Biotechnology, National Taipei University of Technology, 1, Section 3, Chung-hsiao East Road, Taipei, 10608 Taiwan; Division of Urology, Department of Surgery, Chang Gung Memorial Hospital, Linkou, School of Medicine, Chang Gung University, 5, Fusing Street, Gueishan Township, Taoyuan County 333 Taiwan; Division of Cellular and Molecular Research, National Cancer Centre Singapore, 11 Hospital Drive, Singapore, 169610 Singapore; Cancer Science Institute of Singapore, National University of Singapore, Centre for Life Sciences, 28 Medical Drive, Singapore, 117456 Singapore; Genome Institute of Singapore, 60 Biopolis Street Genome, Singapore, 138672 Singapore

## Abstract

**Background:**

Aristolochic acid (AA) is a natural compound found in many plants of the *Aristolochia* genus, and these plants are widely used in traditional medicines for numerous conditions and for weight loss. Previous work has connected AA-mutagenesis to upper-tract urothelial cell carcinomas and hepatocellular carcinomas. We hypothesize that AA may also contribute to bladder cancer.

**Methods:**

Here, we investigated the involvement of AA-mutagenesis in bladder cancer by sequencing bladder tumor genomes from two patients with known exposure to AA. After detecting strong mutational signatures of AA exposure in these tumors, we exome-sequenced and analyzed an additional 11 bladder tumors and analyzed publicly available somatic mutation data from a further 336 bladder tumors.

**Results:**

The somatic mutations in the bladder tumors from the two patients with known AA exposure showed overwhelming AA signatures. We also detected evidence of AA exposure in 1 out of 11 bladder tumors from Singapore and in 3 out of 99 bladder tumors from China. In addition, 1 out of 194 bladder tumors from North America showed a pattern of mutations that might have resulted from exposure to an unknown mutagen with a heretofore undescribed pattern of A > T mutations. Besides the signature of AA exposure, the bladder tumors also showed the CpG > TpG and activated-APOBEC signatures, which have been previously reported in bladder cancer.

**Conclusions:**

This study demonstrates the utility of inferring mutagenic exposures from somatic mutation spectra. Moreover, AA exposure in bladder cancer appears to be more pervasive in the East, where traditional herbal medicine is more widely used. More broadly, our results suggest that AA exposure is more extensive than previously thought both in terms of populations at risk and in terms of types of cancers involved. This appears to be an important public health issue that should be addressed by further investigation and by primary prevention through regulation and education. In addition to opportunities for primary prevention, knowledge of AA exposure would provide opportunities for secondary prevention in the form of intensified screening of patients with known or suspected AA exposure.

**Electronic supplementary material:**

The online version of this article (doi:10.1186/s13073-015-0161-3) contains supplementary material, which is available to authorized users.

## Background

Bladder cancer is a major cause of morbidity and mortality worldwide, causing an estimated 150,000 deaths per year [1]. In developed countries, approximately 90% of bladder cancers are urothelial cancers, that is, cancers of the transitional cells that line the bladder. These cancers can be subclassified as muscle-invasive or non-muscle-invasive. Exposure to environmental carcinogens has long been known to cause bladder cancers. The most prominent of these exposures is inhaled tobacco smoke [2,3]. Additional environmental causes include arsenic [4,5] and certain occupational exposures, such as aromatic amines [6-9]. Chlorinated drinking water is also a possible contributing exposure [10].

An emerging field known as molecular epidemiology augments classical epidemiological approaches with exposure-associated molecular biomarkers to better substantiate causal links. This has significant implications in both clinical oncology and public health. Indeed, inexpensive next-generation sequencing provides a new molecular epidemiological tool for inferring environmental exposures and endogenous mutational processes that occurred prior to and during oncogenesis [11-17]. Somatic mutations can be identified by finding genetic variants in tumors and subtracting from them the variants found in non-malignant tissues from the same individual. Different environmental exposures and endogenous mutagenic processes often have characteristic somatic mutation signatures in terms of single nucleotide changes. These signatures often include preferences for particular bases 5′ and 3′ of the location of the mutation, for example, a preference for G (guanosine) 3′ of C > T mutations, reflecting mutations caused by deamination of 5-methyl cytosine in CpG > TpG contexts. Often tumors show overlays of multiple mutational exposures and processes, which can be deconvolved by approaches based on non-negative matrix factorization (NMF) or software that uses related methods [16,18,19].

Recently, the mutation spectra of bladder tumors from North America and Denmark have been analyzed, and three major signatures were detected [16,17,20-22]. One of these is thought to result from activated APOBEC genes. The second signature corresponds to the aforementioned deamination of 5-methyl cytosines in CpGs. The final mutation signature, Signature 5 [16], is of uncertain origin but partially correlates with tobacco smoking [16].

We and others recently showed that aristolochic acid (AA), which is present in many plants in the genus *Aristolochia* that are used in traditional herbal medicine, induces highly distinctive mutation signatures in the genomes of upper urinary tract urothelial cell carcinomas (UTUC) [14,23-28]. We also found probable AA mutation signatures in hepatocellular carcinomas (HCCs) from southern China, where herbal medicine is widely used, suggesting that AA is a contributory factor in HCC development in this region [14]. Subsequently, AA mutation signatures were detected in renal cell carcinomas (RCCs) from Romania and Croatia [29,30]. The AA signatures were found both in patients from areas where AA exposure was known to be prevalent, but also, importantly, in patients from other areas. These findings in HCCs and RCCs exemplified the utility of mutation signatures for the detection of previously unexpected carcinogenic exposures.

In the study reported here, we investigated whether bladder cancer might also be caused by AA exposure. There is anecdotal evidence that patients with AA-induced kidney failure may have elevated risk of bladder cancer [31-33], and AA-DNA adducts occur in the bladders of AA-fed mice [34,35]. To our knowledge, however, there have been no population-based epidemiological or molecular epidemiological investigations of possible AA exposure in the development of bladder tumors. In the current study, we scrutinized the somatic mutation spectra of 349 bladder cancer exomes to determine if they reveal evidence of AA exposure, starting with tumors from patients with known exposure and then extending the study to tumors from patients with unknown exposure.

## Methods

### Clinical samples and information

Tissue samples and clinical information on two patients with bladder cancer and known AA exposure (130T and 136T) were obtained from the Chang Gung Memorial Hospital in Taiwan. Eleven additional bladder cancers were obtained from Singapore General Hospital in Singapore. Written informed consent was obtained from each subject, the research protocol was approved by the Human Research Ethics Committee of the Chang Gung Memorial Hospital and SingHealth Institutional Review Board, and the research conformed to the Helsinki Declaration. See Table S1 in Additional file 1 for details of the patients treated in Taiwan and Singapore. The somatic mutation data from the 99 bladder cancers treated in China was reported previously [36]. The somatic mutation data from 237 urothelial bladder tumors from The Cancer Genome Atlas (TCGA) [37] were downloaded from TCGA data portal [38] on 8 May 2014. For 130 out of these 237 tumors, somatic data were published previously [39]. Out of the 237 tumors represented in TCGA data, 194 were from patients treated in North America. The treatment locations of the other 43 patients are not clear, as the samples came from biobank organizations. The ethnicities of the 237 TCGA patients were: 'white', 172; 'Asian', 26; 'black or African American', 12; 'unknown', 27. We also analyzed previously reported data on 11 HCCs [40], 24 UTUCs and two AA-exposed cell lines [14]. The total number of tumors analyzed was 386.

### Sequencing and identification of somatic mutations

The genomes of the two AA-associated bladder cancers from Taiwan, the 11 bladder cancers from Singapore, and the respective matched nonmalignant tissue samples were sequenced on an Illumina HiSeq 2000 as paired-end 76-bp reads. Read pairs were aligned to the reference human genome (hg19) using Burrows-Wheeler Aligner [41]. Somatic single nucleotide mutations were identified according to their presence in the tumor genome and absence from the corresponding normal genome using a discovery pipeline based on the Genome Analyzer Toolkit. The sequencing reads for the tumors that were sequenced for this study (Table S2 in Additional file 1) have been deposited at the European Genome-phenome Archive [42] under accession EGAS00001000975.

### Identifying mutation signatures with EMu and non-negative matrix factorization

EMu version 1.5.2 [19] (downloaded from [43]) was used to infer the number of elementary mutational processes and their signatures from the catalogs of somatic mutations in bladder cancer [36,39], UTUC [14], HCC [40], and AA-treated cell line genomes [14]. We ran EMu with default parameters and an 'opportunity file' based on the human exome. We independently confirmed the EMu analysis with the NMF implementation used previously [16] (Figures S8 to 10 in Additional file 1). We ran NMF with the arguments iterationsPerCore = 10, minNumberOfSignature = 2, and maxNumberOfSignature = 8.

### Statistical and computational analysis

Statistical analysis was carried out in R [44]. We used the binom.test function to test for an excess of A:T > T:A mutations and to test for an excess of A > T mutations on the non-transcribed strand (that is, for strand bias). False discovery rates (FDRs) were calculated using the p.adjust function with the fdr method. We used the cosine function in the R package lsa [45] to calculate cosine similarity.

## Results

### Aristolochic acid induces its distinctive mutation signature in bladder cancer

**Figure 1:**
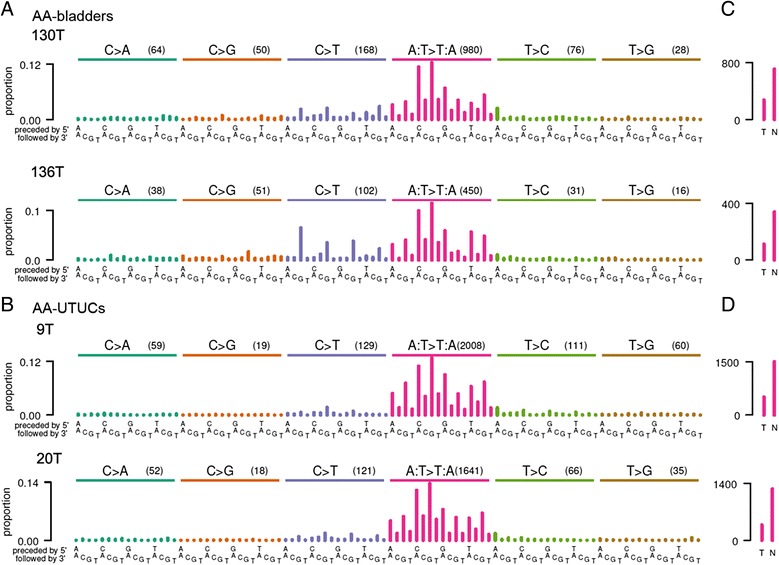
Mutation spectra of AA-bladder cancers and AA-UTUCs. **(A,B)** Somatic mutation proportions in trinucleotide contexts for bladder cancers (130T and 136T) from Taiwan with known AA exposure (A) and AA-UTUCs (9T and 20T) (B). The height of each bar (the *y* axis) represents the proportion of somatic mutations that fall into a particular trinucleotide mutational class, adjusted for the frequency of the trinucleotide in the exome (Table S3 in Additional file 1). Along the *x* axis the mutations are organized first by the nucleotide mutation itself: C > A (turquoise bars), C > G (orange bars), C > T (blue bars), A:T > T:A (red bars), T > C (green bars), T > G (brown bars). For each mutation, the 16 trinucleotide contexts are ordered by the flanking 5′ then 3′ nucleotides. Numbers in parentheses indicate counts of mutations for each single nucleotide substitution (for example, C > A, C > G). Cosine similarities for A:T > T:A mutations were as follows: between 9T and 20T (two UTUCs), 0.989; between 20T and 130T (a UTUC and a bladder cancer), 0.992; between 20T and 136T (a UTUC and a bladder cancer), 0.982. **(C,D)** Strand bias showing counts of A > T mutations (*y* axis) on the transcribed (T) and non-transcribed (N) strands. Many fewer somatic A > T mutations were observed on the transcribed than on the non-transcribed strand in both AA-bladder cancers and AA-UTUCs. *P*-values were computed by one-sided binomial tests compared with the null hypotheses of equal proportions of mutations on the transcribed and non-transcribed strands.

As an initial investigation into whether AA contributes to somatic mutations in bladder cancer, we performed whole-genome sequencing on two tumors from patients with histories of consuming AA-containing herbal remedies. Neither patient had end-stage renal disease (a common consequence of AA-induced nephrotoxicity) nor a history of UTUC or HCC at their first diagnosis of bladder cancer. We focused on the analysis of the exonic portions of these genomes, which harbored 2,054 somatic single nucleotide substitutions - a total of 1,721 different genes were affected by non-silent mutations. These AA-associated bladder cancers had mutation spectra that were similar to those of previously reported AA-associated UTUCs [14] (Figure 1B). The bladder tumors also showed a predominance of A > T transversions that was not seen in previous pan-cancer analyses [17,18] but that is characteristic of AA mutagenesis [16,17]. This predominance of A > T mutations was coupled with a preference for CAG and TAG contexts (that is, CAG > CTG, TAG > TTG; Figure 1A,B), and the overall pattern of A:T > T:A mutations had cosine similarities ranging from 0.977 to 0.992. In addition, both bladder tumors had many fewer A > T mutations on the transcribed compared with the non-transcribed strands: over 70% of the A > T mutations were on the non-transcribed strand (Table 1). Figure 1C shows the number of mutations on the transcribed and non-transcribed strands of the two bladder tumors and the associated *P*-values for strand bias. For comparison, Figure 1D shows that the strand bias and *P*-values for two AA-UTUCs are similar, with > 75% of the A > T mutations on the non-transcribed strand (Table 1).

**Table 1:** **Tumors with high proportions of A:T > T:A mutations**

		**Mutations attributed to AA**	**Mutation count**		**A:T > T:A proportion**		**A > T Count**		**Non-transcribed** **/(all A > T)**	**Strand bias**		**A:T > T:A cosine similarity with AA signature**
**Sample**	**Cancer type**	**Proportion**	**Total**	**A:T > T:A**	***P***	**FDR**	**Non-transcribed**	**Transcribed**		***P***	**FDR**	
9T	UTUC	0.97	2386	2008	0	0	1505	503	0.75	8E-116	3E-113	0.987
20T	UTUC	0.96	1933	1641	0	0	1265	376	0.77	1E-112	2E-110	0.995
K100T	UTUC	0.97	2342	1982	0	0	1435	547	0.72	9E-92	1E-89	0.992
6T	UTUC	0.95	1552	1302	0	0	994	308	0.76	1E-84	9E-83	0.995
K80T	UTUC	0.98	1713	1480	0	0	1100	380	0.74	1E-81	1E-79	0.988
13T	UTUC	0.99	1409	1222	0	0	924	298	0.76	3E-75	2E-73	0.994
K79T	UTUC	0.99	1573	1374	0	0	1015	359	0.74	5E-73	3E-71	0.992
130T	Bladder	0.82	1366	980	0	0	708	272	0.72	1E-45	5E-44	0.992
HK41T	HCC	0.95	781	630	0	0	441	189	0.70	2E-24	7E-23	0.988
3T	UTUC	0.91	666	524	0	0	369	155	0.70	2E-21	6E-20	0.959
136T	Bladder	0.73	688	450	1E-229	4E-228	339	111	0.75	3E-28	1E-26	0.987
10T	UTUC	0.73	545	351	3E-176	9E-175	263	88	0.75	1E-21	4E-20	0.972
HK2B_8d2	AA-treated cell line	0.73	413	264	7E-132	2E-130	178	86	0.67	8E-09	2E-07	0.972
GZ75T	HCC	0.71	404	250	4E-120	1E-118	167	83	0.67	6E-08	2E-06	0.975
HK2_AA	AA-treated cell line	0.66	261	152	2E-68	4E-67	107	45	0.70	3E-07	6E-06	0.905
33324197T	Bladder	0.42	182	67	5E-17	1E-15	46	21	0.69	2E-03	3E-02	0.942
B23	Bladder	0.36	241	76	9E-15	2E-13	56	20	0.74	2E-05	4E-04	0.949
HK174T	HCC	0.48	109	46	1E-14	2E-13	24	22	0.52	0.44	0.85	0.886
HK65T	HCC	0.47	119	48	2E-14	5E-13	41	7	0.85	3E-07	7E-06	0.889
B77	Bladder	0.45	106	42	2E-12	4E-11	38	4	0.90	3E-08	8E-07	0.927
HK81T	HCC	0.45	101	39	3E-11	5E-10	26	13	0.67	3E-02	0.26	0.893
GZ119T	HCC	0.46	59	25	1E-08	2E-07	17	8	0.68	5E-02	0.40	0.736
B89-12	Bladder	0.3	238	62	1E-08	2E-07	48	14	0.77	9E-06	2E-04	0.950
HK267T	HCC	0.43	61	23	5E-07	8E-06	18	5	0.78	5E-03	7E-02	0.833
HK75T	HCC	0.41	67	24	9E-07	1E-05	12	12	0.50	0.58	1	0.871
21T	UTUC	0.34	76	24	1E-05	2E-04	17	7	0.71	3E-02	0.27	0.860
TCGA-K4-A6FZ-01A-11D-A31L-08	Bladder	0.18	461	83	4E-04	6E-03	52	31	0.63	1E-02	0.15	0.525
1T	UTUC	0.27	70	18	2E-03	3E-02	17	1	0.94	7E-05	1E-03	0.777

HCC, hepatocellular carcinoma; NA, not applicable; UTUC, upper urinary tract urothelial cell carcinoma.

### Aristolochic acid exposure in bladder cancers from other populations

**Figure 2:**
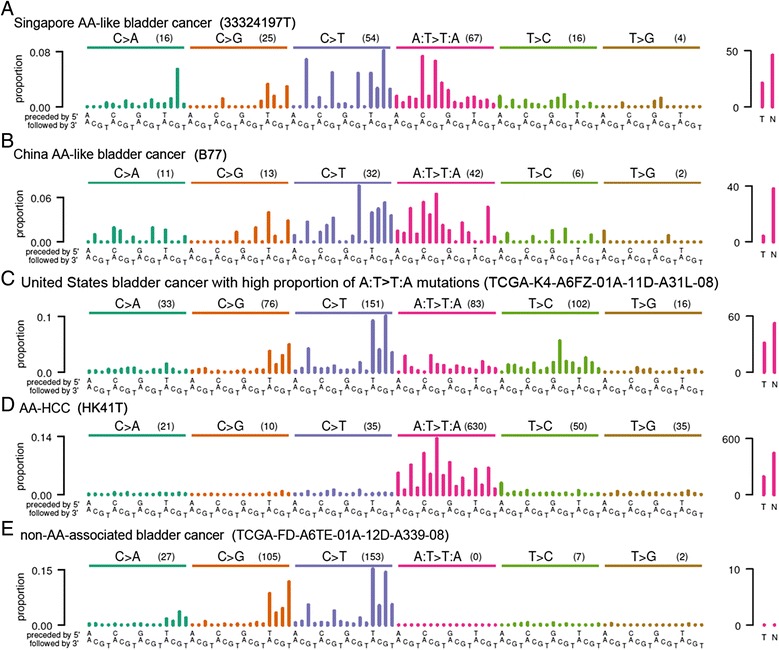
Mutation spectra and strand bias of examples of cancers with and without evidence for AA exposure. **(A,B)** Bladder cancers (33324197T, B77). **(C)** Bladder cancer with statistically significant over-representation of A:T > T:A mutations and strand bias but with a pattern of trinucleotide contexts for A:T > T:A mutations notably dissimilar from tumors with known AA exposure (TCGA-K4-A6FZ-01A-11D-A31L-08). **(D)** HCC (HK41T). **(E)** For comparison, a bladder cancer (TCGA-FD-A6TE-01A-12D-A339-08) without evidence of AA exposure. Plotting conventions are as for Figure 1. In total, 6 of 349 bladder cancers showed evidence of AA exposure (Figure S1 in Additional file 1).

Having established that AA can contribute to somatic mutations in bladder cancer, we next examined the somatic mutation catalogs from a larger set of 347 bladder cancers for evidence of AA-induced mutations. This set consisted of 11 tumors from Singapore that we sequenced for this study, as well as 336 publicly available whole-exome or whole-genome sequenced tumors. Of these, 99 were non-muscle-invasive tumors from patients treated in China [36], and the remaining 237 were muscle-invasive tumors from patients treated either in North America (194 patients) or at unknown locations (43 patients) [39]. Examination of the somatic mutation spectra of these tumors revealed some with the characteristics of AA exposure in the form of elevated proportions of A > T mutations with a marked bias against mutations on the transcribed strand (Figure 2A-E; Figures S2 to S7 in Additional file 1).

**Figure 3:**
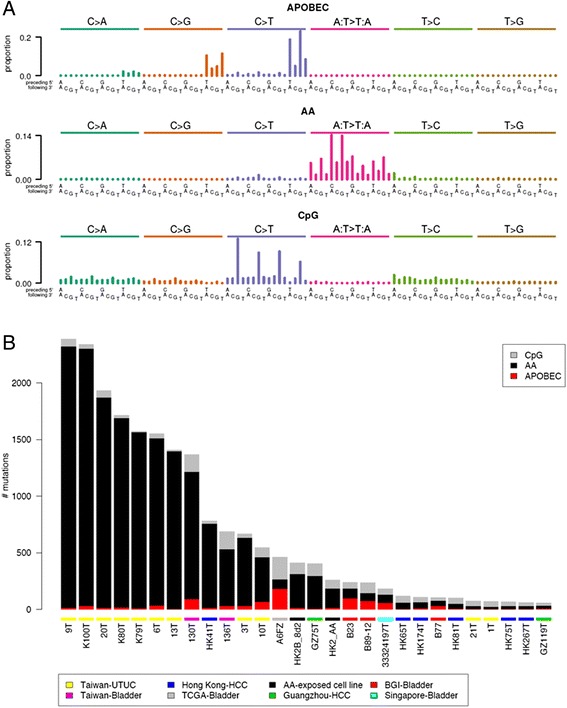
Inferred mutation signatures and their contributions to somatic mutations in bladder cancers, UTUCs, and HCCs. **(A)** EMu discerned three mutation signatures. The height of each bar (the *y* axis) represents the proportion of mutations in the inferred signature that fall into a particular trinucleotide mutation class, adjusted for the frequency of the trinucleotide in the exome. **(B)** The contributions of each mutation signature (that is, inferred mutational process) to the somatic mutations in each tumor in Table 1. BGI-Bladder: tumors reported in reference 36.

To systematically explore this observation, we used EMu [19] to extract mutation signatures from the exomes of all 349 cases (including the two from Taiwan), plus exomes from previously published AA-exposed and non-exposed UTUCs, HCCs, and cell lines (a total of 37 additional exomes). EMu distinguished three mutation signatures in these tumors (Figure 3A). Each signature was characterized by a different pattern of the 96 potential mutations in a trinucleotide context, and each signature contributed to a different proportion of the mutations in each of the tumors (Figure 3B). Notably, EMu discerned a signature almost identical to the mutation spectra observed in UTUCs strongly mutagenized by AA, as can be seen by comparing the EMu AA signature in Figure 3A with the spectra in Figure 1B (cosine similarities for A:T > T:A mutations >0.987). To our knowledge, this signature has not previously been reported in bladder cancers [16,17,20]. In addition to the AA signature, EMu discerned the previously reported signatures of CpG > TpG mutations and of activated APOBECs (C > T and C > G mutations in TCW trinucleotide contexts, where W denotes A or T; Figure 3A) [16,17,20]. To further confirm the EMu analysis, we also analyzed the same set of tumors with NMF [16,17] and found nearly identical signatures (cosine similarity 0.9988 between the EMu and NMF AA signatures) and similar estimates of the AA signature’s contributions for these tumors (Figures S8 to S10 in Additional file 1).

As part of their analyses, EMu and NMF estimate the contribution of each mutation signature to the somatic mutations of each tumor (Table 1). However, neither EMu nor NMF provides a statistical hypothesis test of the presence or absence of a particular signature in a given tumor. Indeed, both EMu and NMF often attribute at least a small number of mutations to every available signature, which for many exposures does not correspond to a biologically plausible level of exposure. Therefore, we developed statistical tests for likely AA exposure based on two characteristics of AA mutagenesis. One was the high proportion of A:T > T:A mutations (Figure 3A), which are rare among other known mutation signatures [16,17]. The other characteristic was the bias against the presence of A > T mutations on the transcribed (antisense) strand, which is presumed to result from transcription-coupled repair of AA-derived adenine adducts on this strand. To statistically test for AA exposure, we made use of the high proportion of A:T > T:A mutations caused by AA and the fact that, among previously reported signatures in bladder cancer, Signature 5 has the highest proportion of A:T > T:A mutations (Figure S7 in Additional file 1; Figure S31 in reference [16]). Specifically, we adopted the null hypothesis that the A:T > T:A mutations in a given tumor were derived only from Signature 5 and calculated as a *P*-value the probability that Signature 5 alone, with no contribution from AA mutagenesis, was responsible for the observed proportion of A:T > T:A mutations. The proportion of A:T > T:A mutations in Signature 5 is 12.5% [16]. This null hypothesis was conservative in that the other signatures previously observed in bladder tumors have far lower proportions of A:T > T:A mutations than Signature 5. We calculated FDRs across the *P*-values of all tumors, adopting a threshold of 0.05 as indicating possible AA exposure. Seven bladder cancers (including the two from patients in Taiwan) met this threshold, with the highest FDR being 0.006 (Table 1). In addition to the two tumors from Taiwan, the bladder cancers with possible AA signatures comprised one out of the 11 tumors treated in Singapore (Figure 2A; Figures S1 and S2 in Additional file 1), three of the 99 tumors treated in China (Figure 2B, Additional file 1: Figures S1, S3) and one of the 194 tumors treated in North America (Figure 2C; Figures S1 and S4 in Additional file 1; Table 1). The mutation spectra of these seven tumors, together with AA-exposed UTUCs and HCCs for comparison, are shown in Figures 1A and 2A-C and Figures S1, S5 and S6 in Additional file 1. These tumors included three muscle-invasive tumors (two from Taiwan and one from the United States) and four non-muscle-invasive tumors (one from Singapore and three from China).

We then confirmed this analysis using the second characteristic of AA mutagenesis: bias against A > T mutations on the transcribed strand. We calculated *P*-values based on the observed proportions of A > T mutations on the transcribed and non-transcribed strands and the null hypothesis of an equal number of A > T mutations on both strands. All seven bladder tumors had raw *P*-values <0.0004 and FDRs <0.017 (one-sided binomial tests; Table 1).

All but one of the bladder cancers that were identified as due to, or as possibly due to, AA exposure, based on the criteria above, had patterns of trinucleotide contexts for A:T > T:A mutations that were very similar to the EMu-extracted AA signature (Figure 3A). This was indicated by A:T > T:A cosine similarities >0.92 for all the bladder cancers in Table 1 except one. The exception was a tumor of a white, 86-year-old patient who was treated in the United States (TCGA-K4-A6FZ-01A-11D-A31L-08). This tumor showed relatively higher rates of 5′-TC-3′ to 5′-AC-3′ (equivalently, 5′-GA-3′ to 5′-GT-3′) mutations (Figure 1C). This was a pattern not seen in the EMu-extracted AA signature (Figure 3A) or in tumors with predominant AA mutations (Figure 1). This dissimilarity was reflected by a cosine similarity of only 0.53 between this tumor and the EMu-extracted A:T > T:A AA signature. Clinical history that would indicate whether this patient used herbal remedies was unavailable. However, this patient was a lifelong non-smoker, suggesting that tobacco smoke would not have been a source of the A > T mutations in this tumor. Thus, the statistically significant over-representation of A:T > T:A mutations and the strand bias in this tumor may be due not to AA but to another, unknown mutagen.

## Discussion

We have presented evidence of AA mutagenesis in the genomes of bladder cancers in patients known to have been exposed to AA, as well as evidence of AA exposure in a subset of bladder cancers from three patient populations. First, we found evidence of AA exposure in 3 of 99 bladder cancers treated in China. This is consistent with the fact that plants containing AA are components of traditional Chinese medicine [46]. Indeed, AA-containing herbal remedies are still readily available in China [47]. We also observed one likely AA-exposed case of bladder cancer from Singapore where traditional Chinese medicine is also widely used. Because the patient was 80 years old, he may have been exposed to AA before its use was prohibited in Singapore. Although HCCs were not the focus of this study, consistent with our previous report [14], the analysis here showed that many HCCs have A:T > T:A mutation patterns very similar to that of the AA signature (Figure 3A). HK41T had a cosine similarity with the EMu-extracted A:T > T:A signature of 0.99 (Figure 1D, Table 1) and GZ75T had a similarity of 0.975 (Table 1).

Our data demonstrating a molecular link between AA and bladder cancer are consistent with previous work showing the presence of AA adducts in the bladders of AA-exposed mice [34,35], with the role of AA in UTUCs [14,23,24], and with previously reported elevated risk of bladder cancer in patients with AA-induced kidney failure [31-33]. However, to our knowledge, the present research is the first to connect AA mutagenesis with bladder cancer in patients from the general population without a history of AA-induced kidney failure.

Although one bladder tumor from Singapore and three bladder tumors from China showed patterns of A:T > T:A mutations that were very similar to those in tumors with known AA exposure (Table 1; Figure S1 in Additional file 1), it remains a formal possibility that these mutations arose from exposure to an unknown mutagen with effects very similar to those of AA. At present, no such mutagen is known, but the mutation signatures of most mutagens remain unstudied. Nevertheless, the reasonable prior possibility of AA exposure through herbal remedies in the tumors from Singapore and China would argue that the mutagen in these tumors was in fact AA.

Associations between exposures and disease are often difficult to establish based on epidemiological data alone [48,49]. Various 'omics' approaches, such as transcriptomics, metabolomics, proteomics, and genomics, are now being evaluated as tools for assessing environmental exposures and cancer risk [50]. Here, we provide an example of using recent advances in sequencing technologies to detect a new likely link between AA mutagenesis and bladder cancers, in addition to its links with UTUC, HCC, and RCC. This was possible because the AA signature has been established in cell culture [14,25] and because previous epidemiological data have linked AA to UTUCs [24,28,51].

The medicinal use of AA-containing plants has a long and geographically diverse history and likely remains widespread despite efforts at regulation in some countries [51-54]. This is probably especially true in highly populous countries in Asia: China and India alone have a combined population of >2.6 billion, and although use of AA-containing plants in Chinese herbal medicine has received much attention, AA-containing plants are probably also widely used in the Indian subcontinent [53,54]. In addition, there is evidence that AA-containing plants are also used in Central and South America [53]. Although use of these plants does not appear to be widespread in the US, their sale or use as botanical products without claims of health benefits are not restricted there. Under the Dietary Supplement Health and Education Act of 1994, the US Food and Drug Administration has little ability to regulate botanical products, although it has urged manufacturers and distributors to ensure that botanical products are free of AA and advised consumers to not use products that might contain AA [55-57].

## Conclusions

Large populations are using traditional herbal medicines that prescribe AA-containing plants, and there is new, rapidly emerging molecular epidemiological evidence of AA exposure in several types of cancer, including bladder cancer, not previously linked to AA. There is also additional exposure to AA due to unintended contamination of food with AA-containing plants. Thus, multiple lines of evidence point to AA exposure as a worldwide public health issue that should be addressed by further investigation and by primary prevention through regulation and public education. In addition to opportunities for primary prevention, knowledge of AA exposure would provide opportunities for secondary prevention in the form of intensified screening of patients with known or suspected AA exposure.

## Additional file

Additional file 1: **Supplementary material.** Contains Supplementary Materials and Methods, Supplementary Figures S1 through S10, Supplementary Tables S1 through S3, and Supplementary References.

## Abbreviations

AA: aristolochic acid
FDR: false discovery rate
HCC: hepatocellular carcinoma
NMF: non-negative matrix factorization
RCC: renal cell carcinoma
TCGA: The Cancer Genome Atlas
UTUC: upper urinary tract urothelial cell carcinoma
