# Supplementary material for: Mutation signatures implicate aristolochic acid in bladder cancer development
Source: Genome Med. 2015 Apr 28;7(1):38. doi: 10.1186/s13073-015-0161-3 (PMC4443665; doi:10.1186/s13073-015-0161-3)
Supplement: Supplementary file 1 — Supplementary material. Contains Supplementary Materials and Methods, Supplementary Figures S1 through S10, Supplementary Tables S1 through S3, and Supplementary References. [file 13073_2015_161_MOESM1_ESM.pdf]

Supplementary Materials for

Mutation Signatures Implicate Aristolochic Acid in Bladder Cancer Development

Song Ling Poon, Mi Ni Huang, Choo Yang, John R. McPherson, Willie Yu, Hong Lee Heng, Anna Gan, Swe Swe Myint, Ee Yan Siew, Lian Dee Ler, Lay Guatt Ng, Wen-Hui Weng, Cheng-Keng Chuang, John Yuen, See-Tong Pang, Patrick Tan, Bin Tean Teh and Steven G. Rozen

Table of Contents

Supplementary Materials and Methods 2

Supplementary Figure S1. The mutation spectra of 28 tumors with high proportions of A:T > T:A mutations and of two AA-treated cell lines. 3

Supplementary Figure S2. The mutation spectra of 11 bladder cancers from patients treated in Singapore. 7

Supplementary Figure S3. The mutation spectra of 99 bladder cancers from patients treated in China. 9

Supplementary Figure S4. The mutation spectra of 237 bladder cancers with data from TCGA [2]. 22

Supplementary Figure S5. The mutation spectra of 24 AA-associated and non-AA associated UTUCs [3]. 52

Supplementary Figure S6. The mutation spectra of 11 AA associated and non-AA associated HCCs [4]. 55

Supplementary Figure S7. The mutation spectra of two AA-treated cell lines [3] 57

Supplementary Figure S8. Mutation signatures detected by NMF were substantially similar to those detected by EMu. 58

Supplementary Figure S9. The correlation of AA counts between EMu and NMF analyses. 59

Supplementary Figure S10. The correlation of AA proportion between EMu and NMF analyses. 60

Supplementary Table S1. Clinical characteristics of 13 bladder cancer patients analyzed by whole-genome or whole exome sequencing.. 61

Supplementary Table S2. Sequence analysis summary of 13 bladder tumors and matched normal tissue 61

Supplementary Table S3. Frequencies of trinucleotides centered at A in the human exome.. 63

Supplementary References 64

Supplementary Materials and Methods

Whole genome and exome sequencing

Illumina TruSeq DNA Sample Prep Kit (Illumina Inc.) was used for preparation of DNA for whole genome shotgun (WGS) libraries while SureSelect Human All Exon kit v3 was used to enrich the coding sequence of 11 bladder cancers the adjacent normal tissues as previously described [1]. Whole genome and whole exome libraries were sequenced on an Illumina HiSeq 2000 as paired-end 76-base pair reads.

Bioinformatic analysis of genome and exome

We used our routine pipeline for the variant calling for both genome and exome analysis [1]. We used the Burrows-Wheeler Aligner (BWA, http://bio-bwa.sourceforge.net/) to align the sequence reads to the human reference genome NCBI GRC Build 37 (hg19) and employed SAMtools (http://samtools.sourceforge.net/) to remove PCR duplicates. Single-nucleotide variants (SNVs) were detected using the Genome Analyzer Toolkit (GATK) pipeline. Only reads with mapping quality ≥ 30 and ≤ 3 mismatches within a 40-bp window were used as input for the genotyper. Additional quality filters (quality by depth ≥ 3, variant depth ≥ 10 and normal depth ≥ 5) were used and any SNV that are close to a micro-indel or several other SNVs will be discarded. dbSNPv135 (http://www.ncbi.nlm.nih.gov/projects/SNP/) and 1000 Genomes Project databases (http://www.1000genomes.org/) were used to discard any common SNPs. W used Sanger capillary sequencing to validate selected mutations.

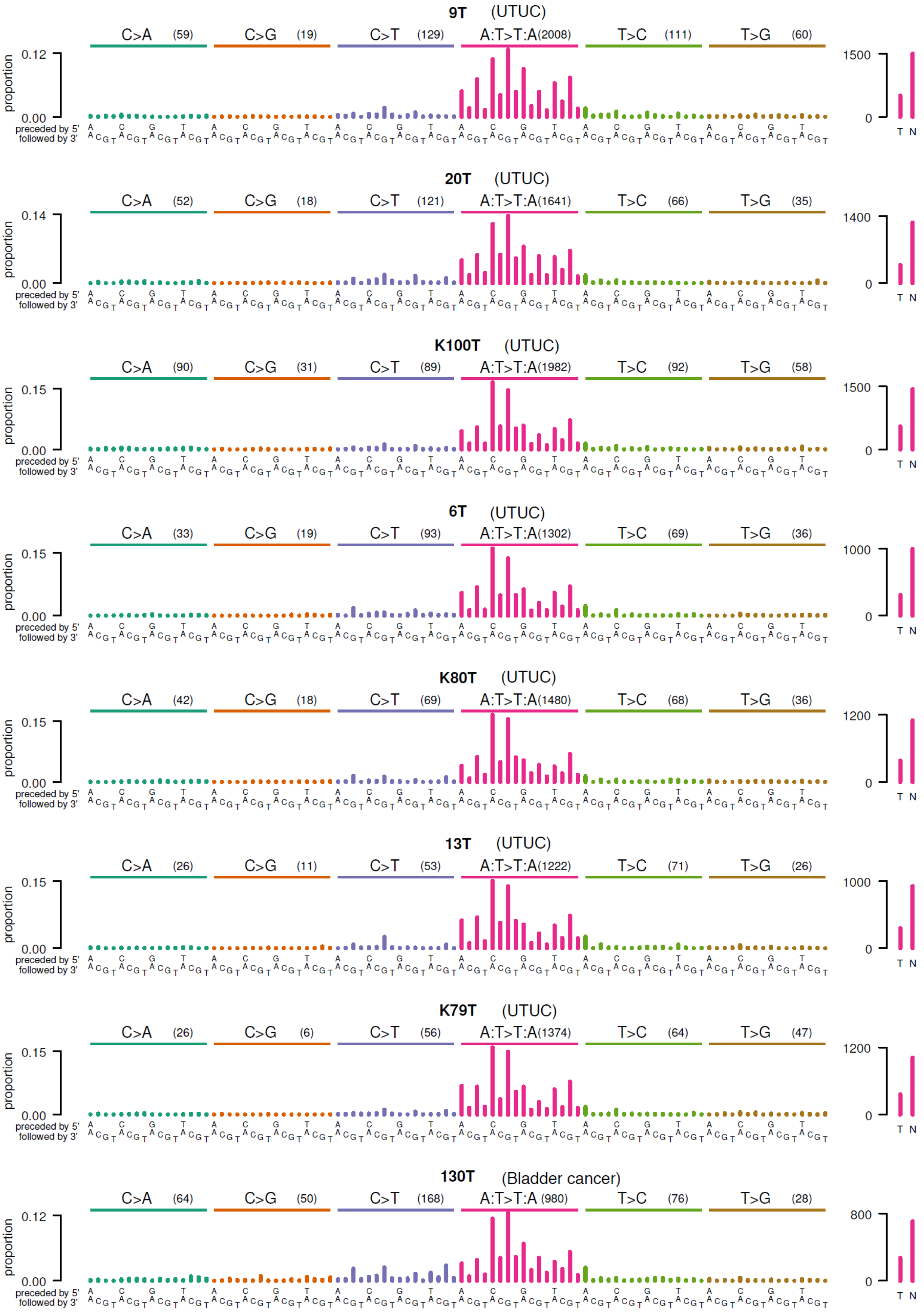

Supplementary Figure S1. The mutation spectra of 28 tumors with high proportions of A:T > T:A mutations and of two AA-treated cell lines.
The tumors included UTUCs, HCCs, and bladder cancers.

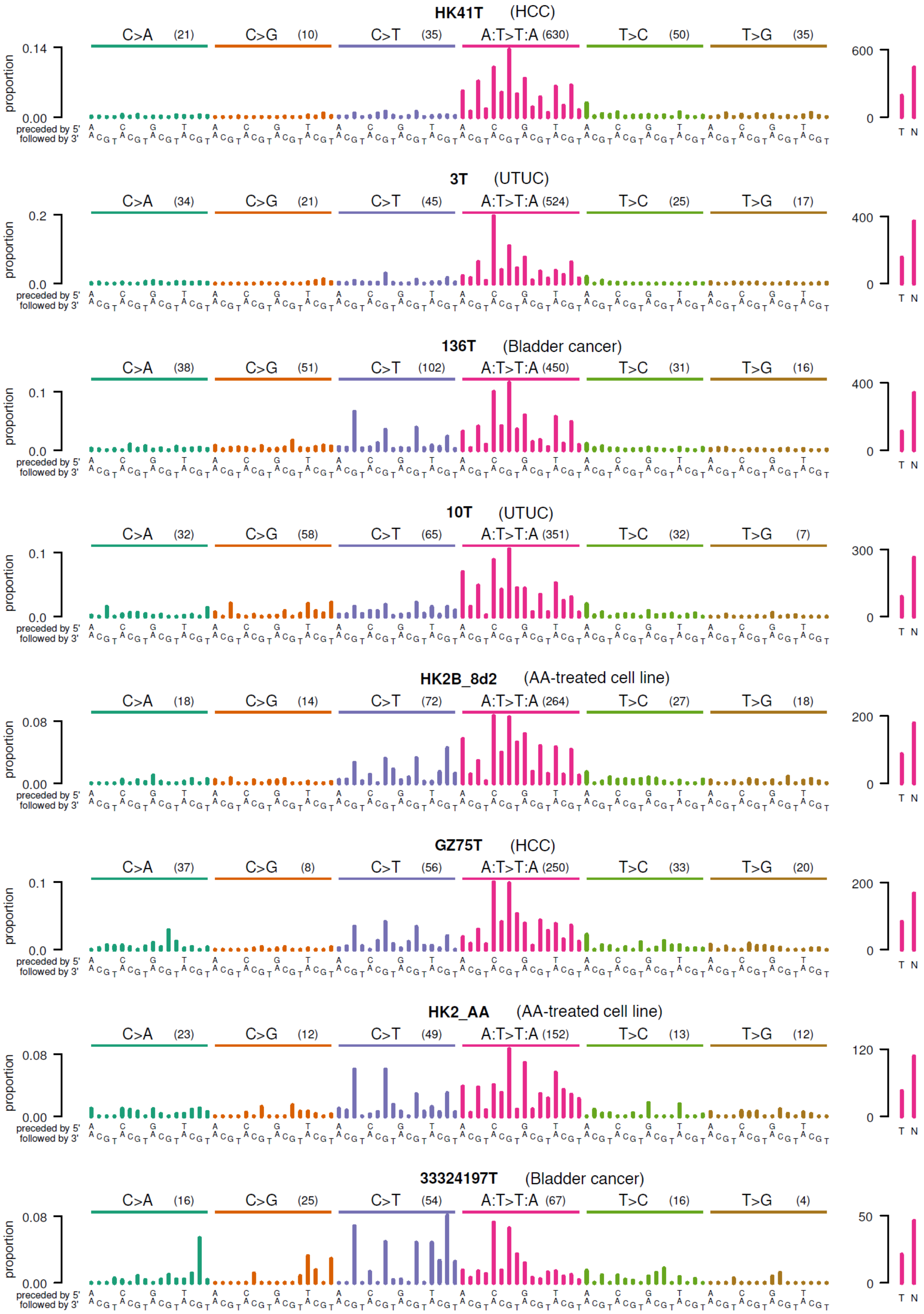

Supplementary Figure S1 continued. The mutation spectra of 28 tumors with high proportions of A:T > T:A mutations and of two AA-treated cell lines.
The tumors included UTUCs, HCCs, and bladder cancers.

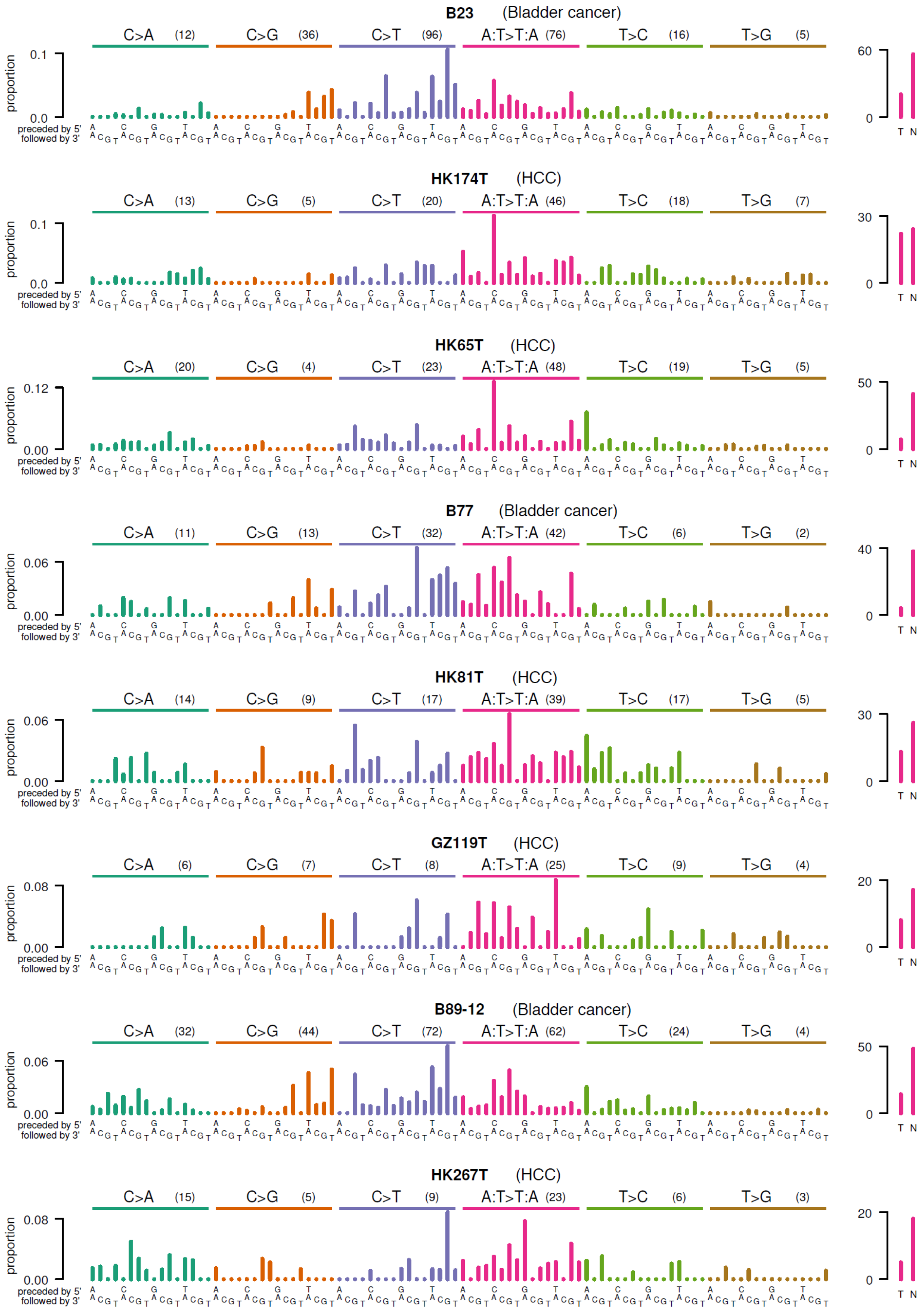

Supplementary Figure S1 continued. The mutation spectra of 28 tumors with high proportions of A:T > T:A mutations and of two AA-treated cell lines.
The tumors included UTUCs, HCCs, and bladder cancers.

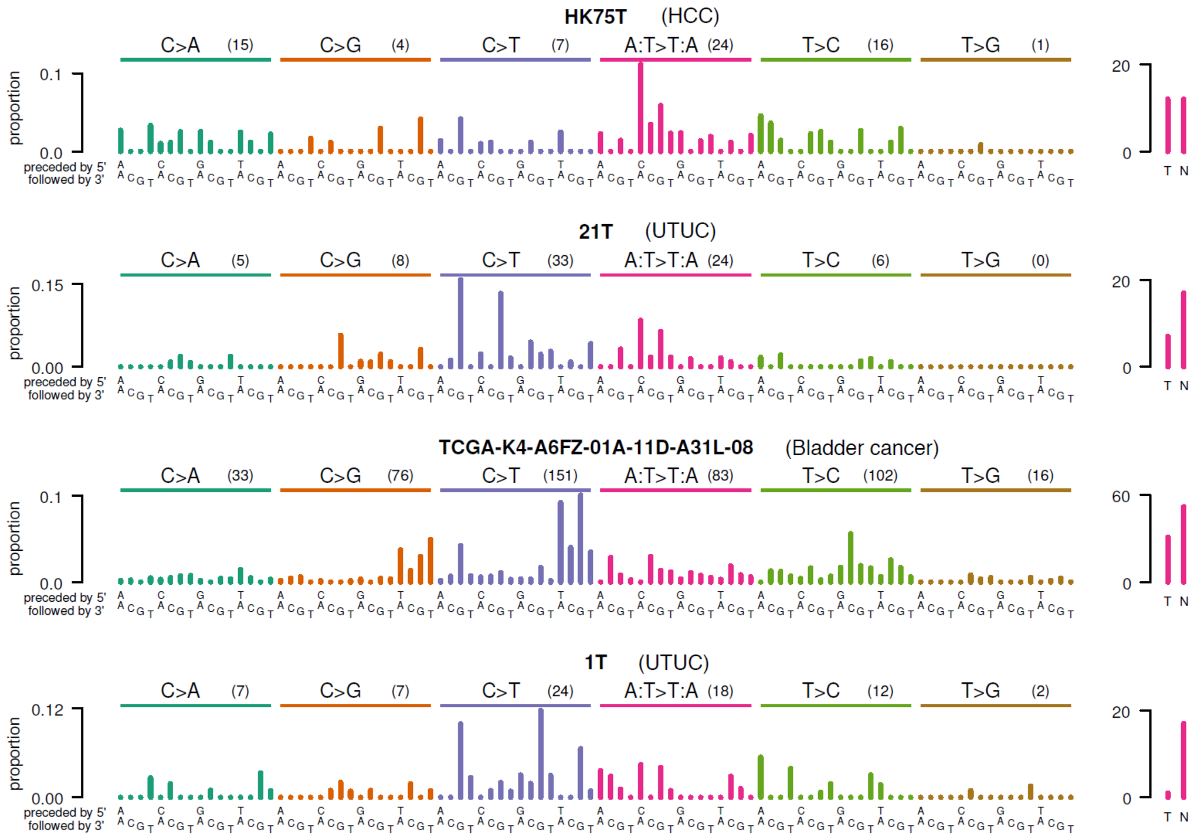

Supplementary Figure S1 continued. The mutation spectra of 28 tumors with high proportions of A:T > T:A mutations and of two AA-treated cell lines.The tumors included UTUCs, HCCs, and bladder cancers.

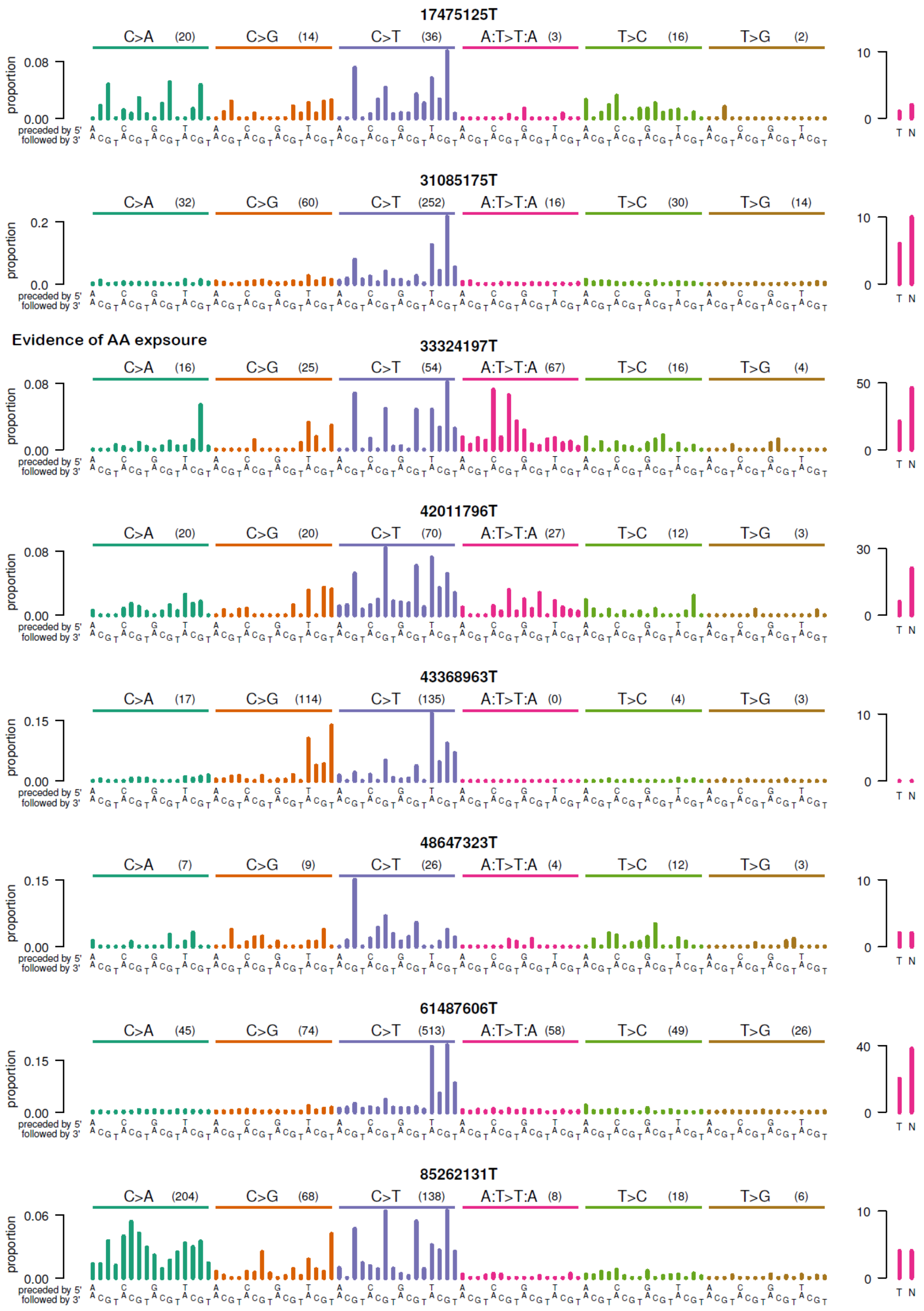

Supplementary Figure S2. The mutation spectra of 11 bladder cancers from patients treated in Singapore.

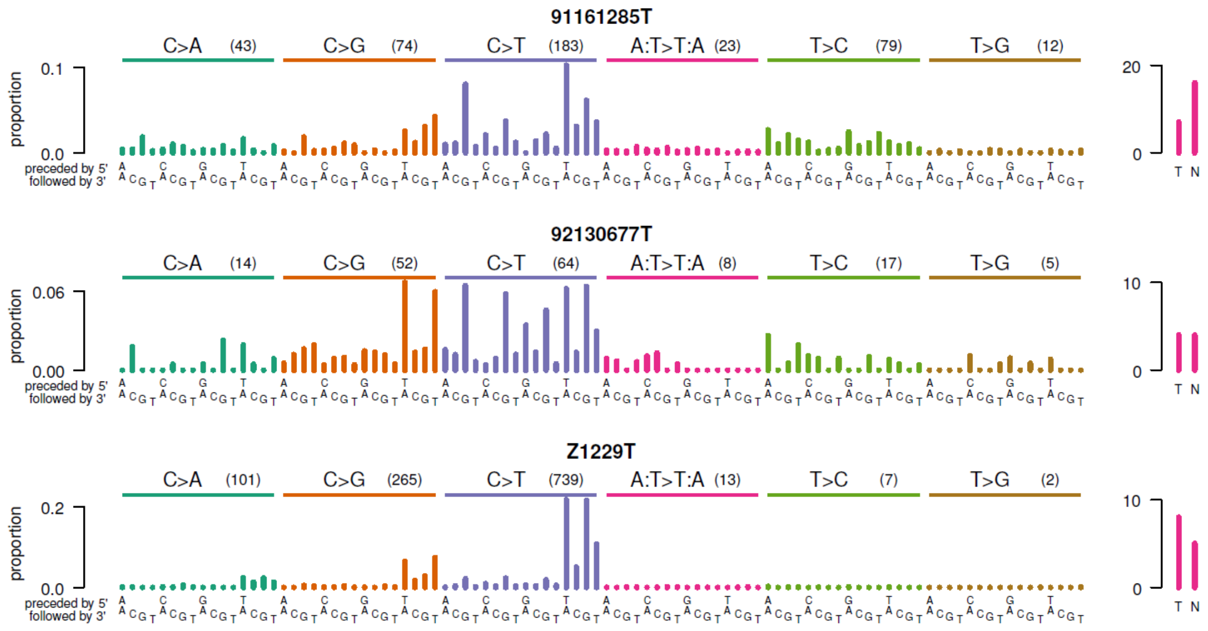

Supplementary Figure S2 continued. The mutation spectra of 11 bladder cancers from patients treated in Singapore.

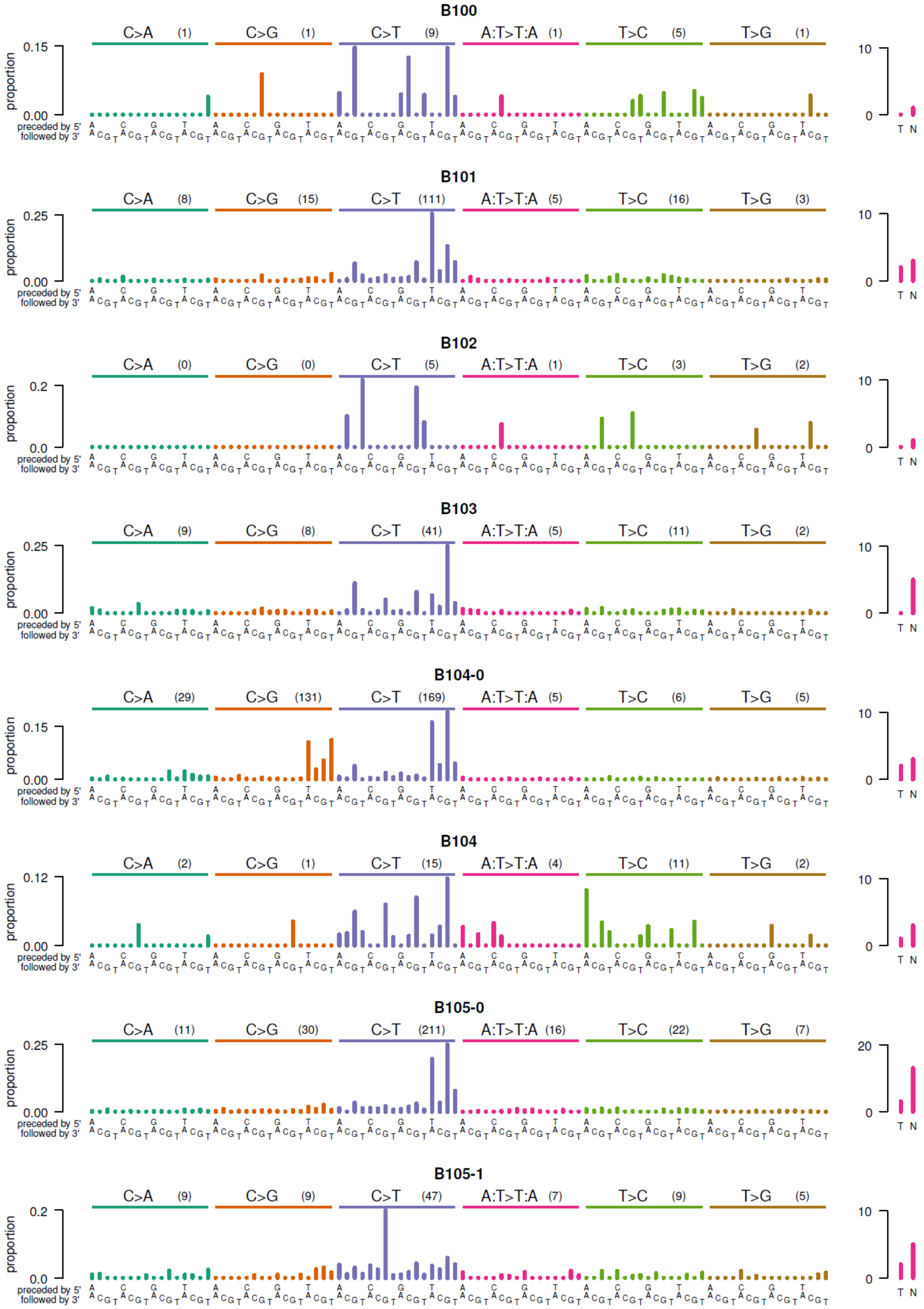

Supplementary Figure S3. The mutation spectra of 99 bladder cancers from patients treated in China.

Data from Guo et al [2].

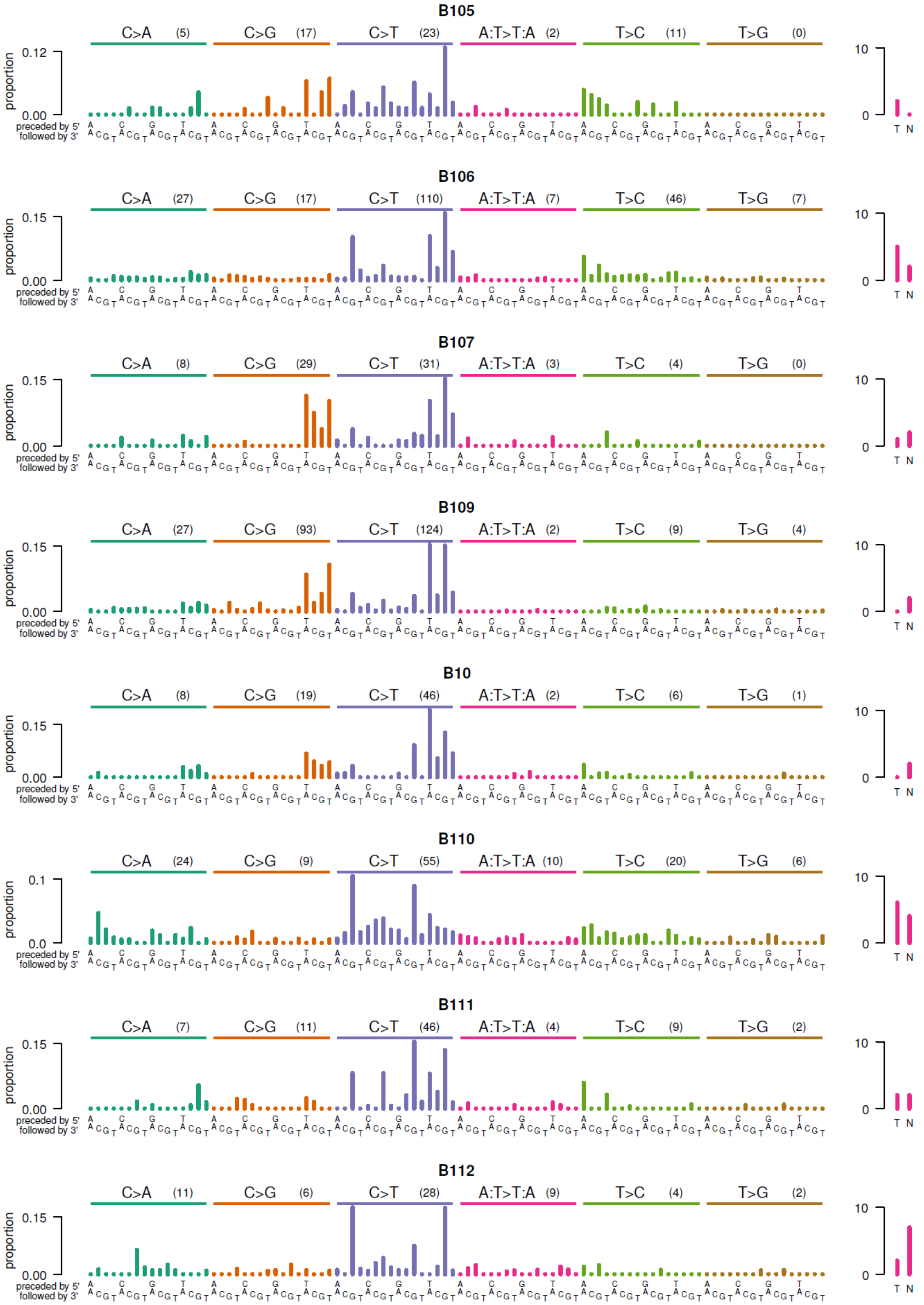

Supplementary Figure S3 continued. The mutation spectra of 99 bladder cancers from patients treated in China.

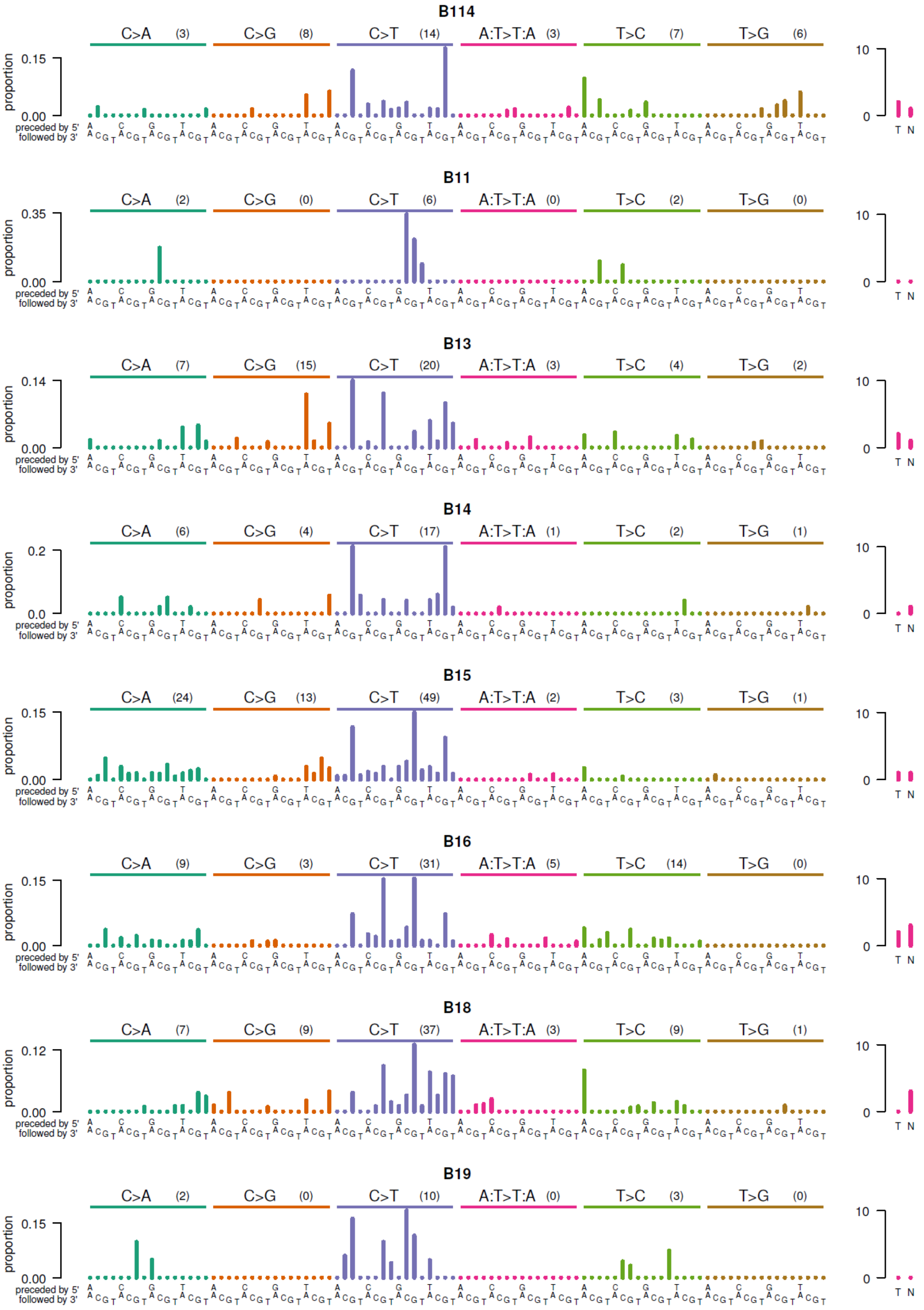

Supplementary Figure S3 continued. The mutation spectra of 99 bladder cancers from patients treated in China.

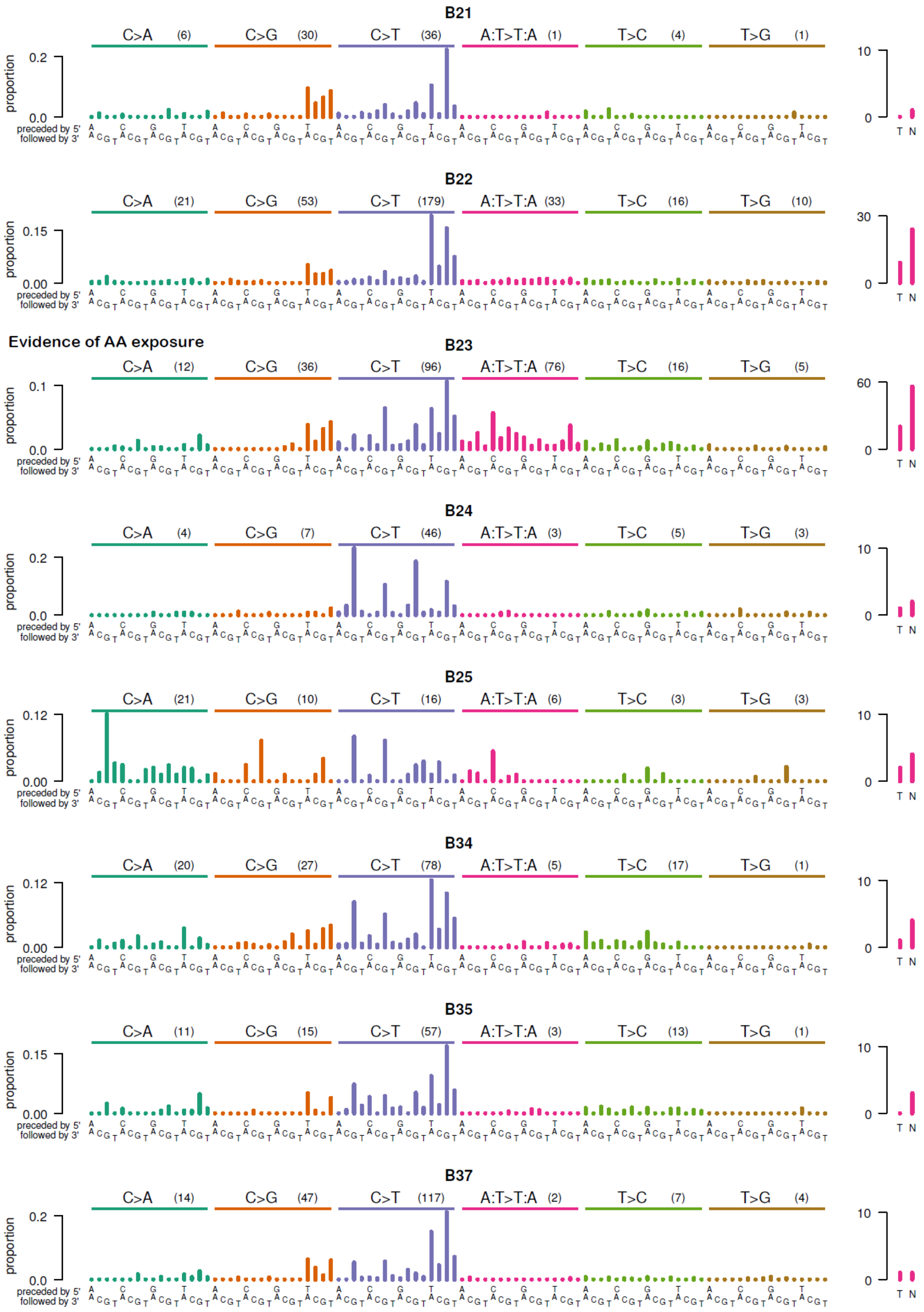

Supplementary Figure S3 continued. The mutation spectra of 99 bladder cancers from patients treated in China.

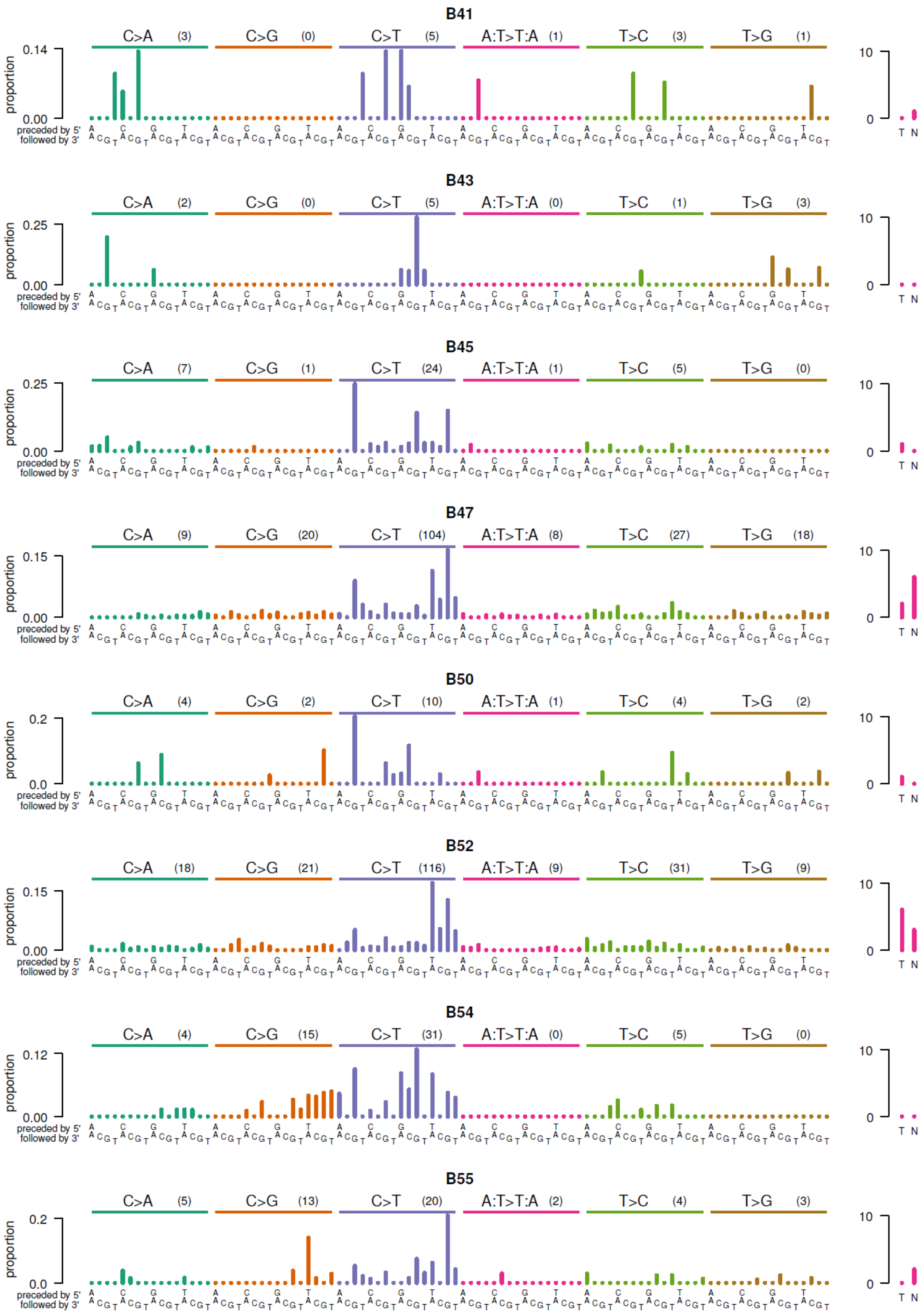

Supplementary Figure S3 continued. The mutation spectra of 99 bladder cancers from patients treated in China.

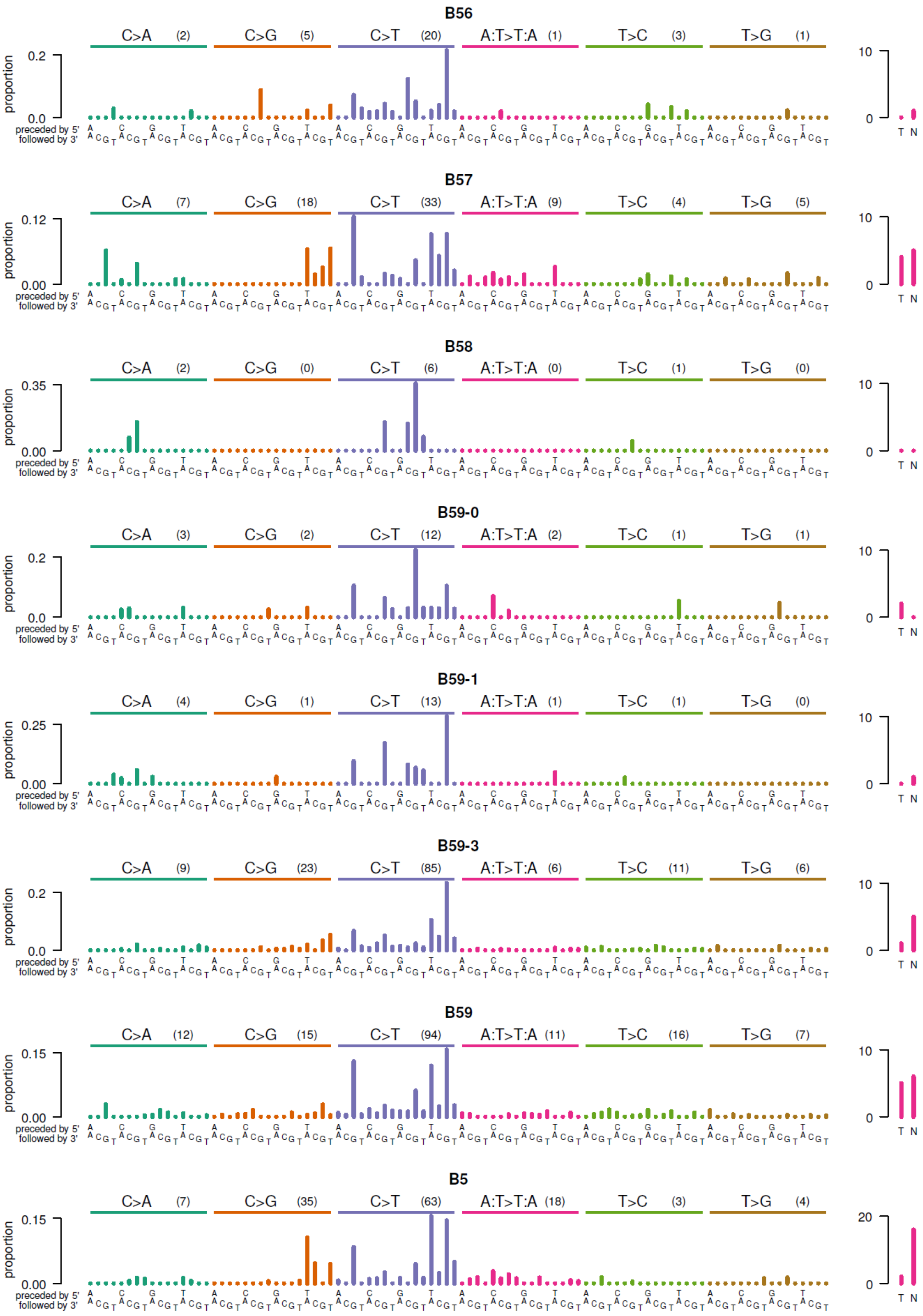

Supplementary Figure S3 continued. The mutation spectra of 99 bladder cancers from patients treated in China.

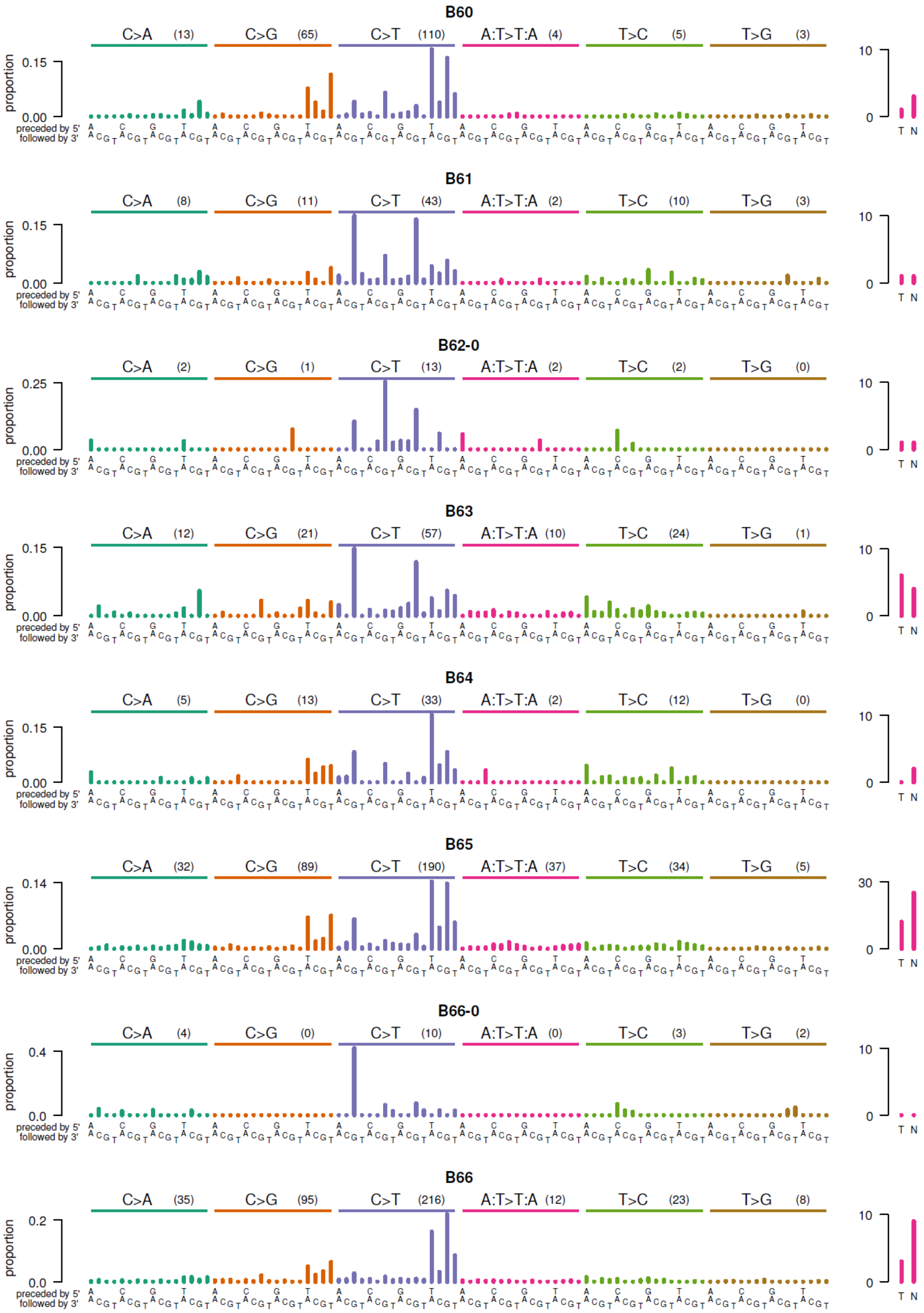

Supplementary Figure S3 continued. The mutation spectra of 99 bladder cancers from patients treated in China.

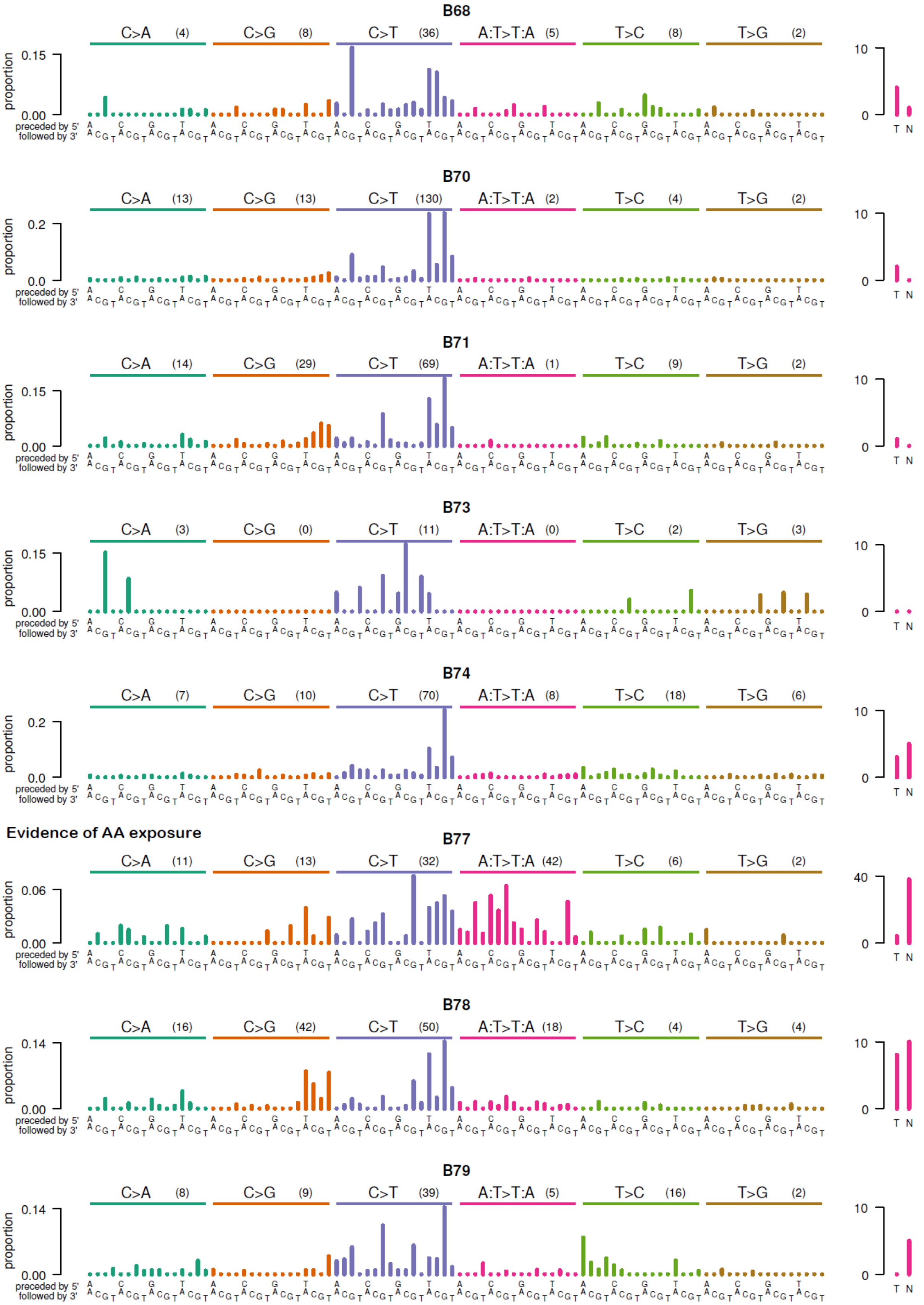

Supplementary Figure S3 continued. The mutation spectra of 99 bladder cancers from patients treated in China.

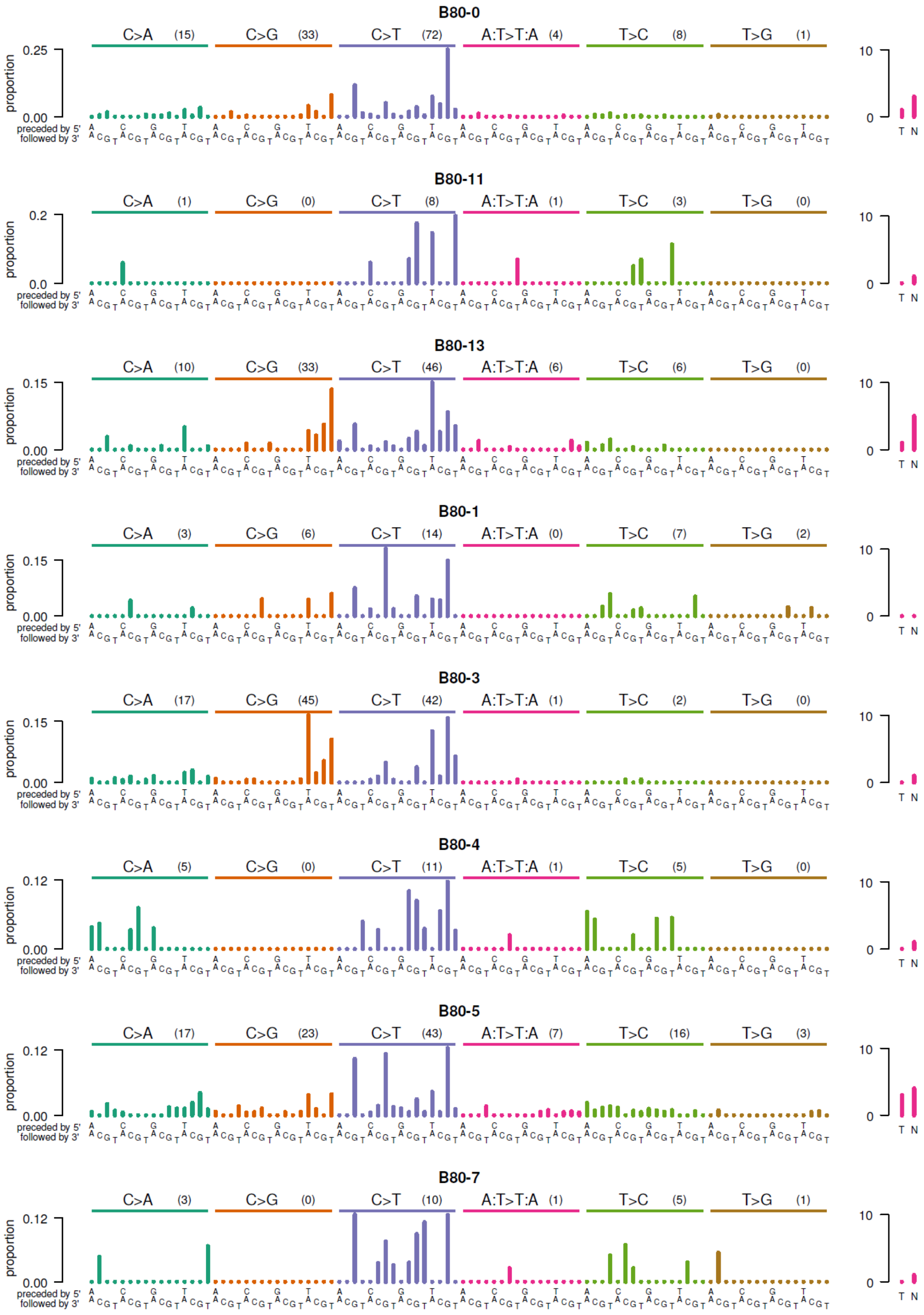

Supplementary Figure S3 continued. The mutation spectra of 99 bladder cancers from patients treated in China.

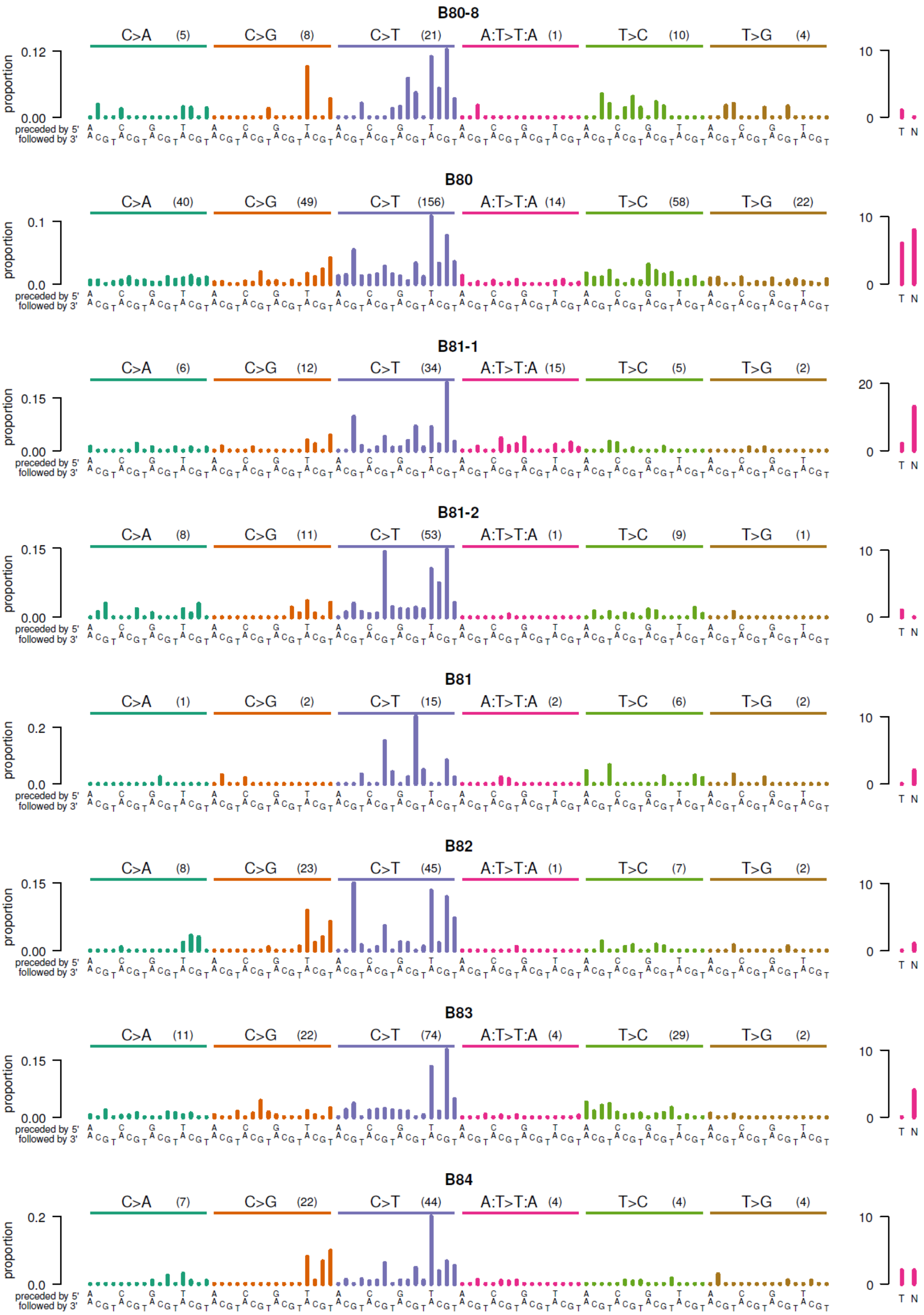

Supplementary Figure S3 continued. The mutation spectra of 99 bladder cancers from patients treated in China.

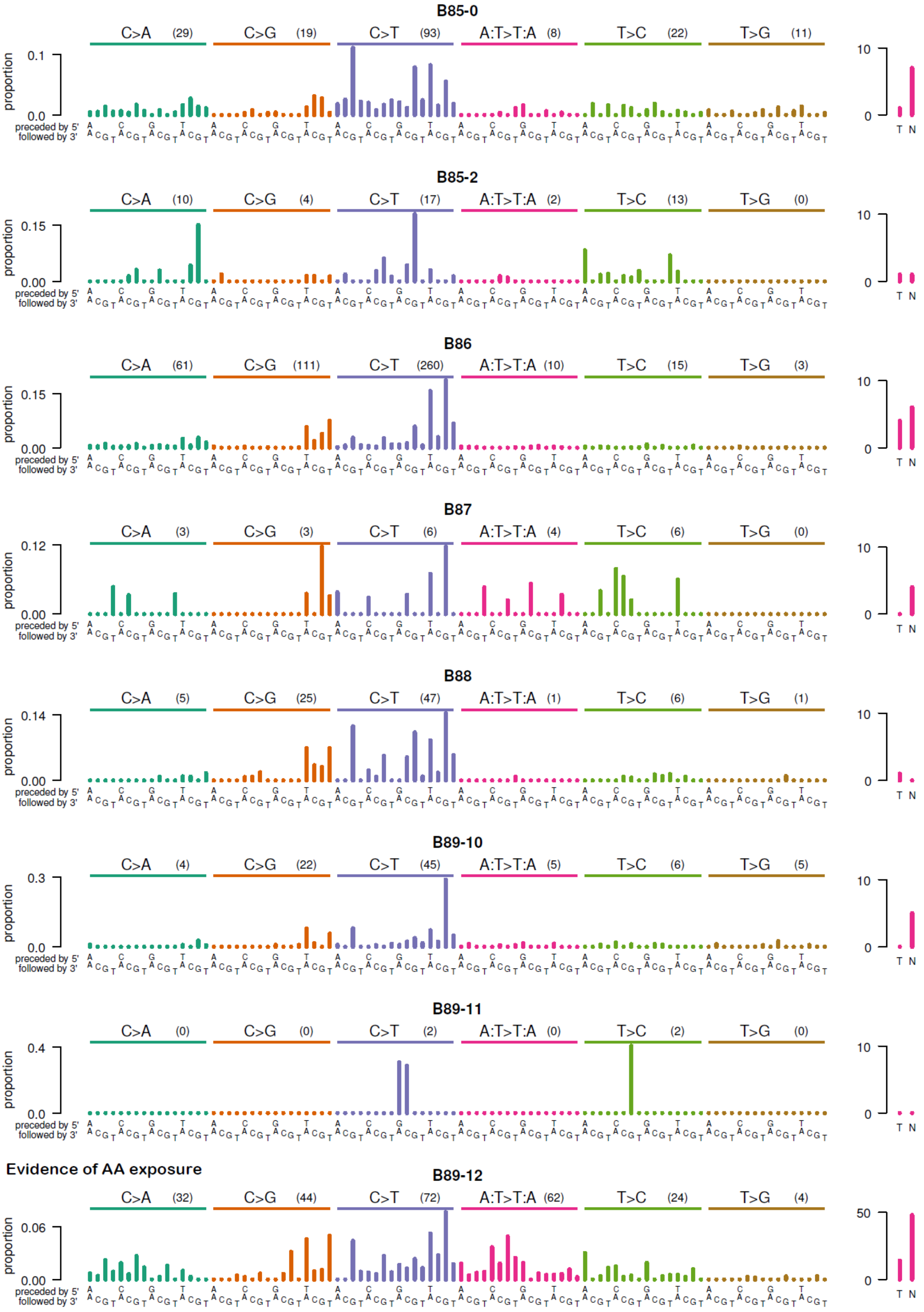

Supplementary Figure S3 continued. The mutation spectra of 99 bladder cancers from patients treated in China

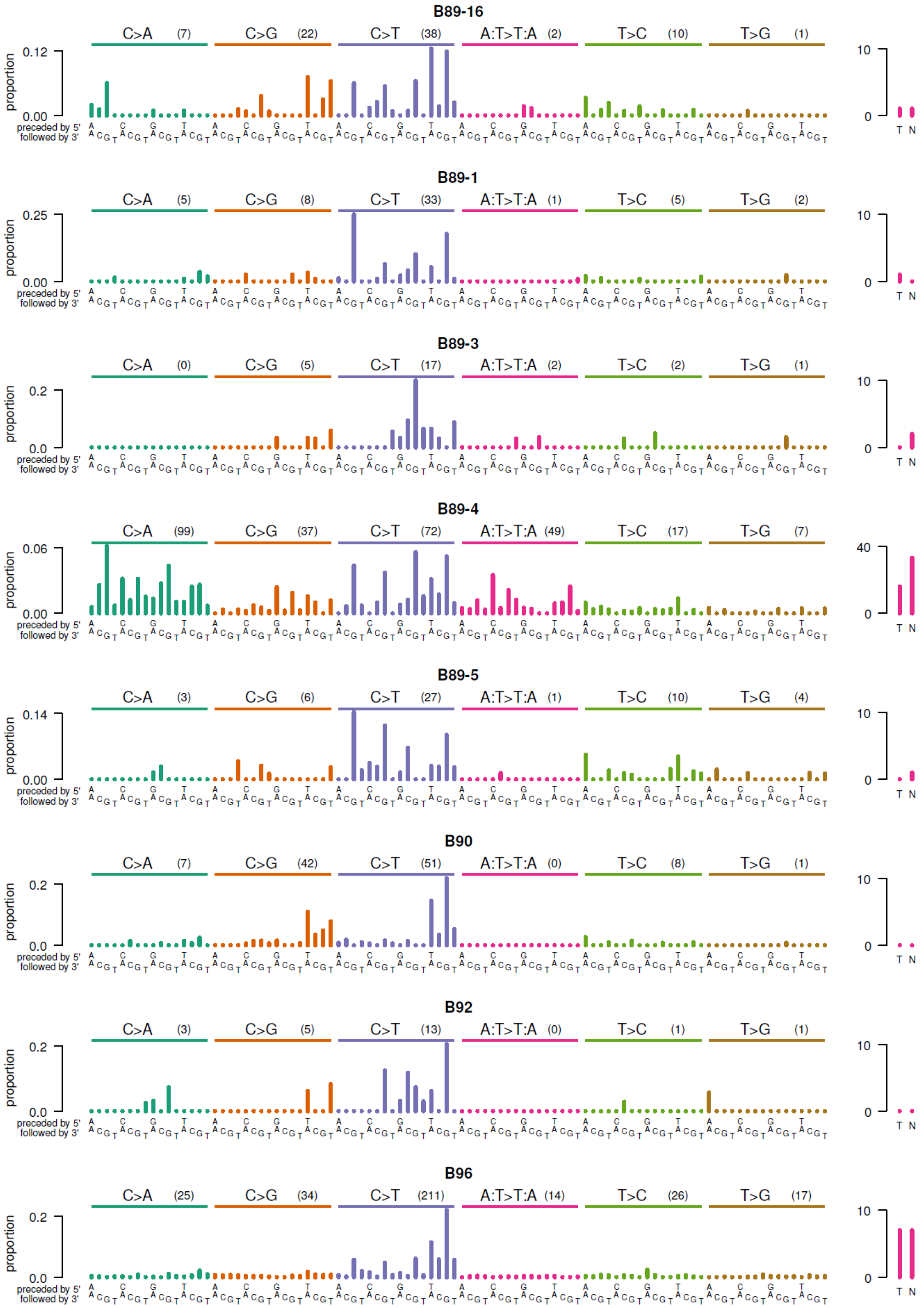

Supplementary Figure S3 continued. The mutation spectra of 99 bladder cancers from patients treated in China.

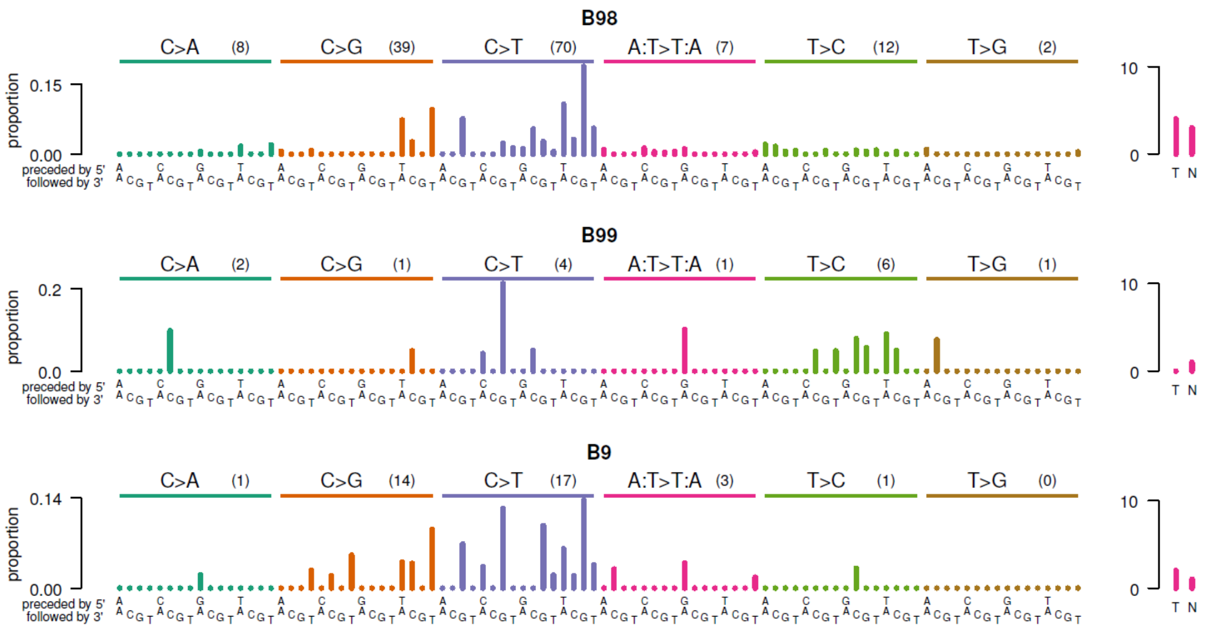

Supplementary Figure S3 continued. The mutation spectra of 99 bladder cancers from patients treated in China.

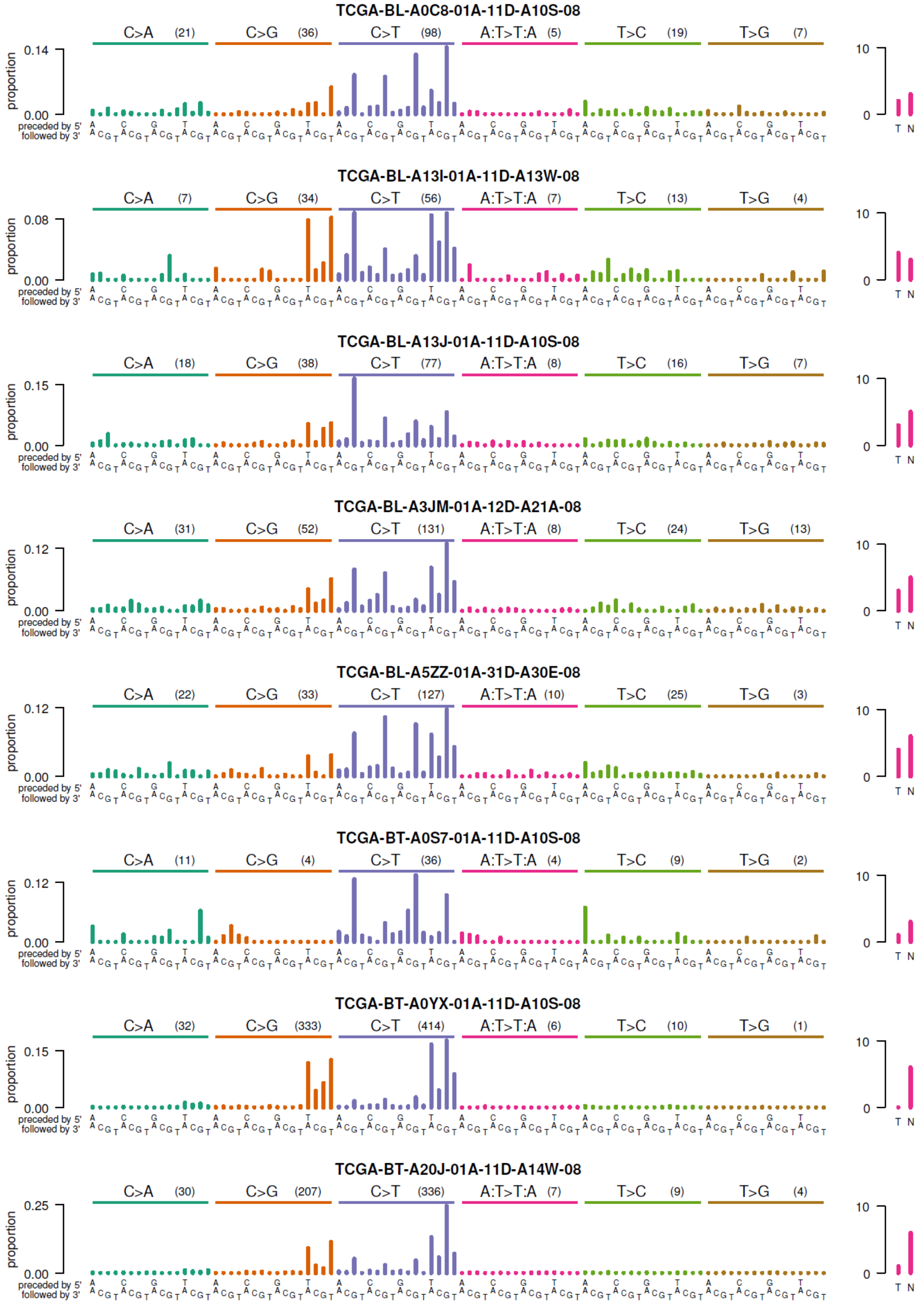

Supplementary Figure S4. The mutation spectra of 237 bladder cancers with data from TCGA [3].The somatic mutation data from 237 TCGA (http://cancergenome.nih.gov/) urothelial bladder tumors were downloaded from the TCGA data portal (https://tcga-data.nci.nih.gov/tcga/) on 8 May 2014.

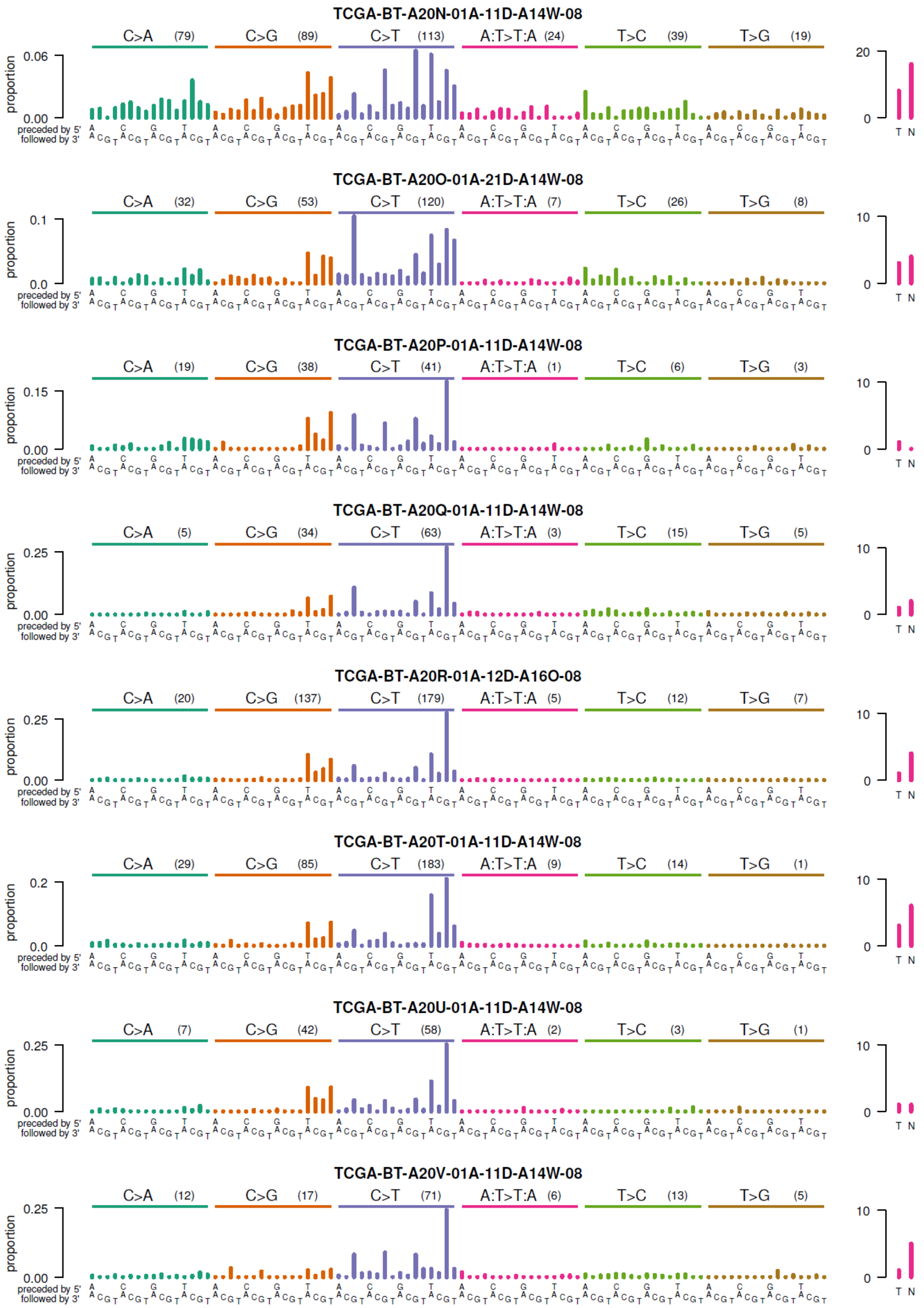

Supplementary Figure S4 continued. The mutation spectra of 237 bladder cancers with data from TCGA. The somatic mutation data from 237 TCGA (http://cancergenome.nih.gov/) urothelial bladder tumors were downloaded from the TCGA data portal (https://tcga-data.nci.nih.gov/tcga/) on 8 May 2014.

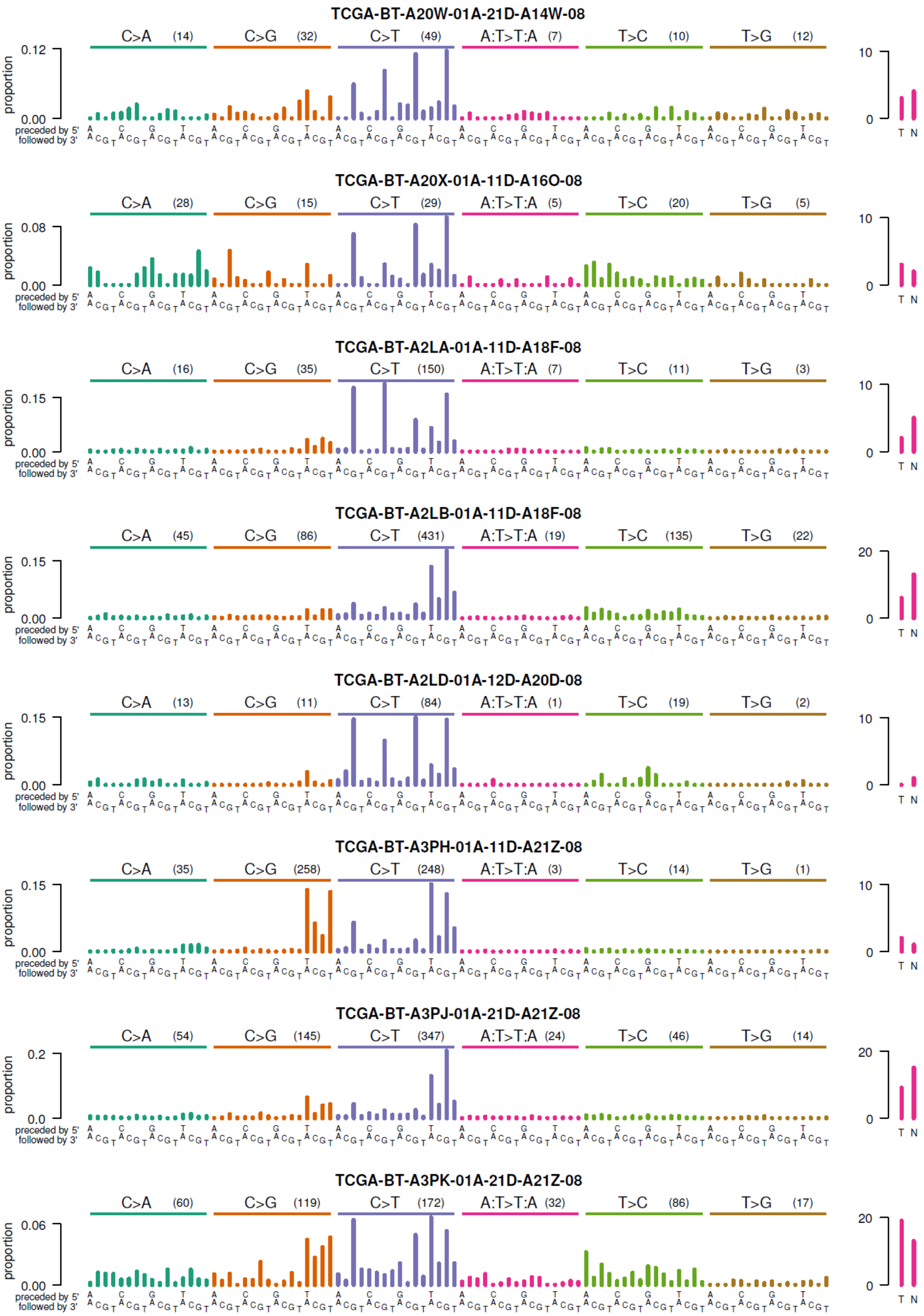

Supplementary Figure S4 continued. The mutation spectra of 237 bladder cancers with data from TCGA. The somatic mutation data from 237 TCGA (http://cancergenome.nih.gov/) urothelial bladder tumors were downloaded from the TCGA data portal (https://tcga-data.nci.nih.gov/tcga/) on 8 May 2014.

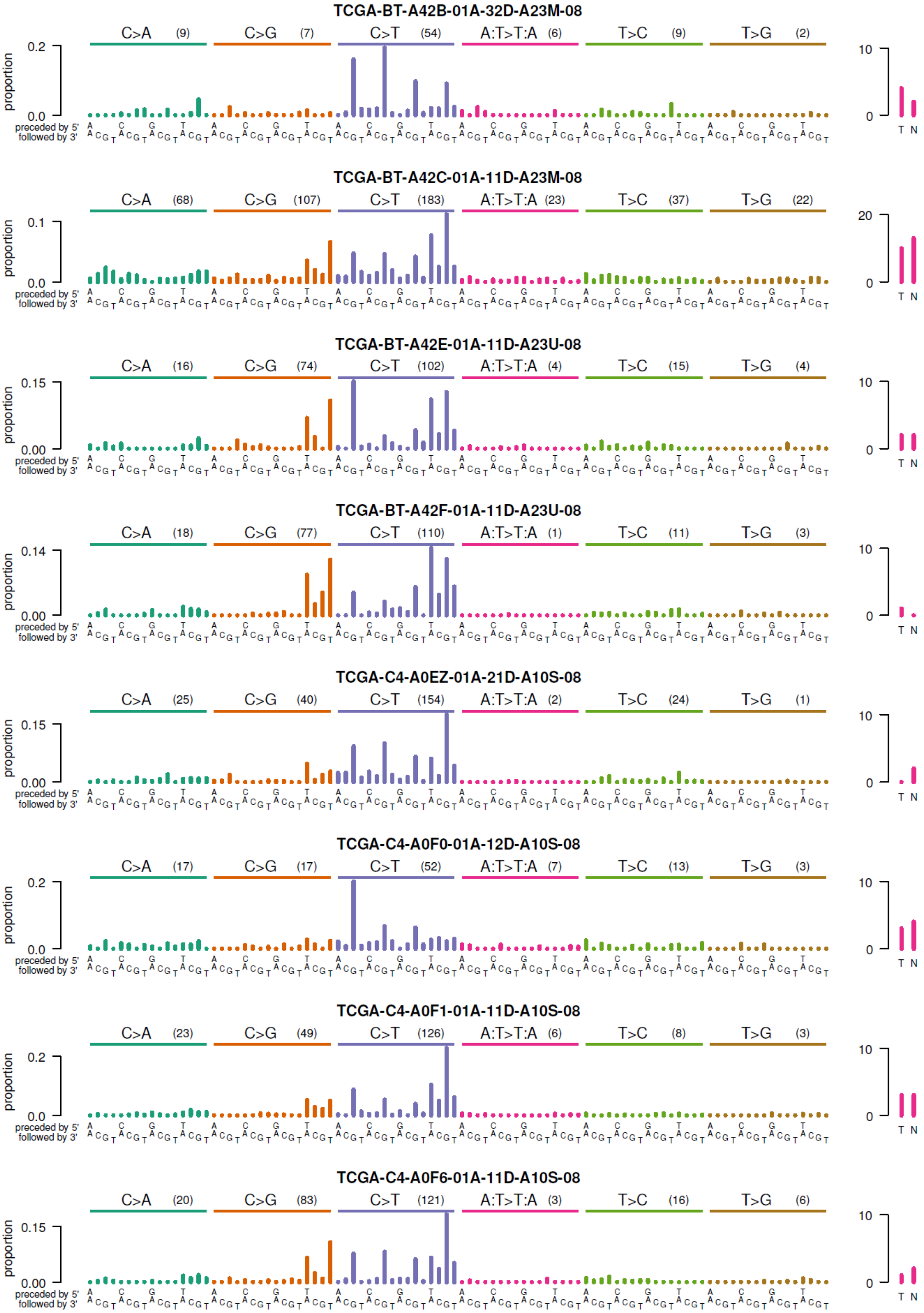

Supplementary Figure S4 continued. The mutation spectra of 237 bladder cancers with data from TCGA. The somatic mutation data from 237 TCGA (http://cancergenome.nih.gov/) urothelial bladder tumors were downloaded from the TCGA data portal (https://tcga-data.nci.nih.gov/tcga/) on 8 May 2014.

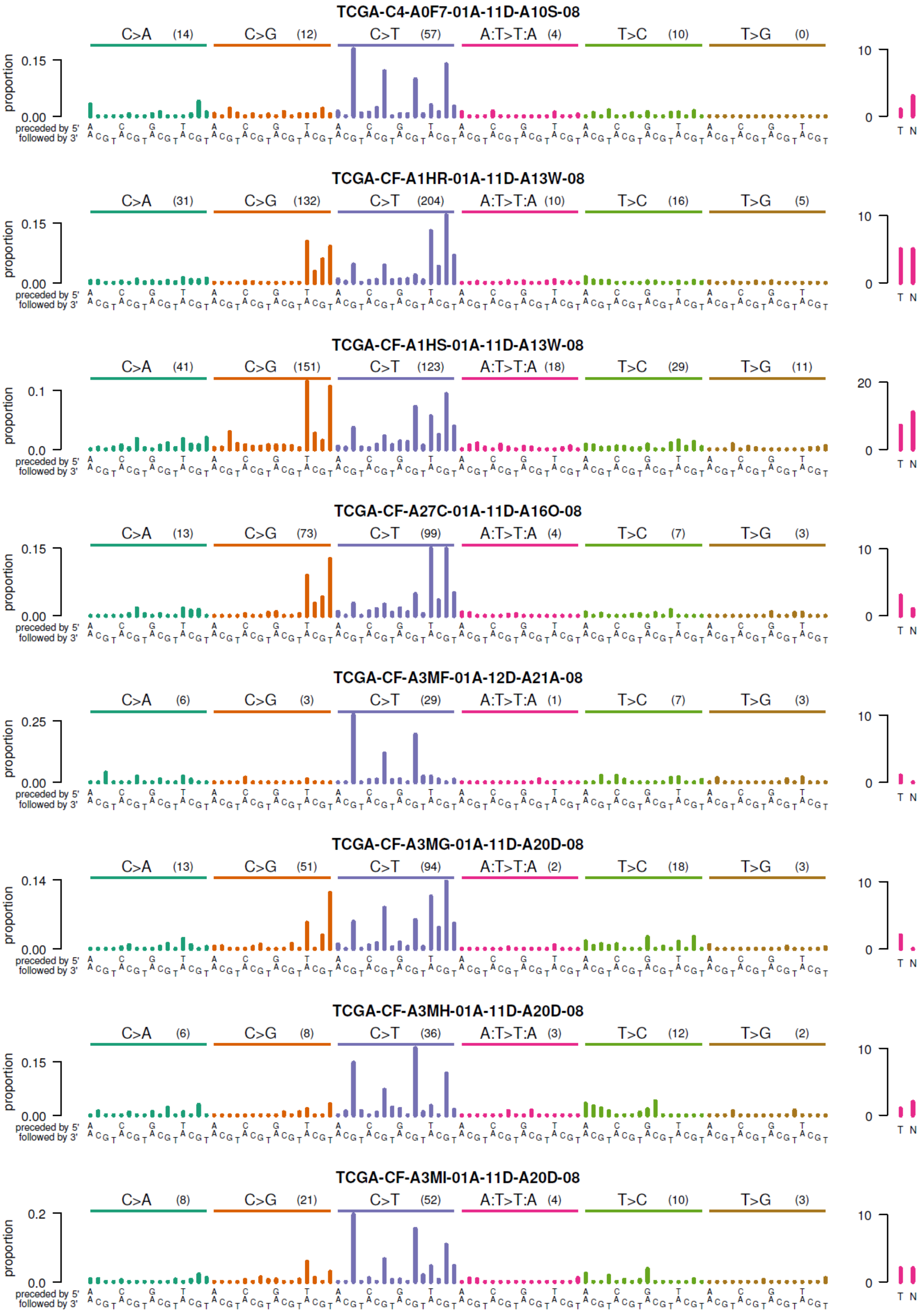

Supplementary Figure S4 continued. The mutation spectra of 237 bladder cancers with data from TCGA. The somatic mutation data from 237 TCGA (http://cancergenome.nih.gov/) urothelial bladder tumors were downloaded from the TCGA data portal (https://tcga-data.nci.nih.gov/tcga/) on 8 May 2014.

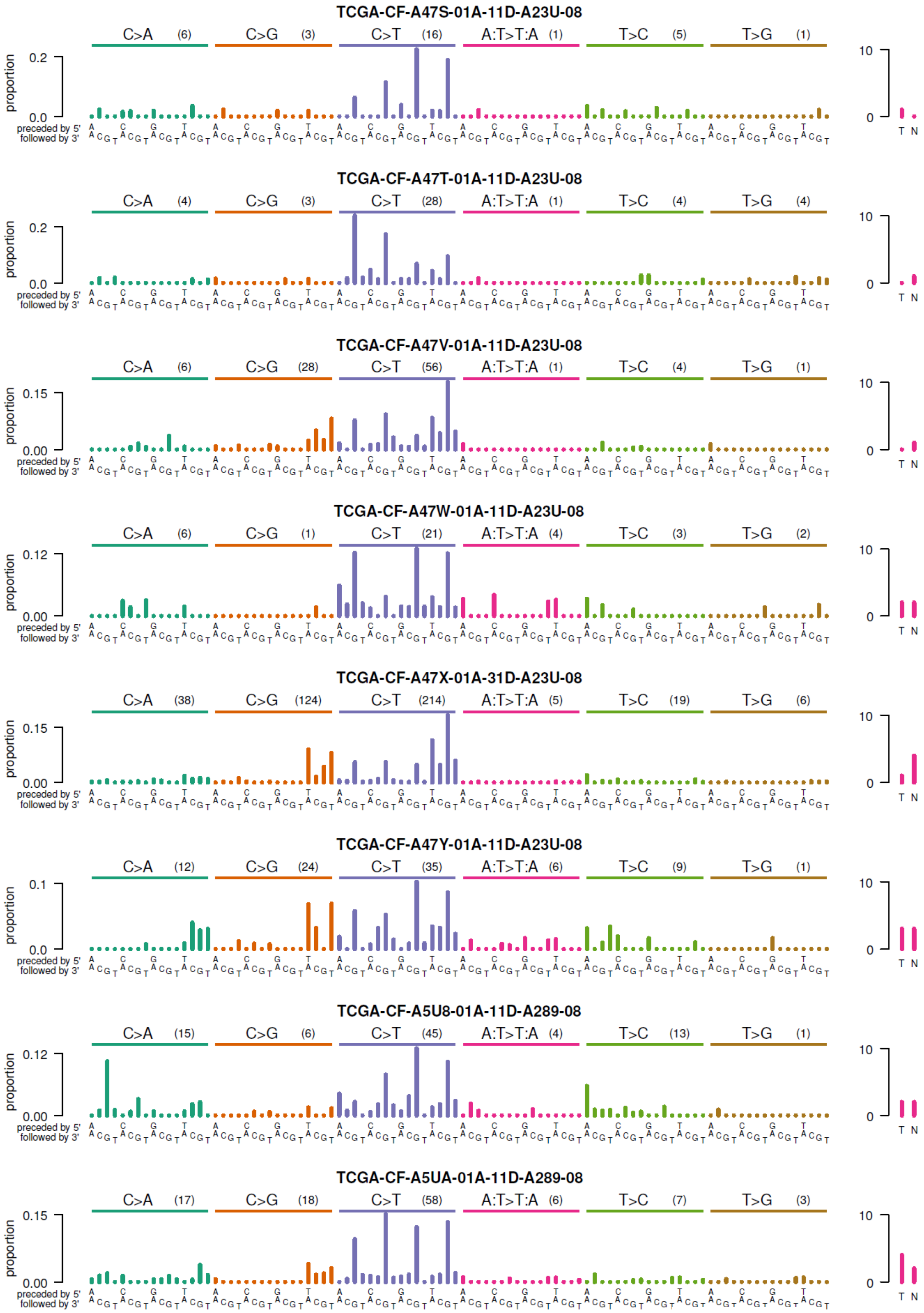

Supplementary Figure S4 continued. The mutation spectra of 237 bladder cancers with data from TCGA. The somatic mutation data from 237 TCGA (http://cancergenome.nih.gov/) urothelial bladder tumors were downloaded from the TCGA data portal (https://tcga-data.nci.nih.gov/tcga/) on 8 May 2014.

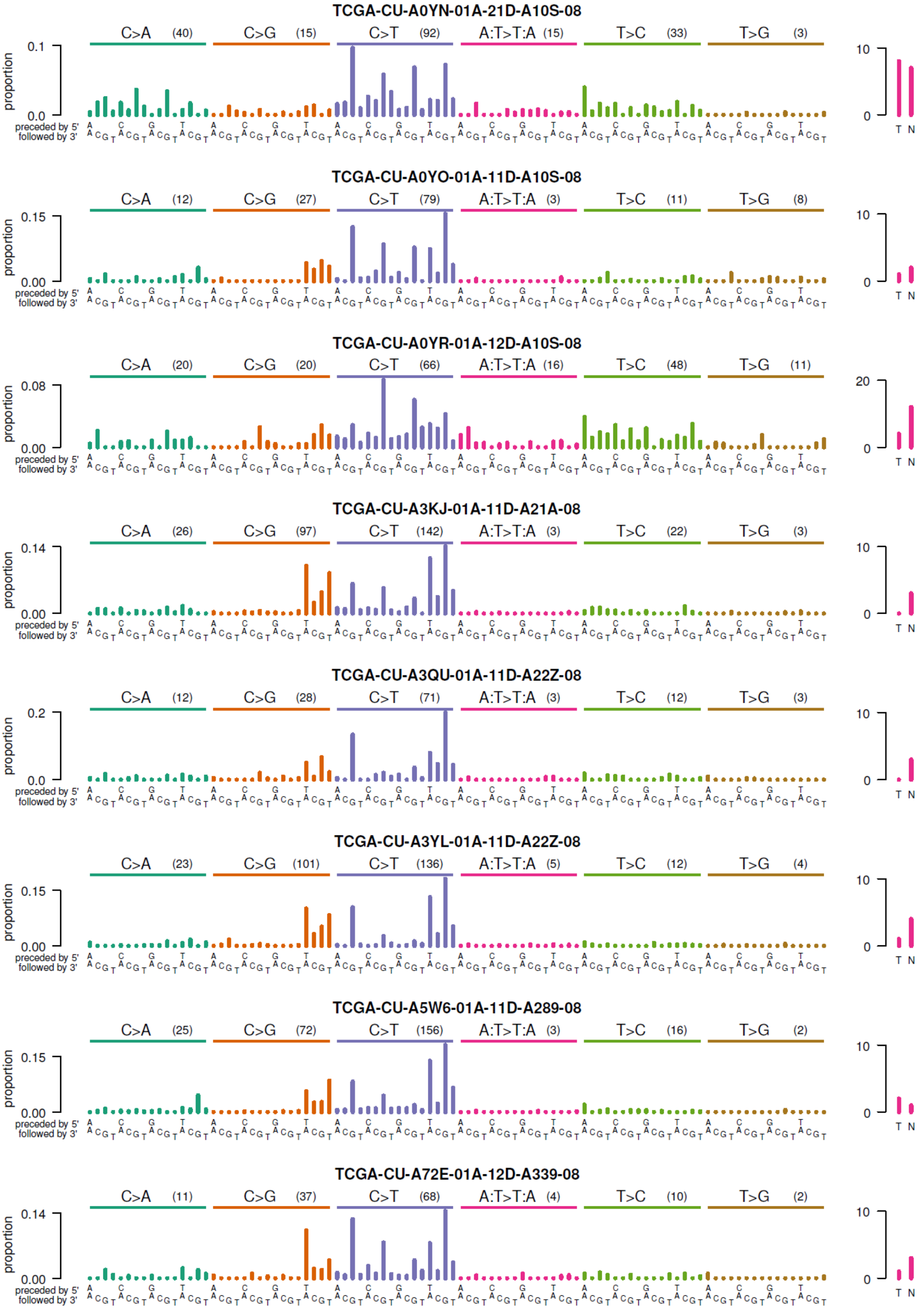

Supplementary Figure S4 continued. The mutation spectra of 237 bladder cancers with data from TCGA. The somatic mutation data from 237 TCGA (http://cancergenome.nih.gov/) urothelial bladder tumors were downloaded from the TCGA data portal (https://tcga-data.nci.nih.gov/tcga/) on 8 May 2014.

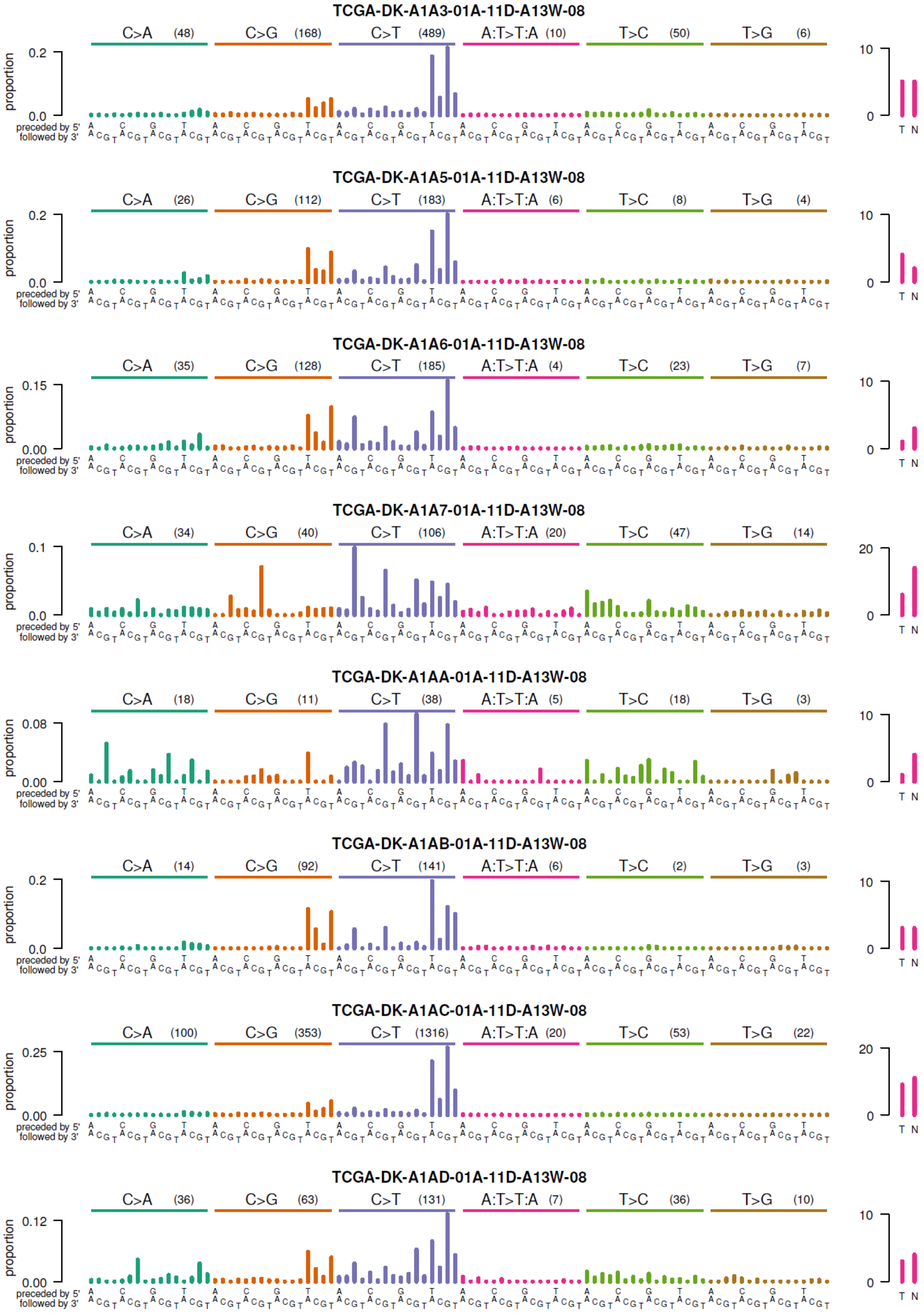

Supplementary Figure S4 continued. The mutation spectra of 237 bladder cancers with data from TCGA. The somatic mutation data from 237 TCGA (http://cancergenome.nih.gov/) urothelial bladder tumors were downloaded from the TCGA data portal (https://tcga-data.nci.nih.gov/tcga/) on 8 May 2014.

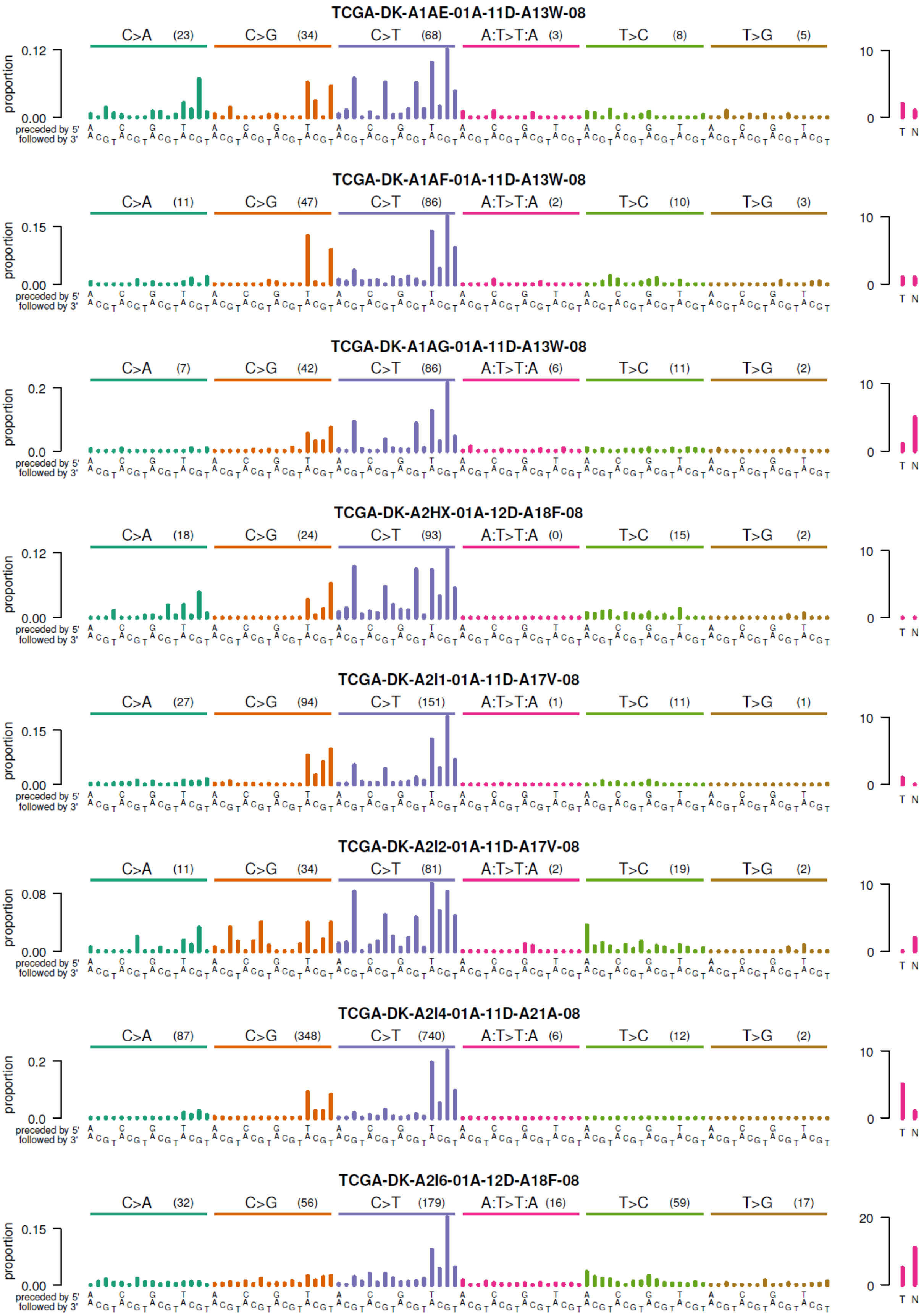

Supplementary Figure S4 continued. The mutation spectra of 237 bladder cancers with data from TCGA. The somatic mutation data from 237 TCGA (http://cancergenome.nih.gov/) urothelial bladder tumors were downloaded from the TCGA data portal (https://tcga-data.nci.nih.gov/tcga/) on 8 May 2014.

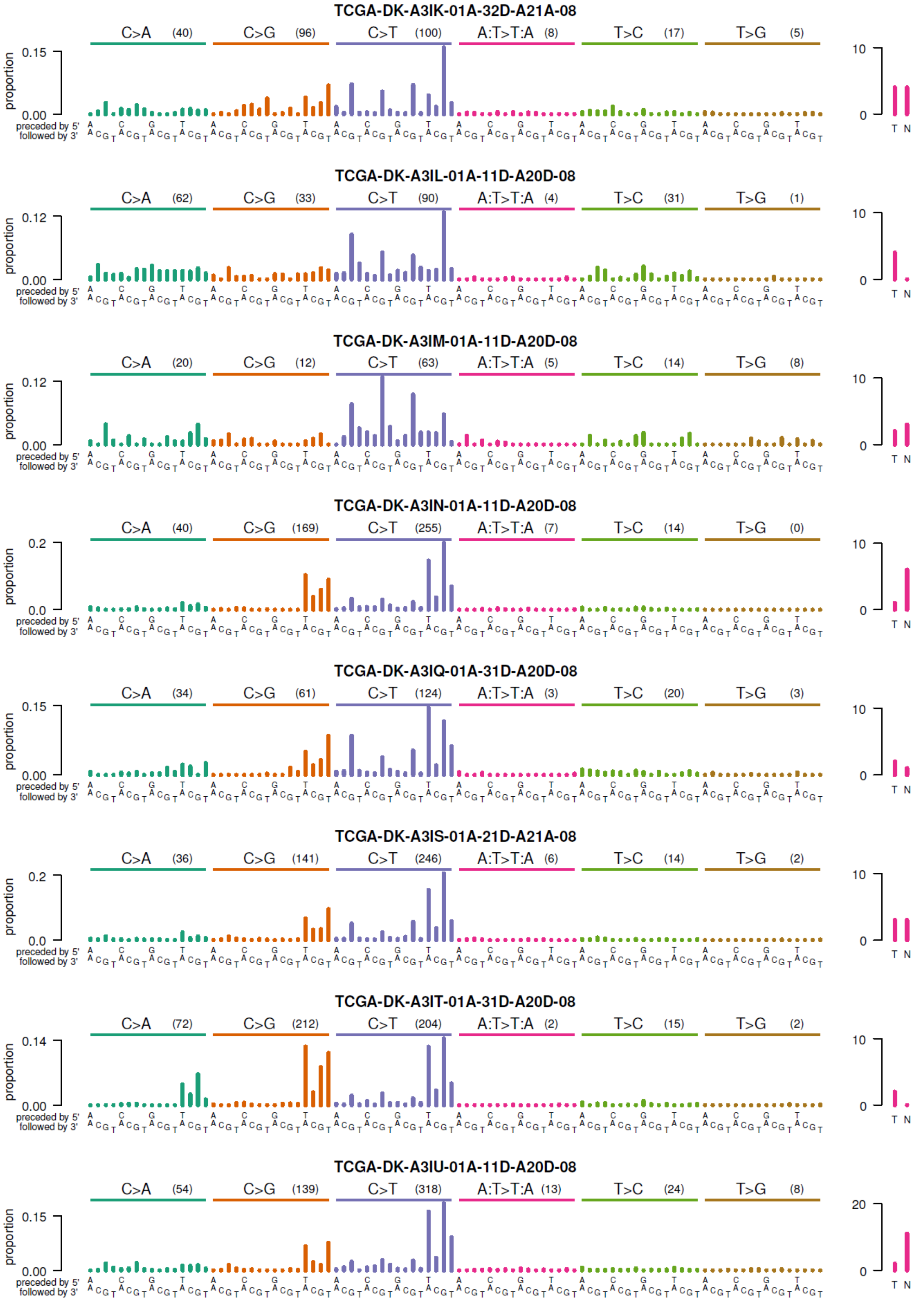

Supplementary Figure S4 continued. The mutation spectra of 237 bladder cancers with data from TCGA. The somatic mutation data from 237 TCGA (http://cancergenome.nih.gov/) urothelial bladder tumors were downloaded from the TCGA data portal (https://tcga-data.nci.nih.gov/tcga/) on 8 May 2014.

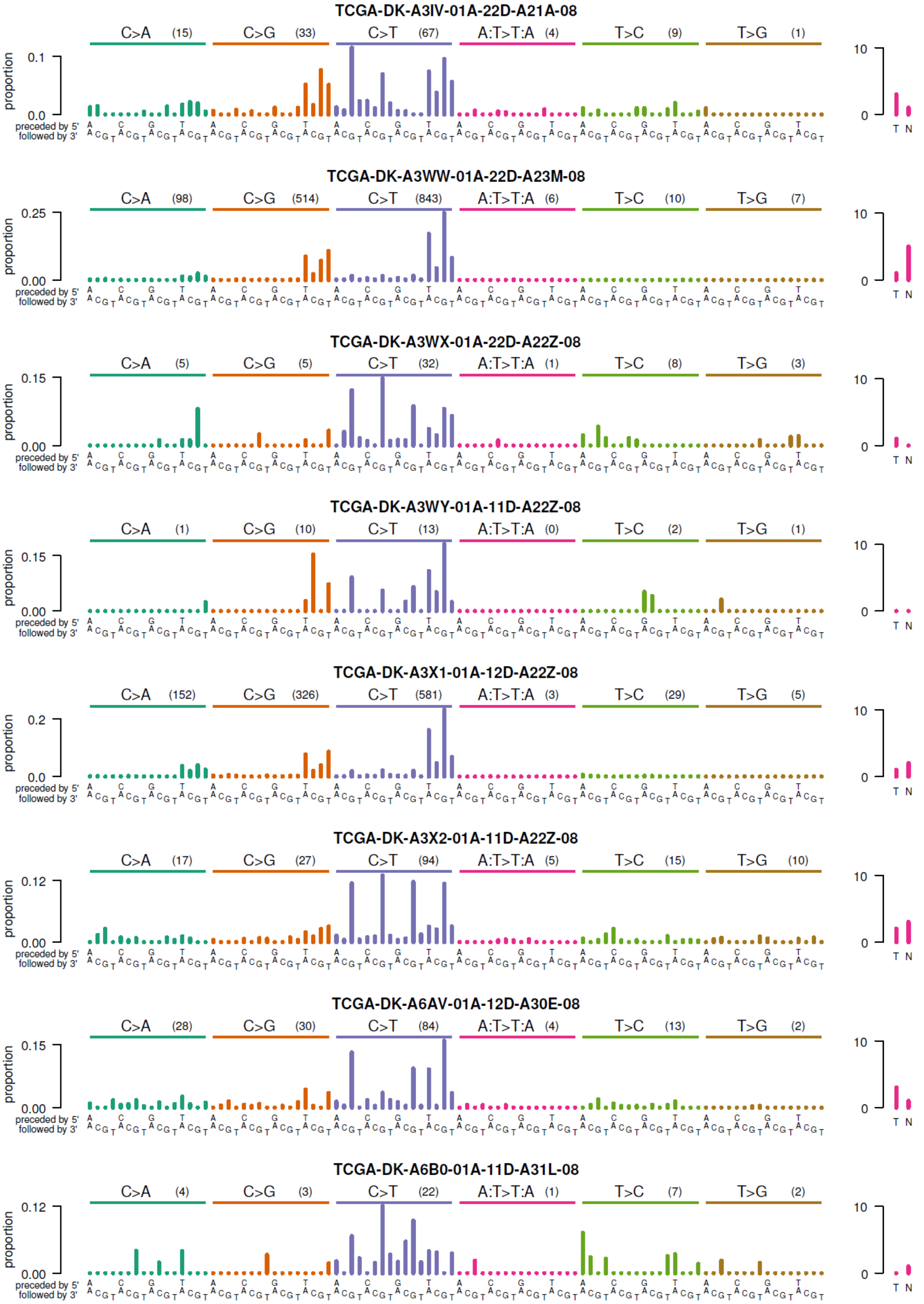

Supplementary Figure S4 continued. The mutation spectra of 237 bladder cancers with data from TCGA. The somatic mutation data from 237 TCGA (http://cancergenome.nih.gov/) urothelial bladder tumors were downloaded from the TCGA data portal (https://tcga-data.nci.nih.gov/tcga/) on 8 May 2014.

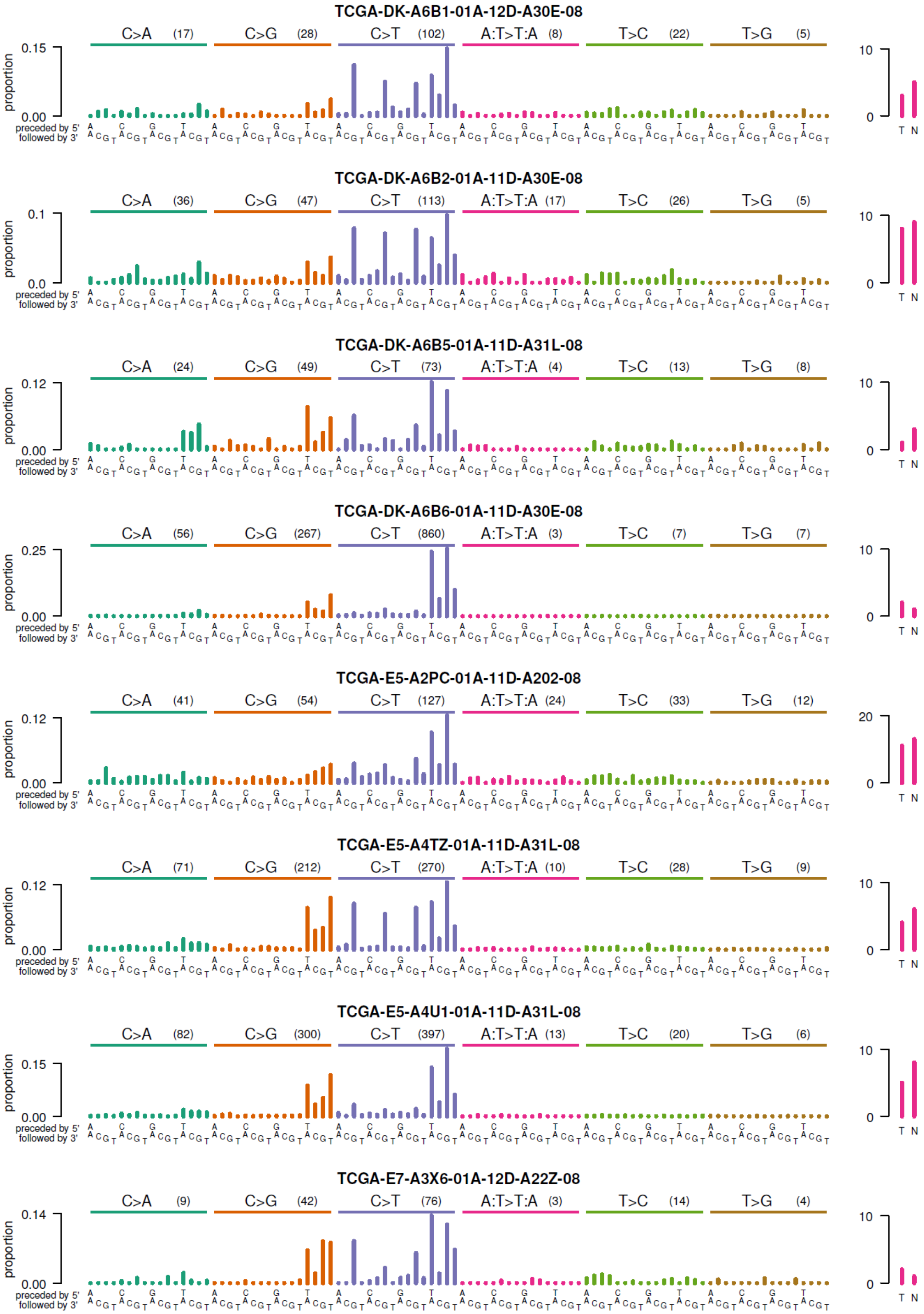

Supplementary Figure S4 continued. The mutation spectra of 237 bladder cancers with data from TCGA. The somatic mutation data from 237 TCGA (http://cancergenome.nih.gov/) urothelial bladder tumors were downloaded from the TCGA data portal (https://tcga-data.nci.nih.gov/tcga/) on 8 May 2014.

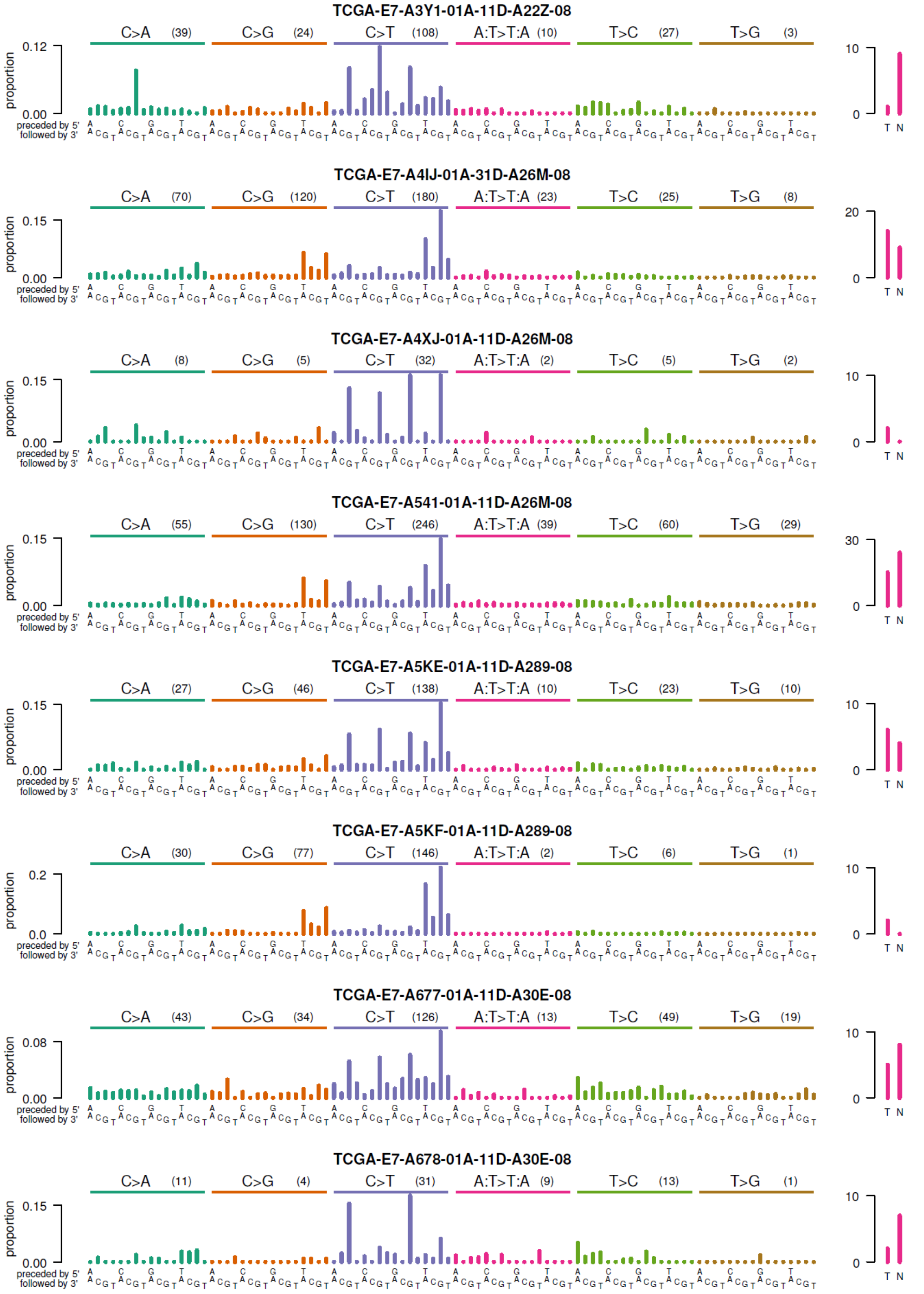

Supplementary Figure S4 continued. The mutation spectra of 237 bladder cancers with data from TCGA. The somatic mutation data from 237 TCGA (http://cancergenome.nih.gov/) urothelial bladder tumors were downloaded from the TCGA data portal (https://tcga-data.nci.nih.gov/tcga/) on 8 May 2014.

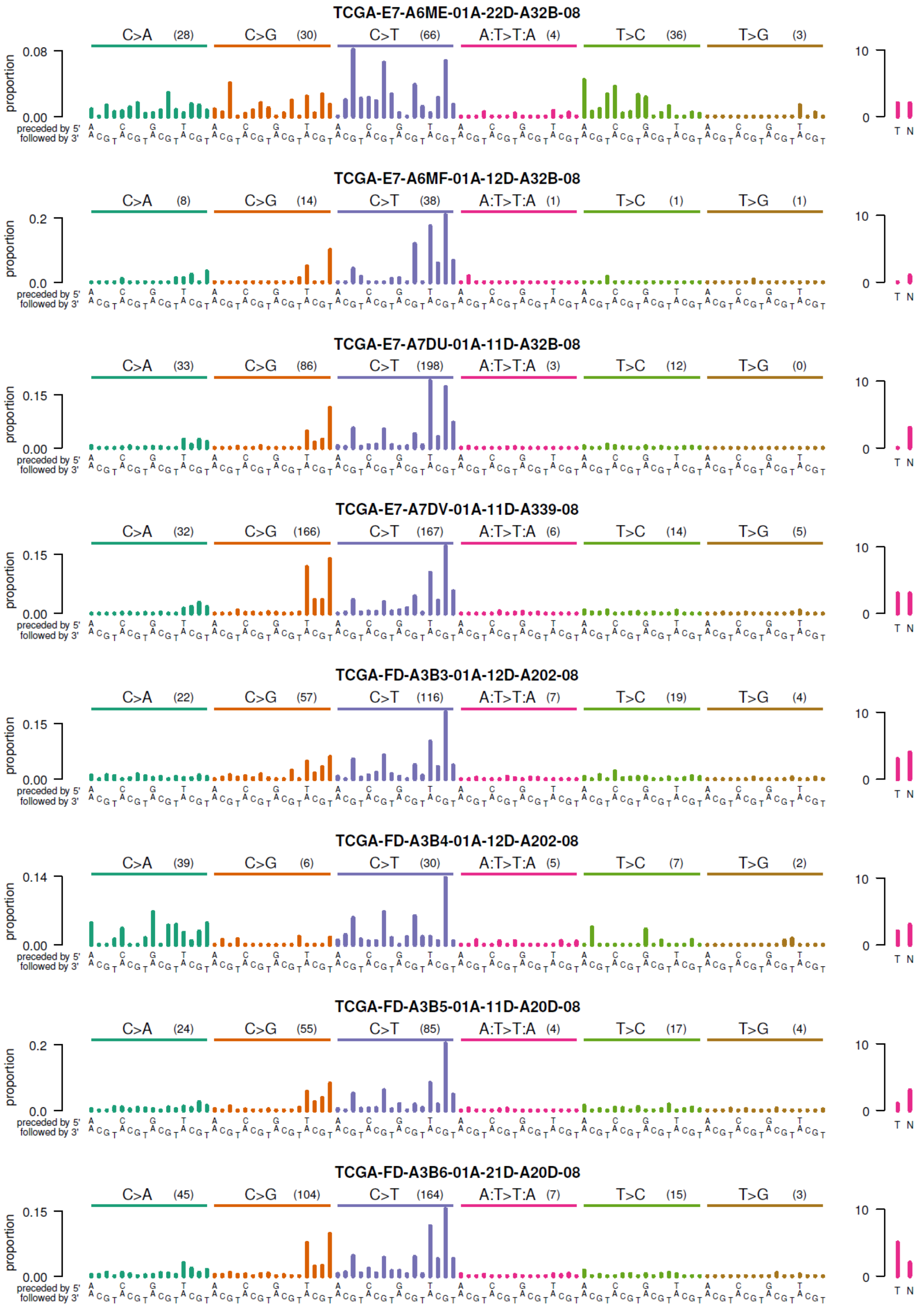

Supplementary Figure S4 continued. The mutation spectra of 237 bladder cancers with data from TCGA. The somatic mutation data from 237 TCGA (http://cancergenome.nih.gov/) urothelial bladder tumors were downloaded from the TCGA data portal (https://tcga-data.nci.nih.gov/tcga/) on 8 May 2014.

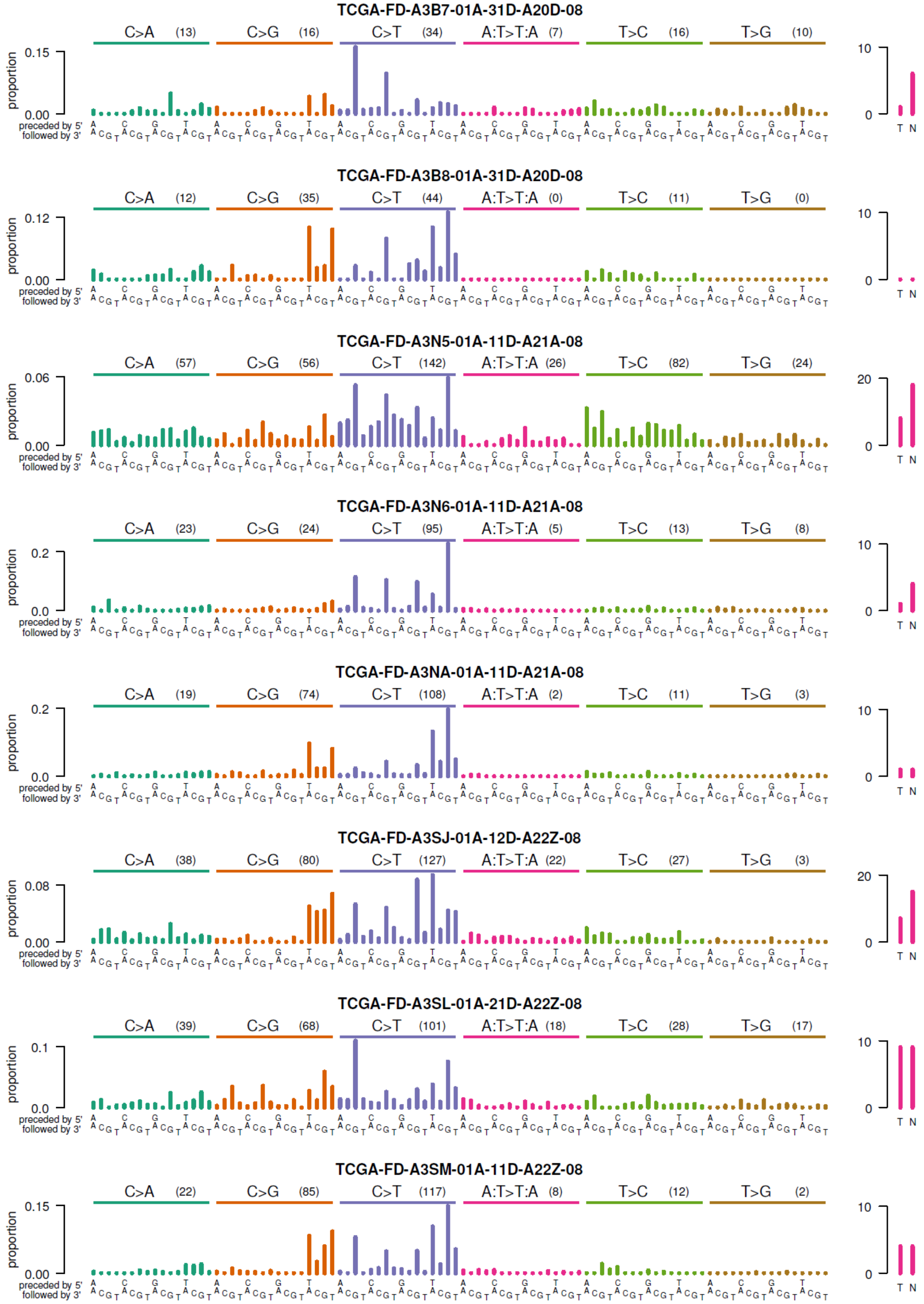

Supplementary Figure S4 continued. The mutation spectra of 237 bladder cancers with data from TCGA. The somatic mutation data from 237 TCGA (http://cancergenome.nih.gov/) urothelial bladder tumors were downloaded from the TCGA data portal (https://tcga-data.nci.nih.gov/tcga/) on 8 May 2014.

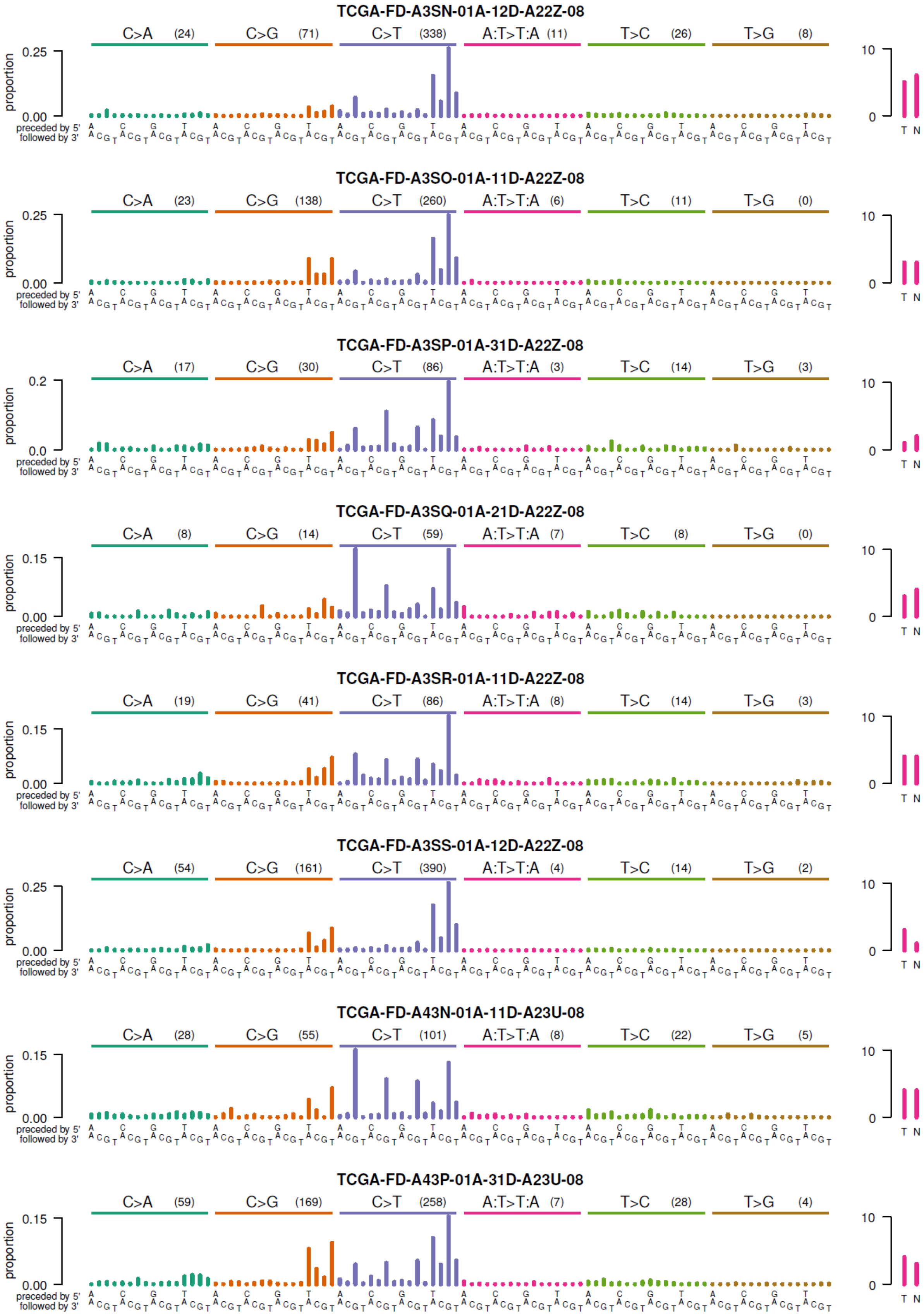

Supplementary Figure S4 continued. The mutation spectra of 237 bladder cancers with data from TCGA. The somatic mutation data from 237 TCGA (http://cancergenome.nih.gov/) urothelial bladder tumors were downloaded from the TCGA data portal (https://tcga-data.nci.nih.gov/tcga/) on 8 May 2014.

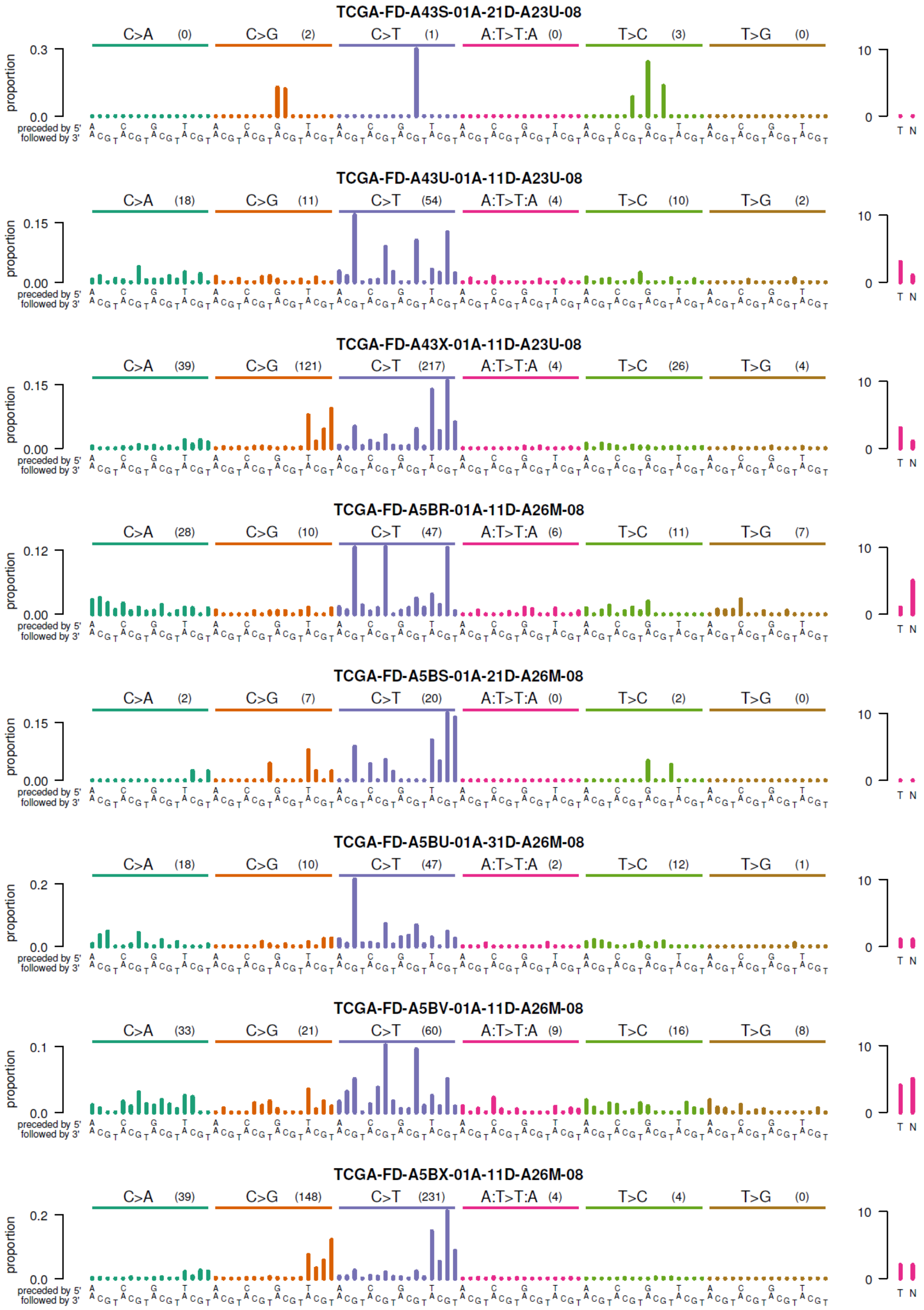

Supplementary Figure S4 continued. The mutation spectra of 237 bladder cancers with data from TCGA. The somatic mutation data from 237 TCGA (http://cancergenome.nih.gov/) urothelial bladder tumors were downloaded from the TCGA data portal (https://tcga-data.nci.nih.gov/tcga/) on 8 May 2014.

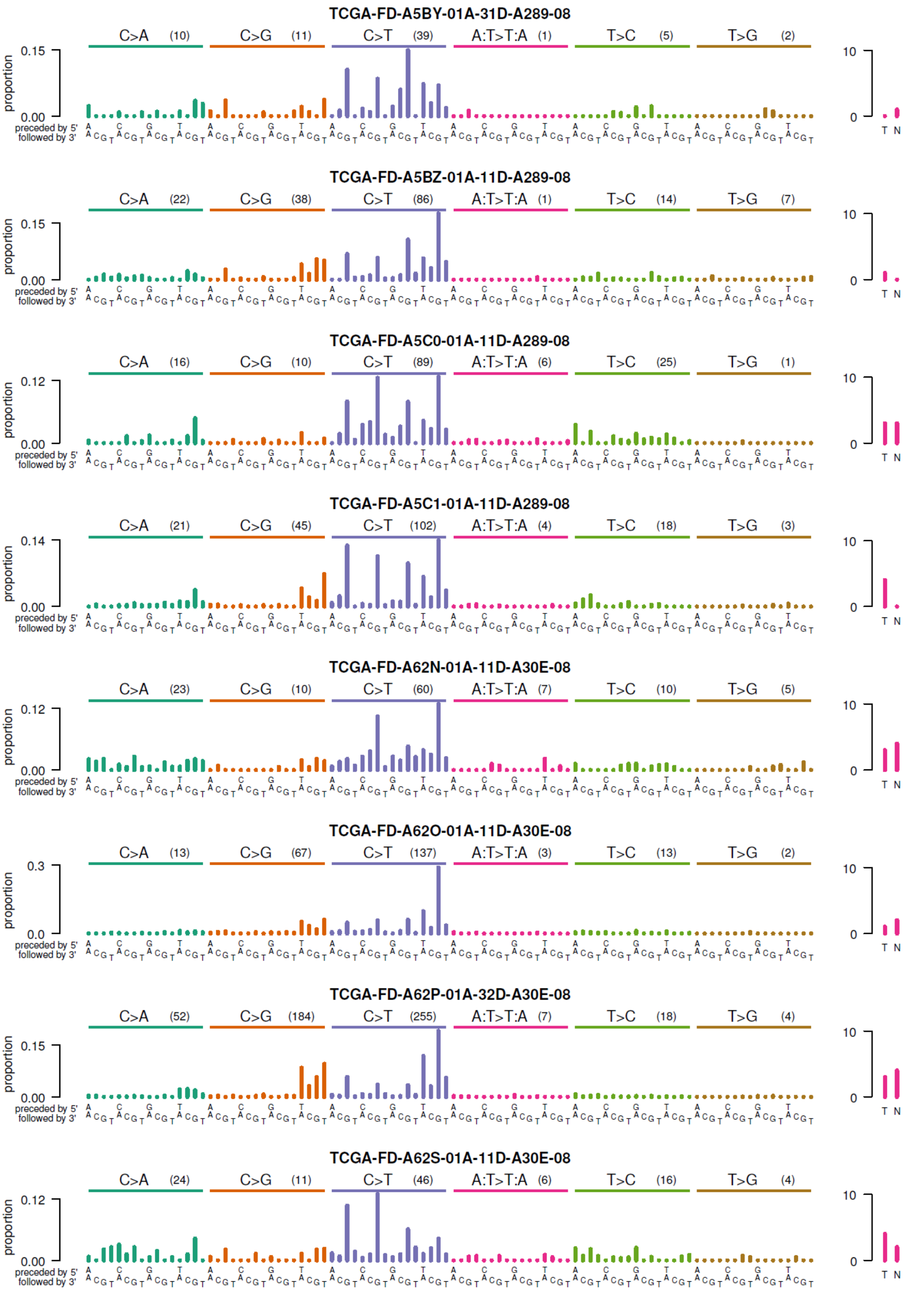

Supplementary Figure S4 continued. The mutation spectra of 237 bladder cancers with data from TCGA. The somatic mutation data from 237 TCGA (http://cancergenome.nih.gov/) urothelial bladder tumors were downloaded from the TCGA data portal (https://tcga-data.nci.nih.gov/tcga/) on 8 May 2014.

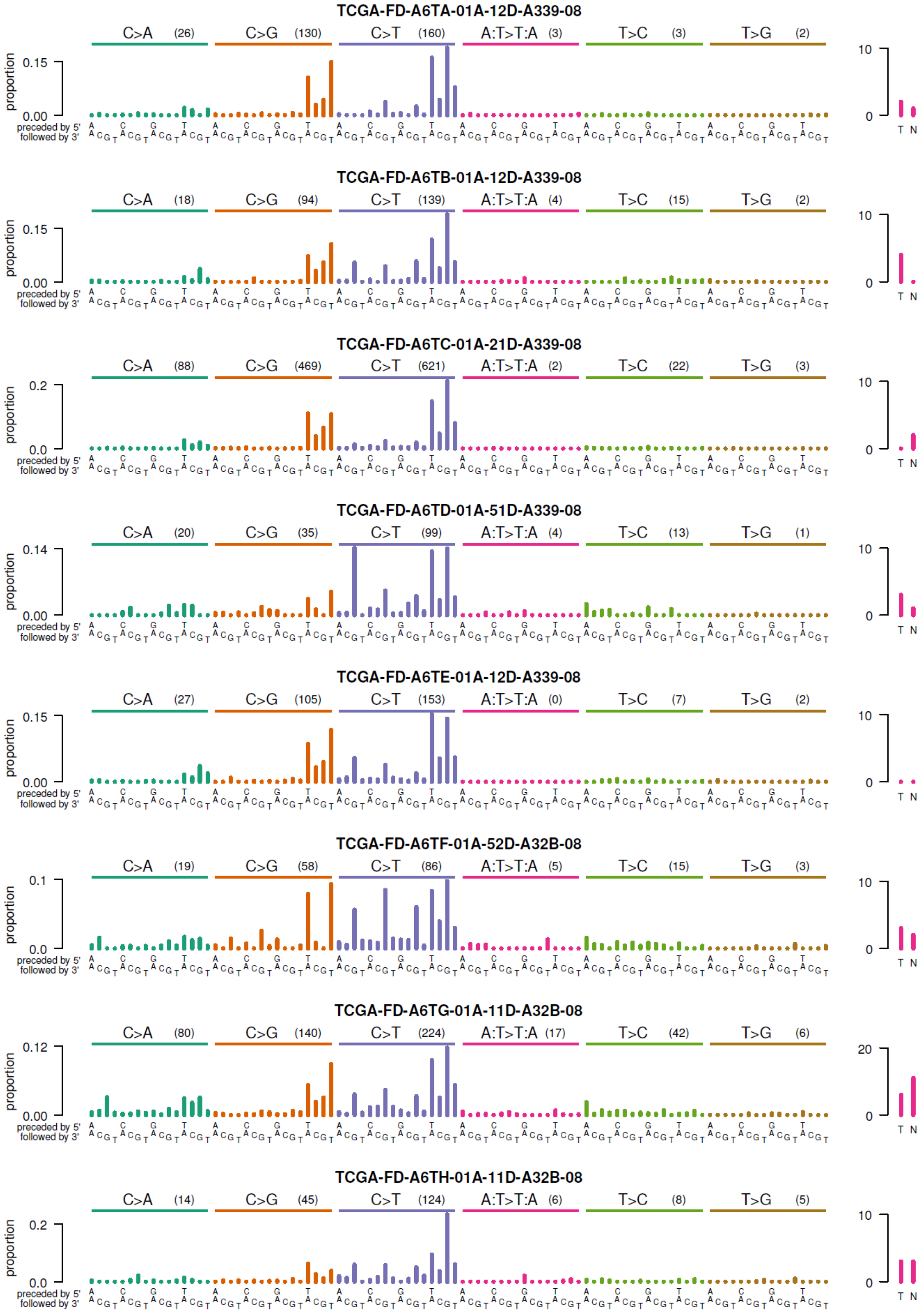

Supplementary Figure S4 continued. The mutation spectra of 237 bladder cancers with data from TCGA. The somatic mutation data from 237 TCGA (http://cancergenome.nih.gov/) urothelial bladder tumors were downloaded from the TCGA data portal (https://tcga-data.nci.nih.gov/tcga/) on 8 May 2014.

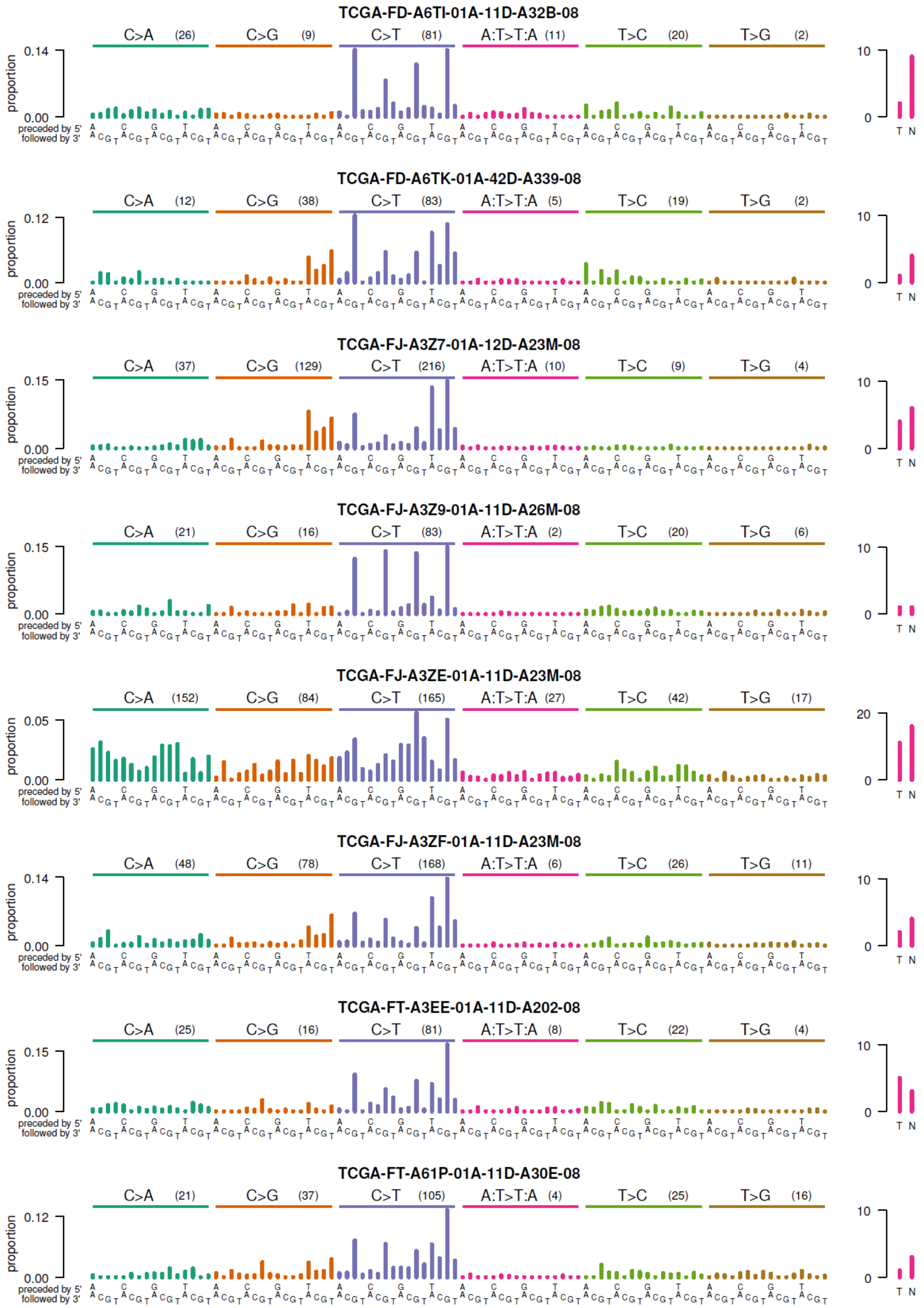

Supplementary Figure S4 continued. The mutation spectra of 237 bladder cancers with data from TCGA. The somatic mutation data from 237 TCGA (http://cancergenome.nih.gov/) urothelial bladder tumors were downloaded from the TCGA data portal (https://tcga-data.nci.nih.gov/tcga/) on 8 May 2014.

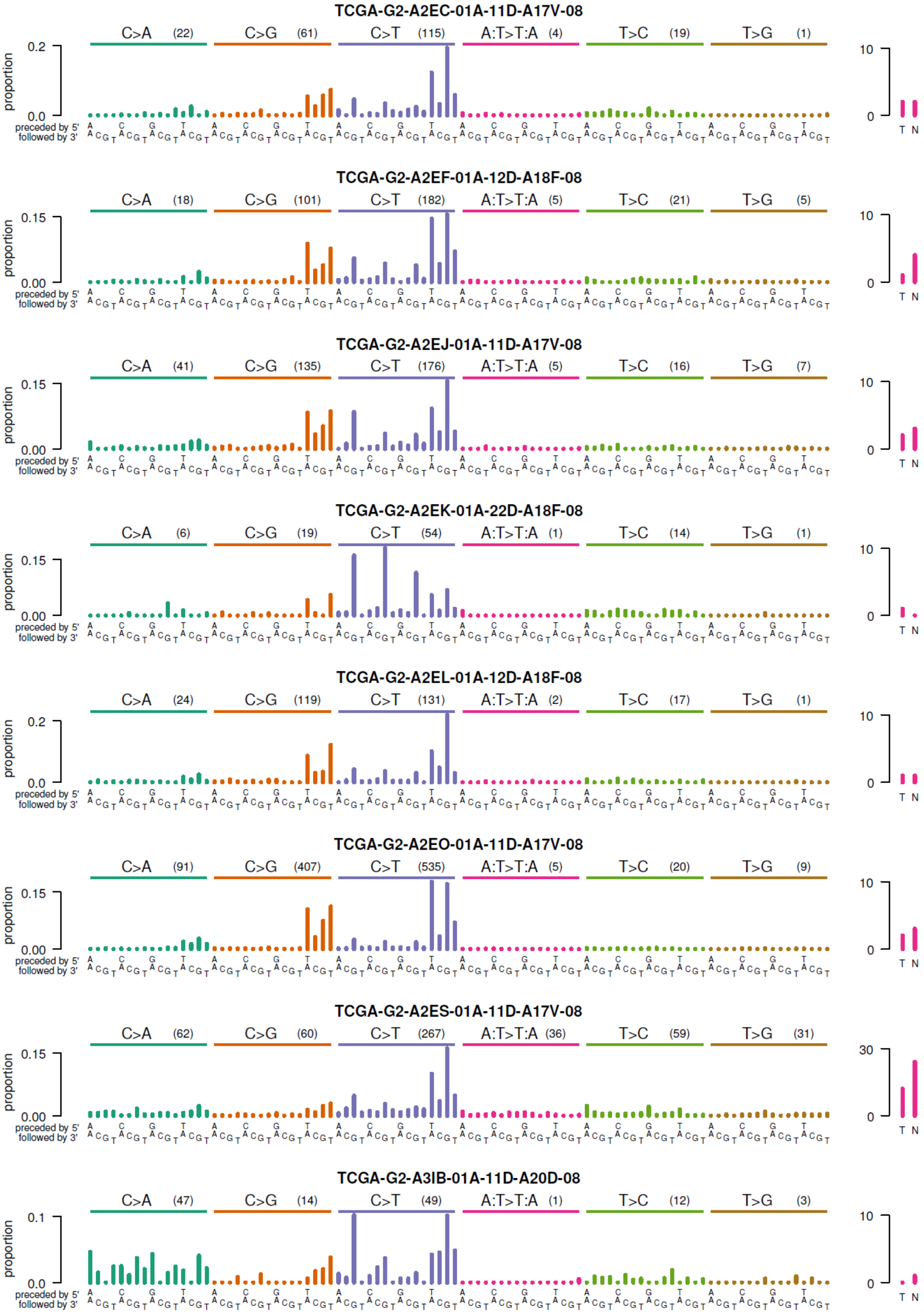

Supplementary Figure S4 continued. The mutation spectra of 237 bladder cancers with data from TCGA. The somatic mutation data from 237 TCGA (http://cancergenome.nih.gov/) urothelial bladder tumors were downloaded from the TCGA data portal (https://tcga-data.nci.nih.gov/tcga/) on 8 May 2014.

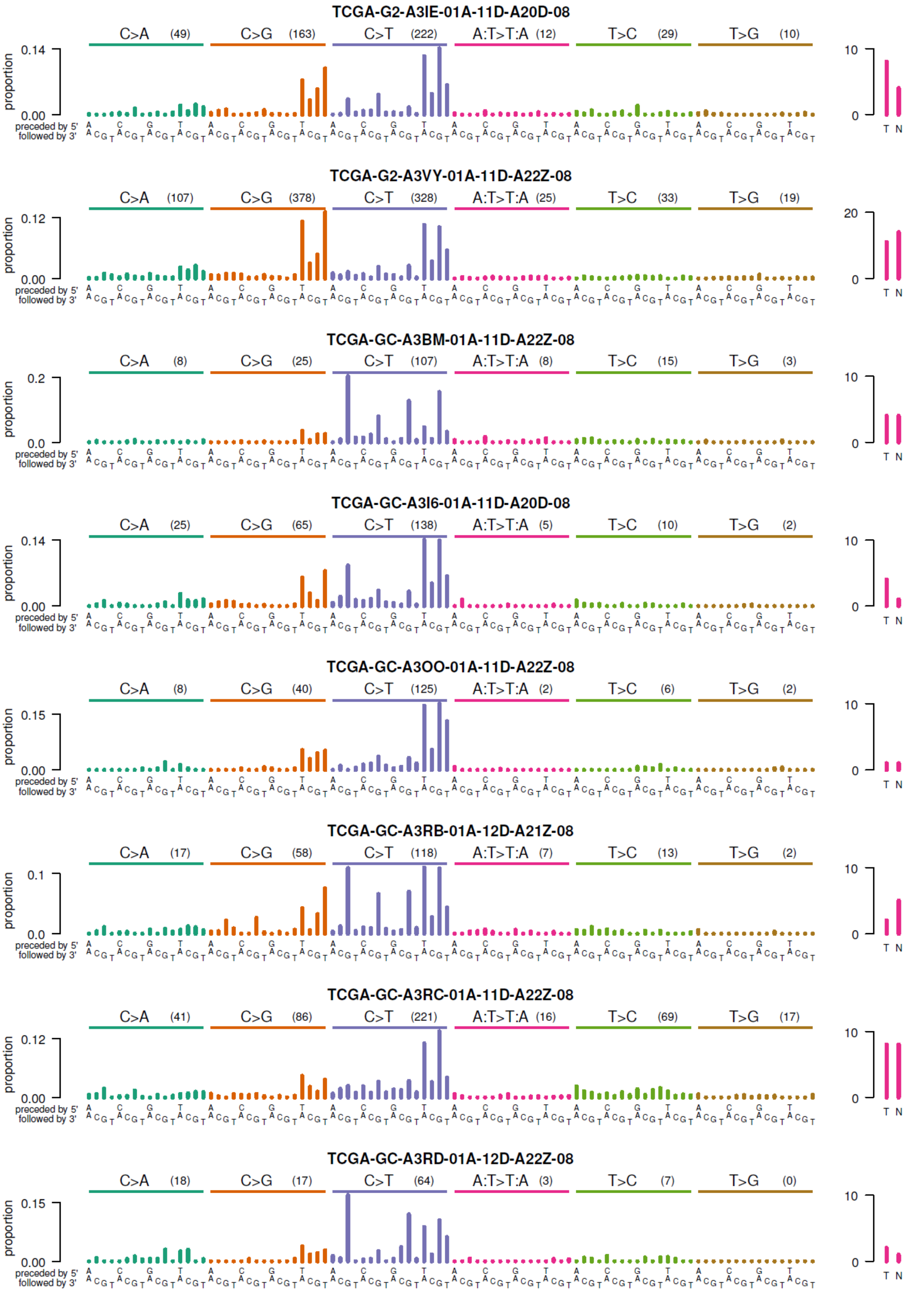

Supplementary Figure S4 continued. The mutation spectra of 237 bladder cancers with data from TCGA. The somatic mutation data from 237 TCGA (http://cancergenome.nih.gov/) urothelial bladder tumors were downloaded from the TCGA data portal (https://tcga-data.nci.nih.gov/tcga/) on 8 May 2014.

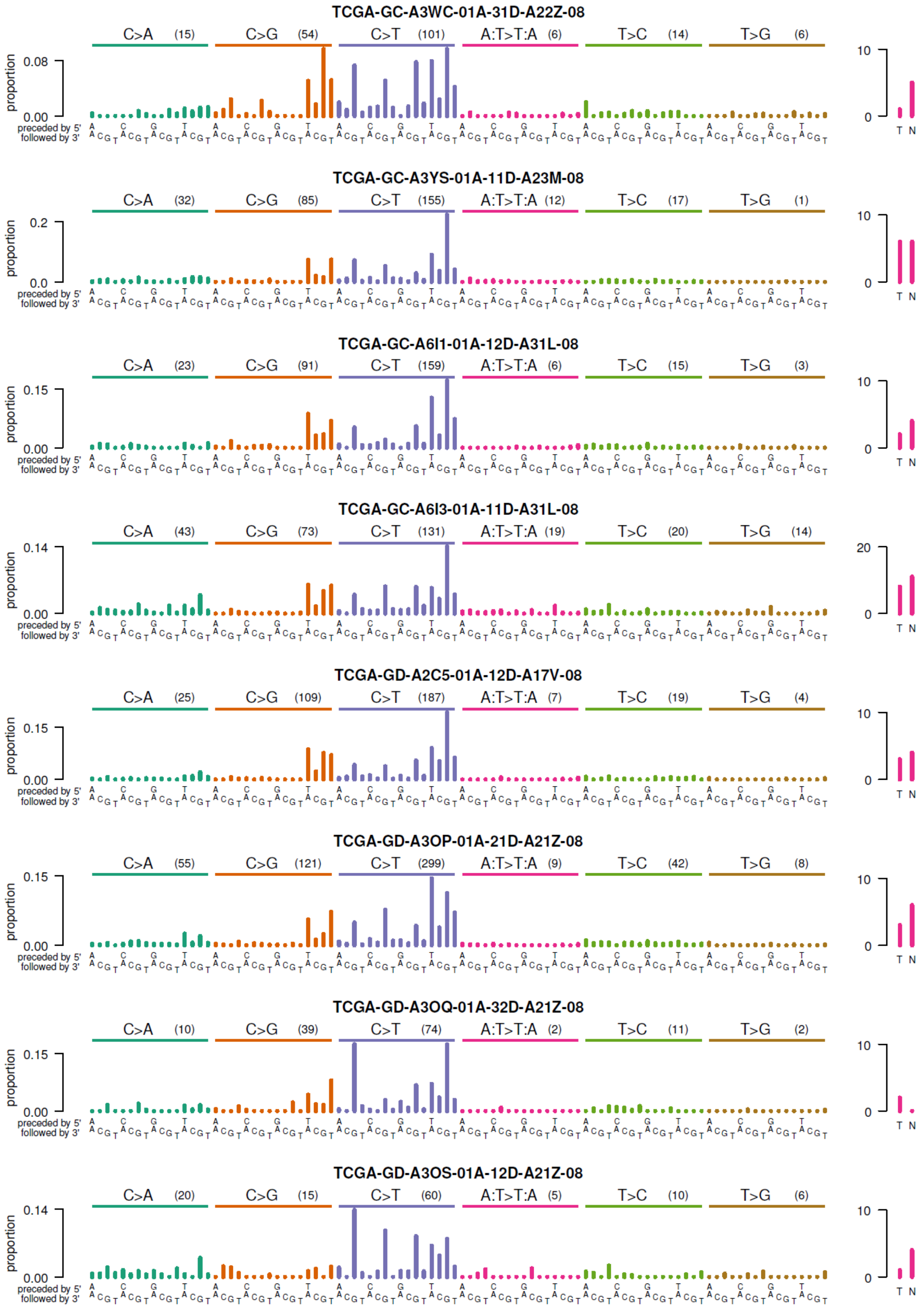

Supplementary Figure S4 continued. The mutation spectra of 237 bladder cancers with data from TCGA. The somatic mutation data from 237 TCGA (http://cancergenome.nih.gov/) urothelial bladder tumors were downloaded from the TCGA data portal (https://tcga-data.nci.nih.gov/tcga/) on 8 May 2014.

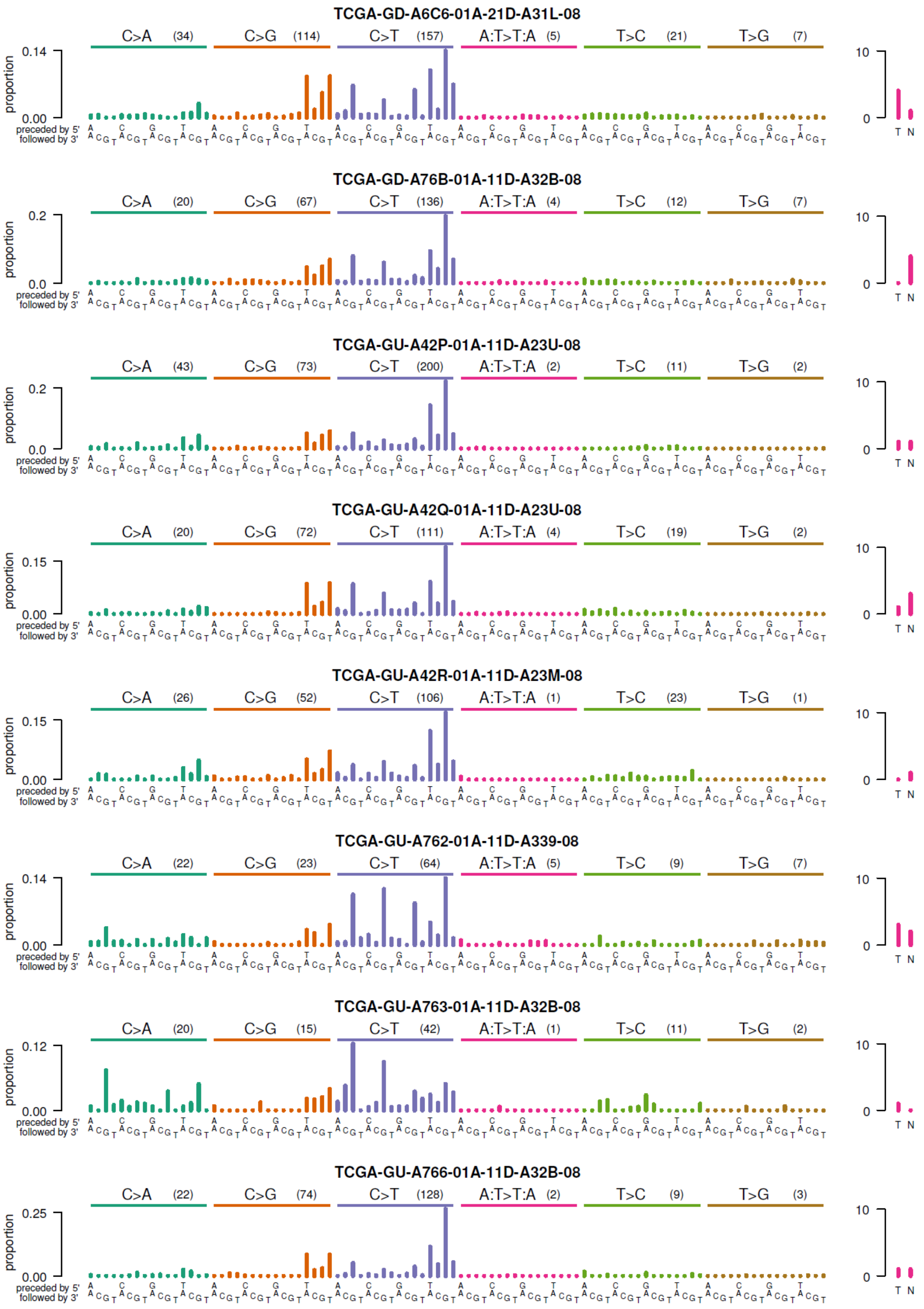

Supplementary Figure S4 continued. The mutation spectra of 237 bladder cancers with data from TCGA. The somatic mutation data from 237 TCGA (http://cancergenome.nih.gov/) urothelial bladder tumors were downloaded from the TCGA data portal (https://tcga-data.nci.nih.gov/tcga/) on 8 May 2014.

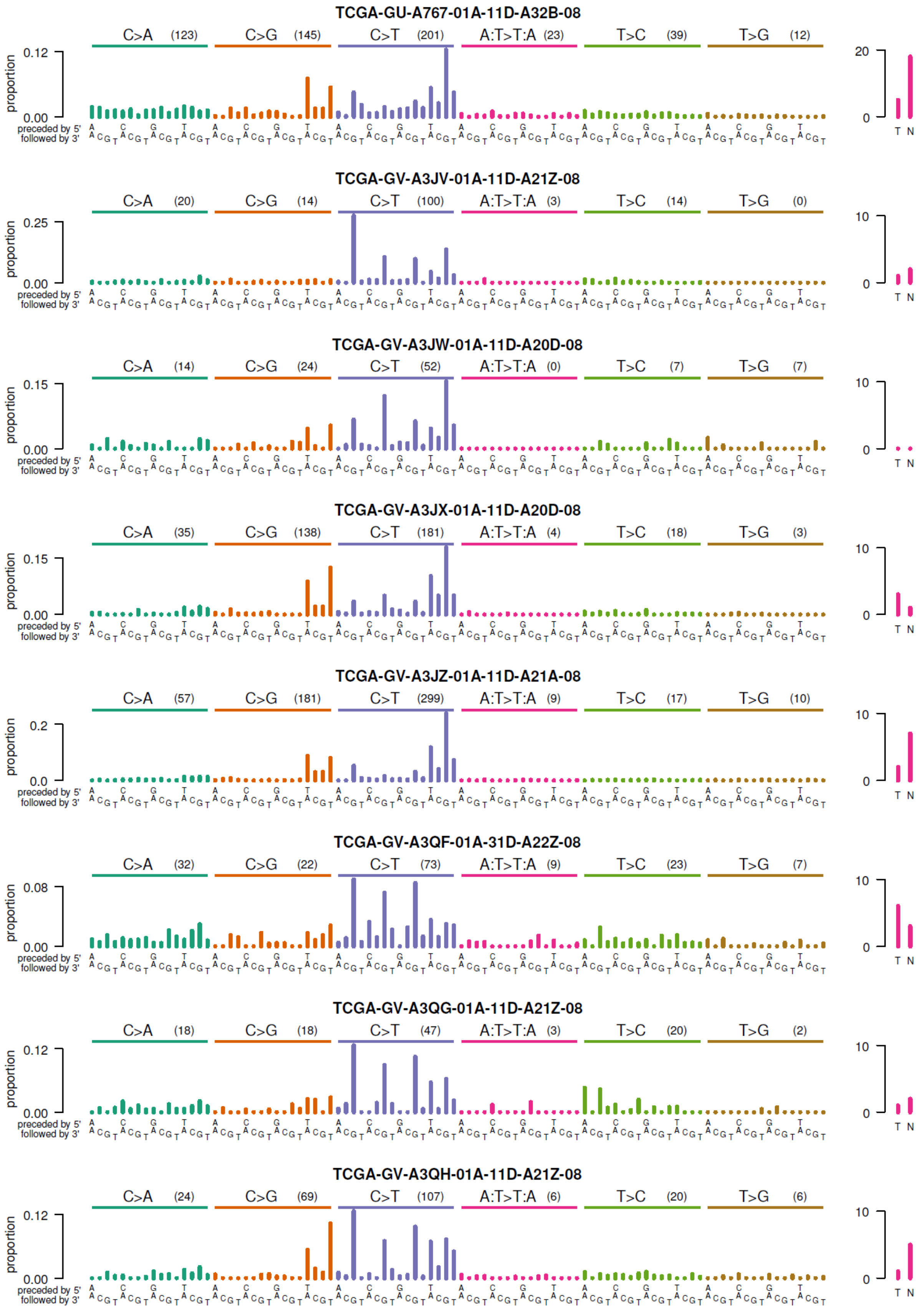

Supplementary Figure S4 continued. The mutation spectra of 237 bladder cancers with data from TCGA. The somatic mutation data from 237 TCGA (http://cancergenome.nih.gov/) urothelial bladder tumors were downloaded from the TCGA data portal (https://tcga-data.nci.nih.gov/tcga/) on 8 May 2014.

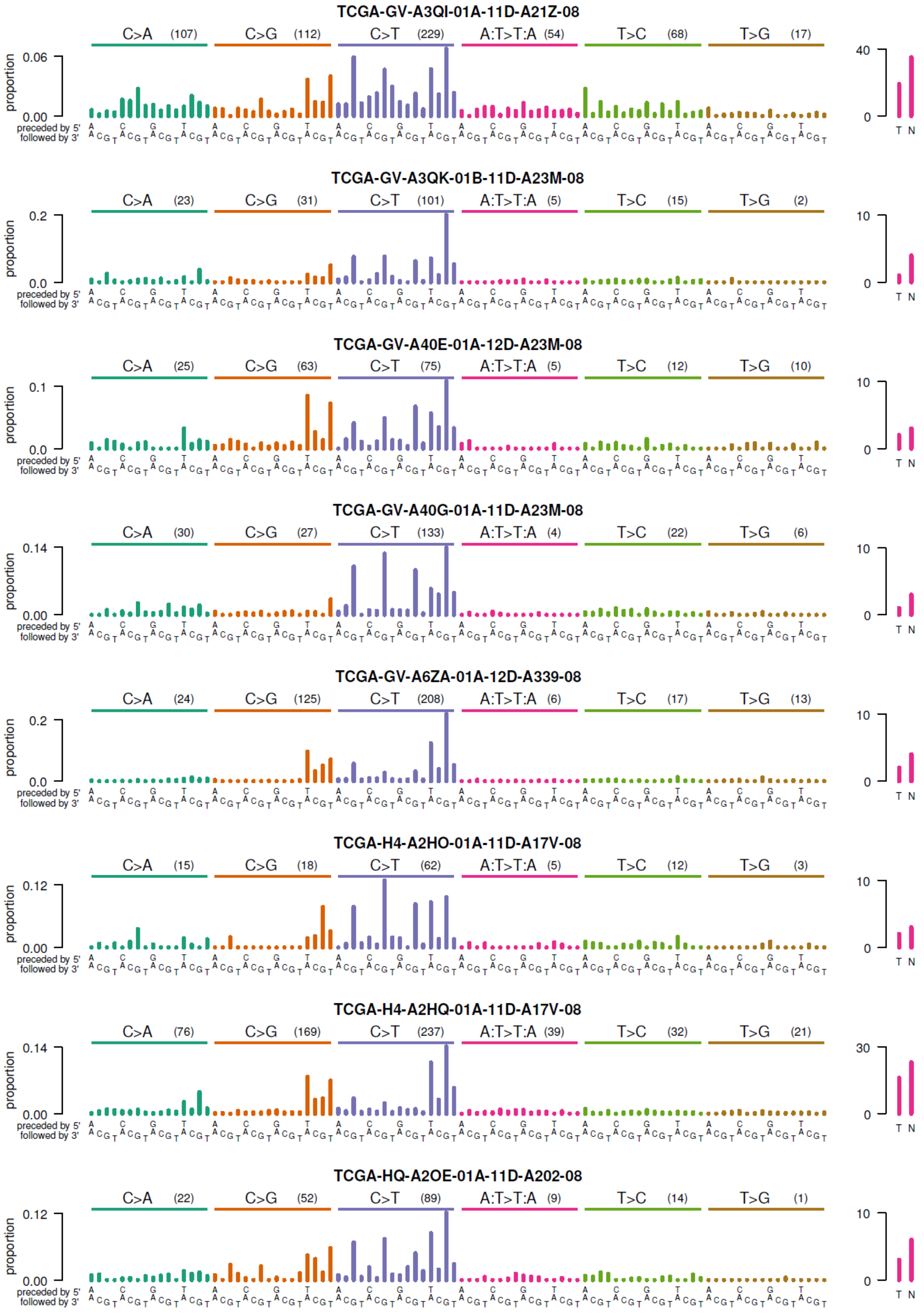

Supplementary Figure S4 continued. The mutation spectra of 237 bladder cancers with data from TCGA. The somatic mutation data from 237 TCGA (http://cancergenome.nih.gov/) urothelial bladder tumors were downloaded from the TCGA data portal (https://tcga-data.nci.nih.gov/tcga/) on 8 May 2014.

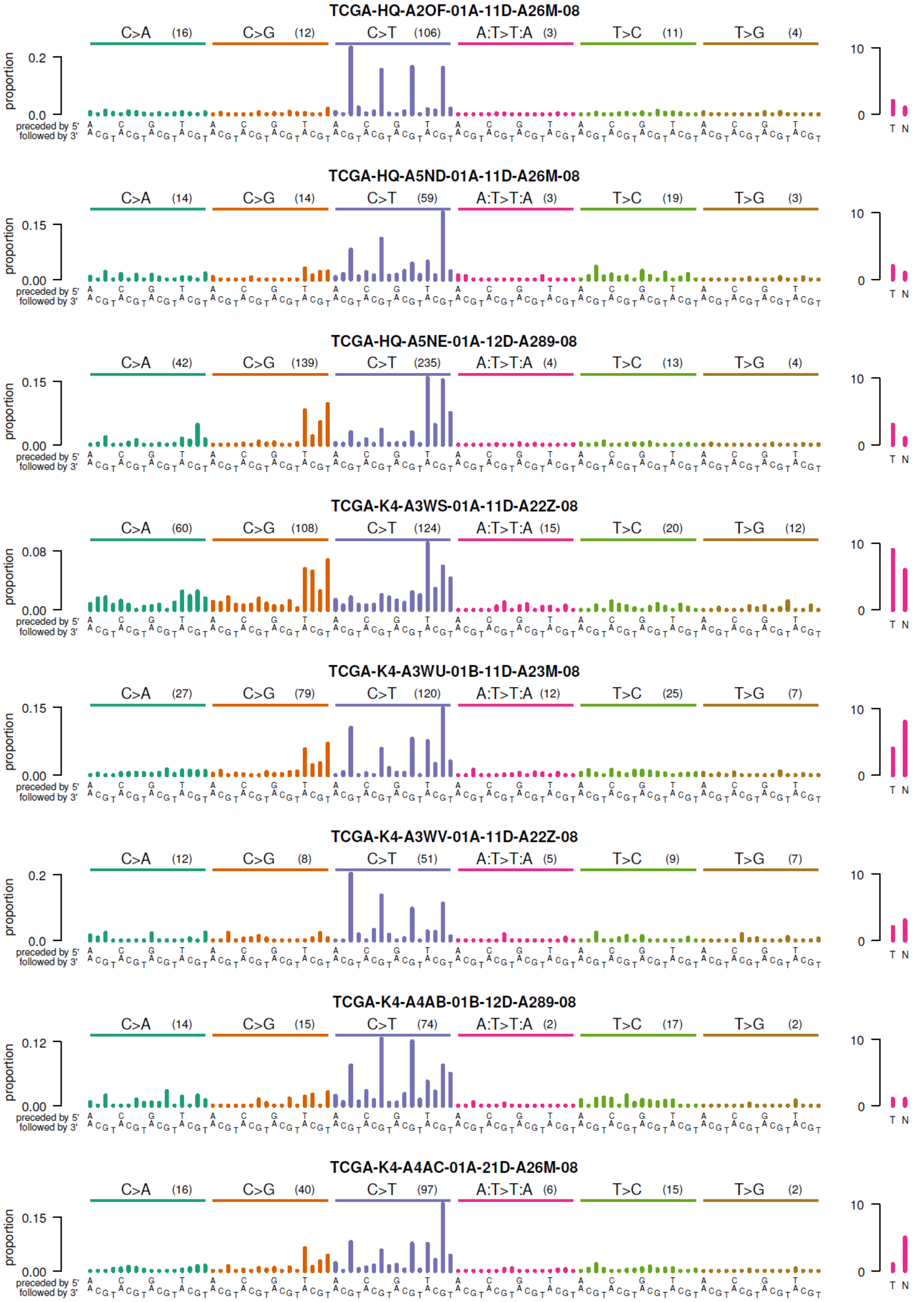

Supplementary Figure S4 continued. The mutation spectra of 237 bladder cancers with data from TCGA. The somatic mutation data from 237 TCGA (http://cancergenome.nih.gov/) urothelial bladder tumors were downloaded from the TCGA data portal (https://tcga-data.nci.nih.gov/tcga/) on 8 May 2014.

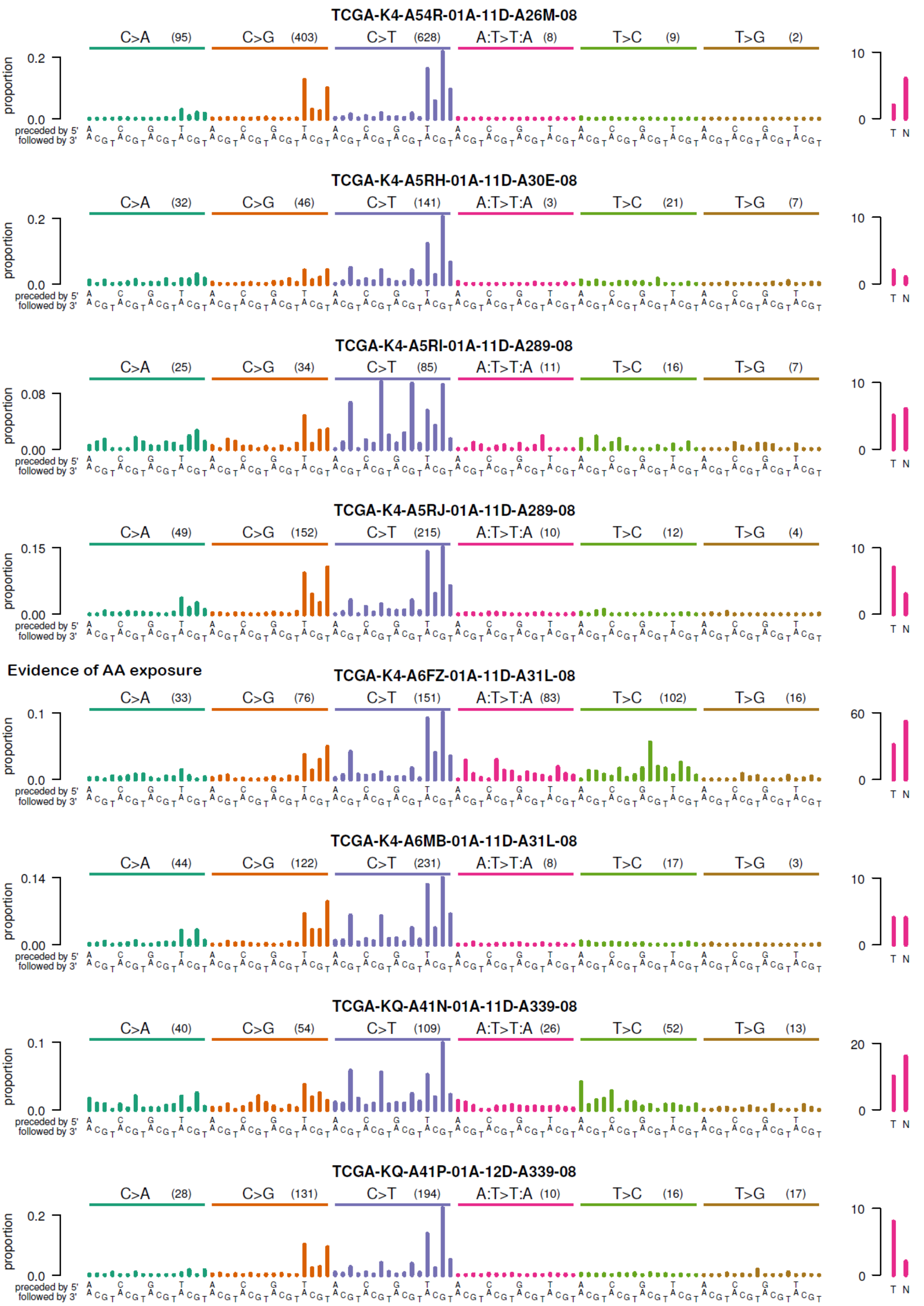

Supplementary Figure S4 continued. The mutation spectra of 237 bladder cancers with data from TCGA. The somatic mutation data from 237 TCGA (http://cancergenome.nih.gov/) urothelial bladder tumors were downloaded from the TCGA data portal (https://tcga-data.nci.nih.gov/tcga/) on 8 May 2014.

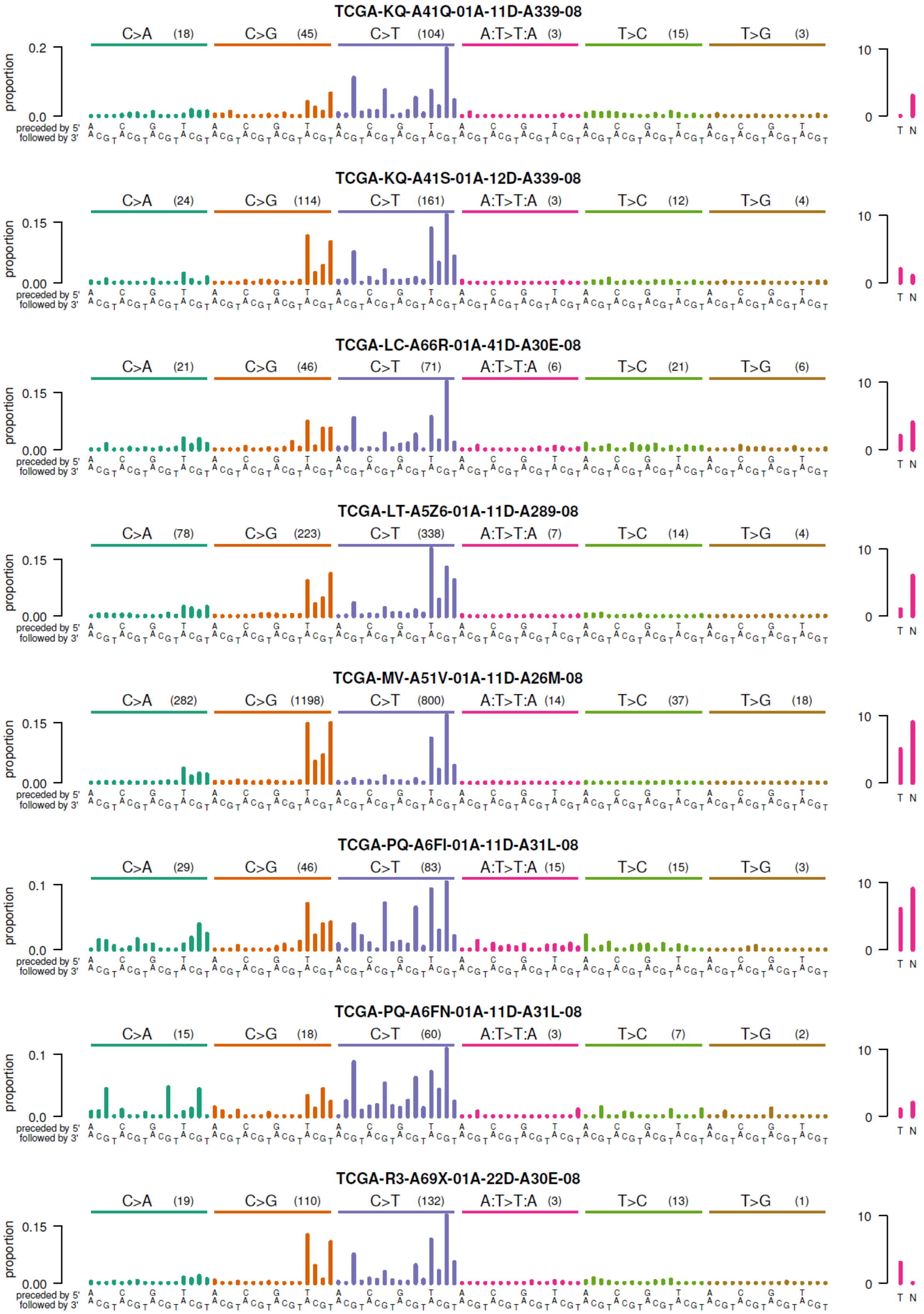

Supplementary Figure S4 continued. The mutation spectra of 237 bladder cancers with data from TCGA. The somatic mutation data from 237 TCGA (http://cancergenome.nih.gov/) urothelial bladder tumors were downloaded from the TCGA data portal (https://tcga-data.nci.nih.gov/tcga/) on 8 May 2014.

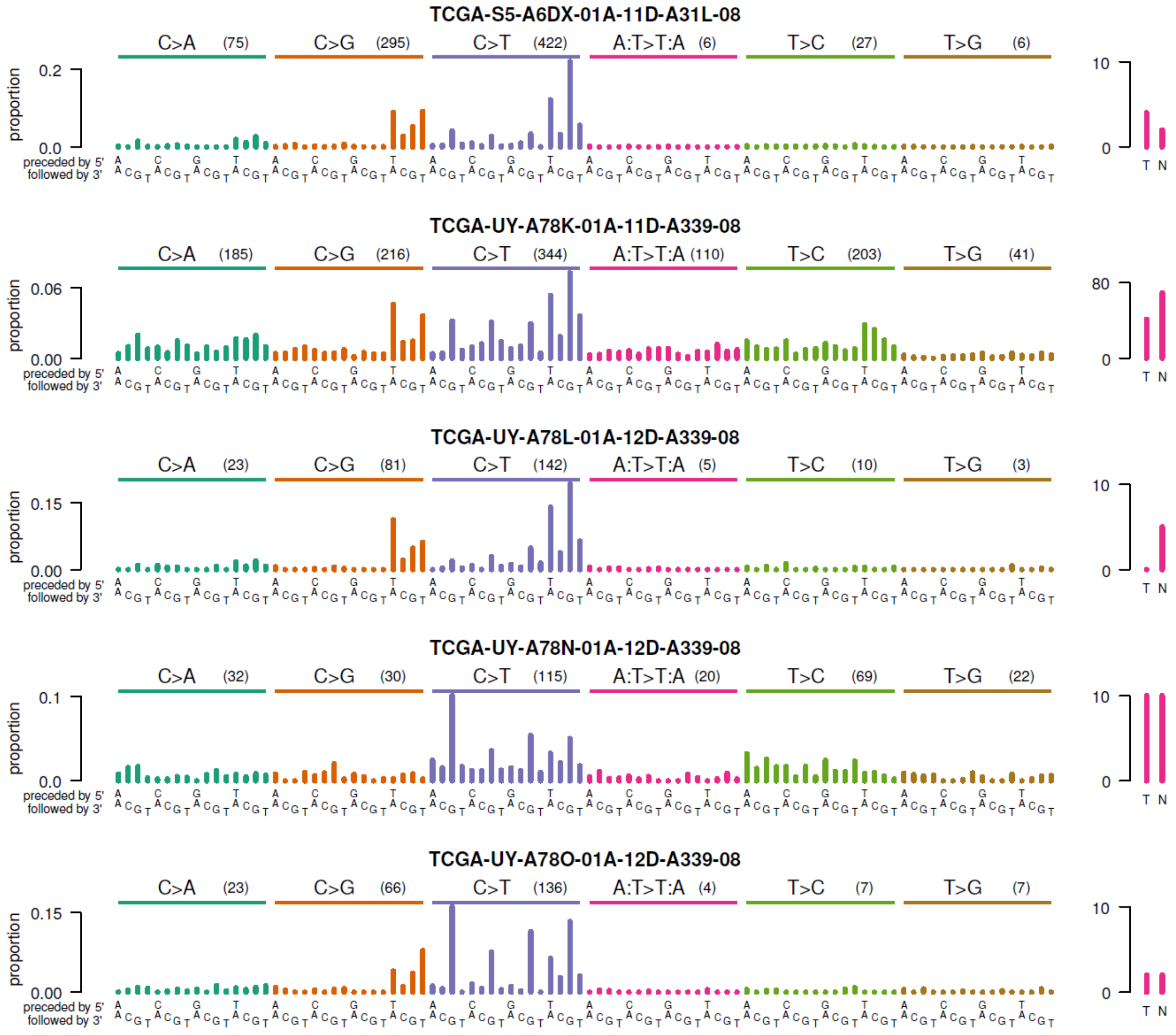

Supplementary Figure S4 continued. The mutation spectra of 237 bladder cancers with data from TCGA. The somatic mutation data from 237 TCGA (http://cancergenome.nih.gov/) urothelial bladder tumors were downloaded from the TCGA data portal (https://tcga-data.nci.nih.gov/tcga/) on 8 May 2014.

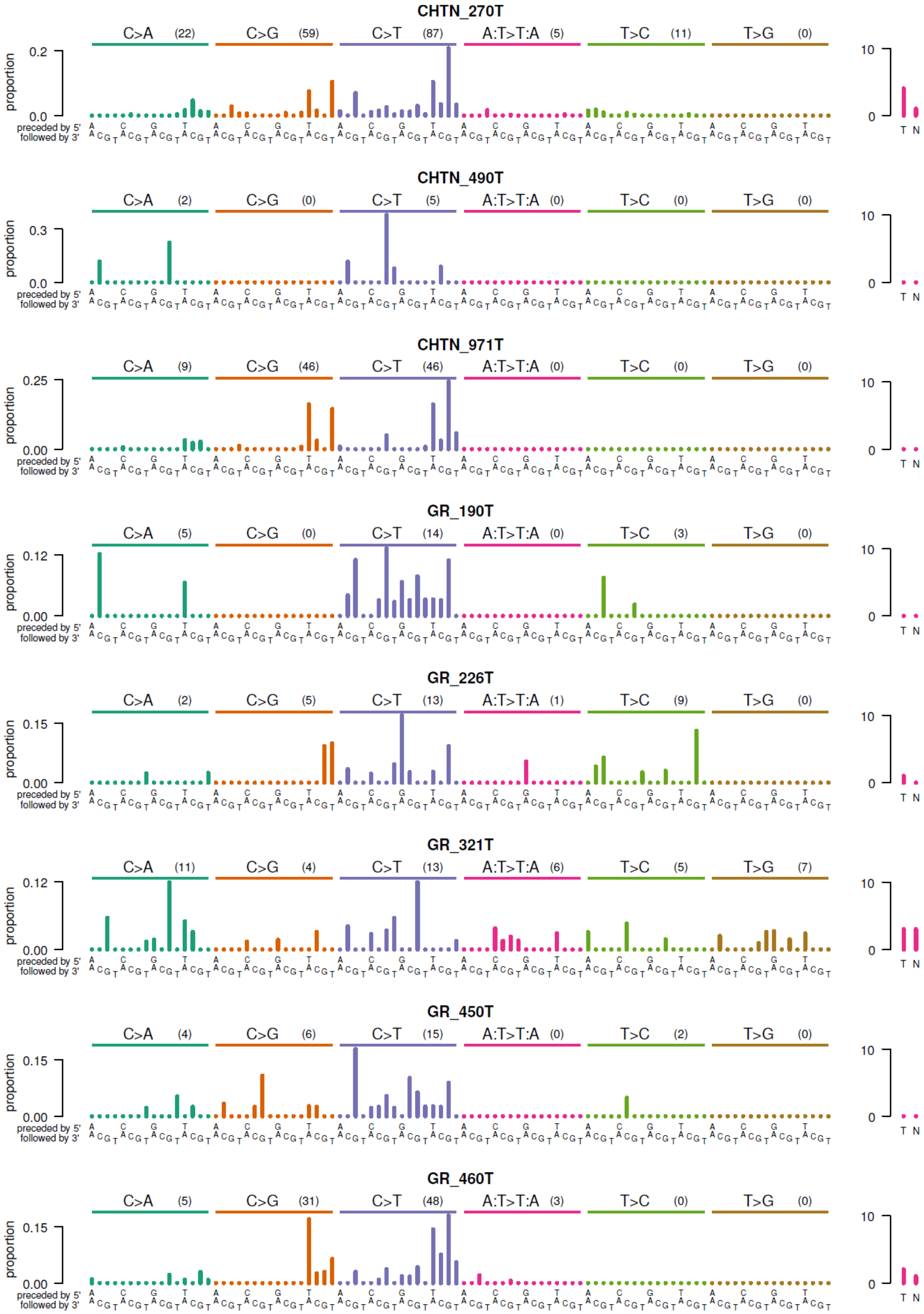

Supplementary Figure S5. The mutation spectra of 24 AA-associated and non-AA associated UTUCs [1].

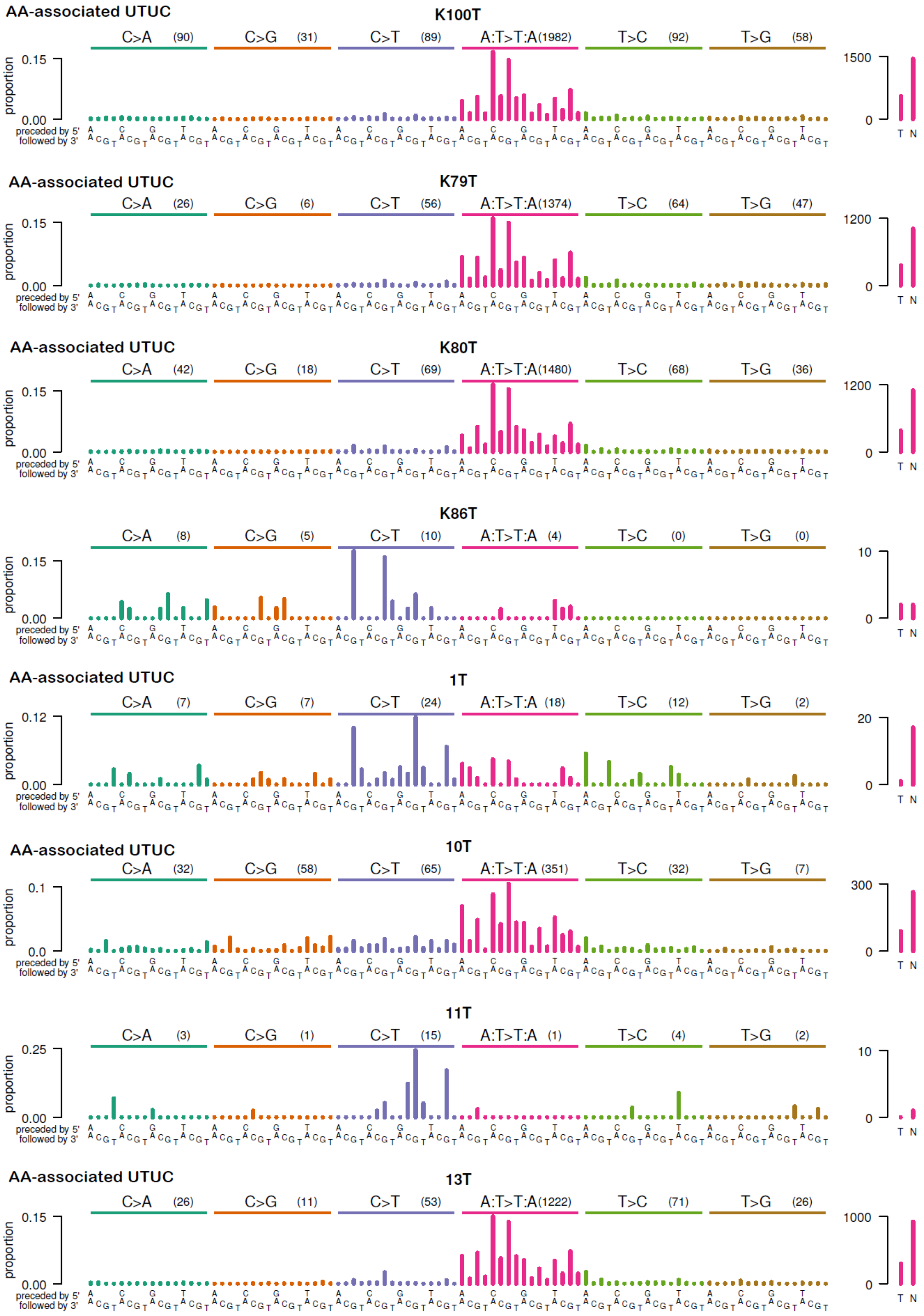

Supplementary Figure S5 continued. The mutation spectra of 24 AA-associated and non-AA associated UTUCs.

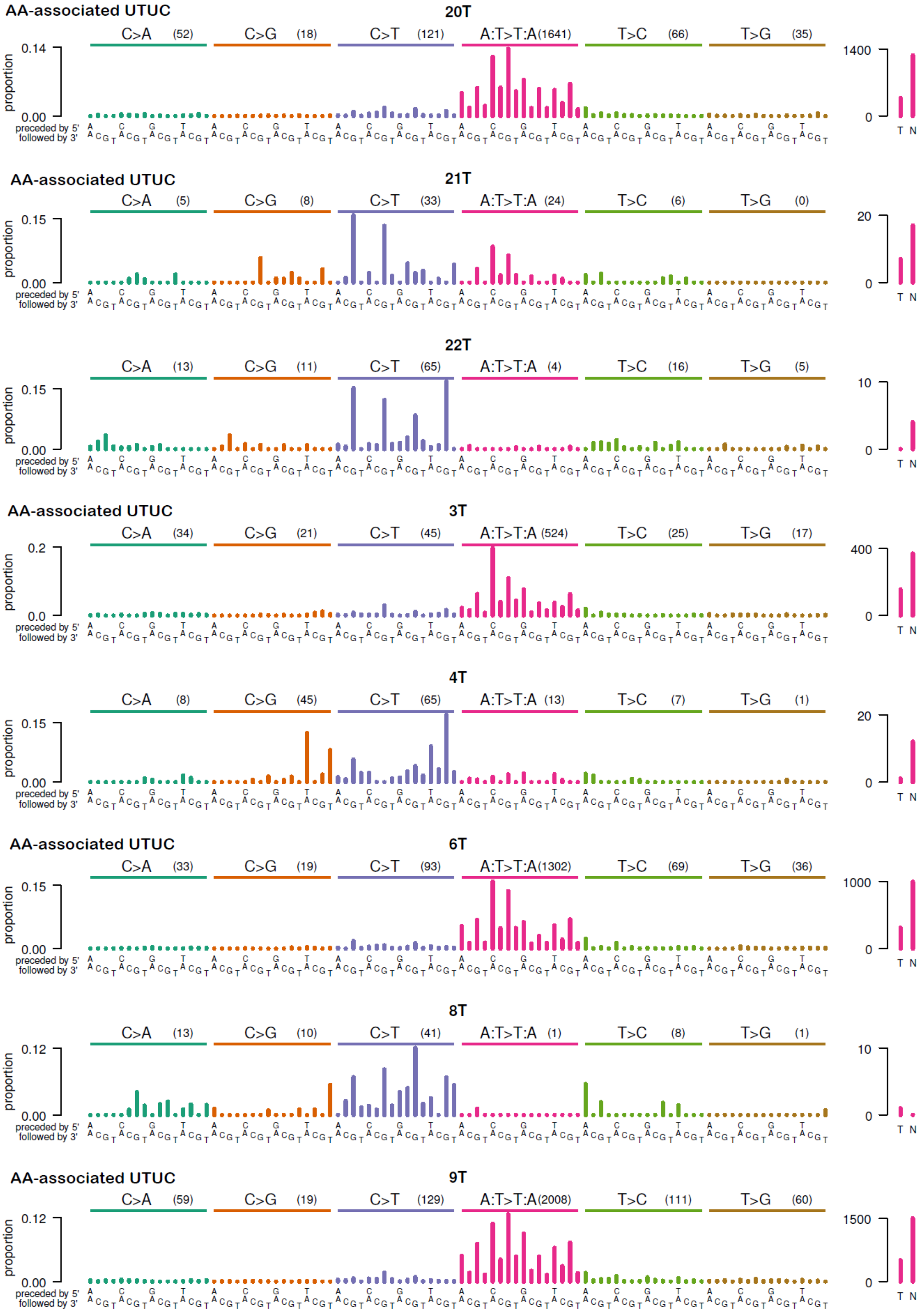

Supplementary Figure S5 continued. The mutation spectra of 24 AA-associated and non-AA associated UTUCs.

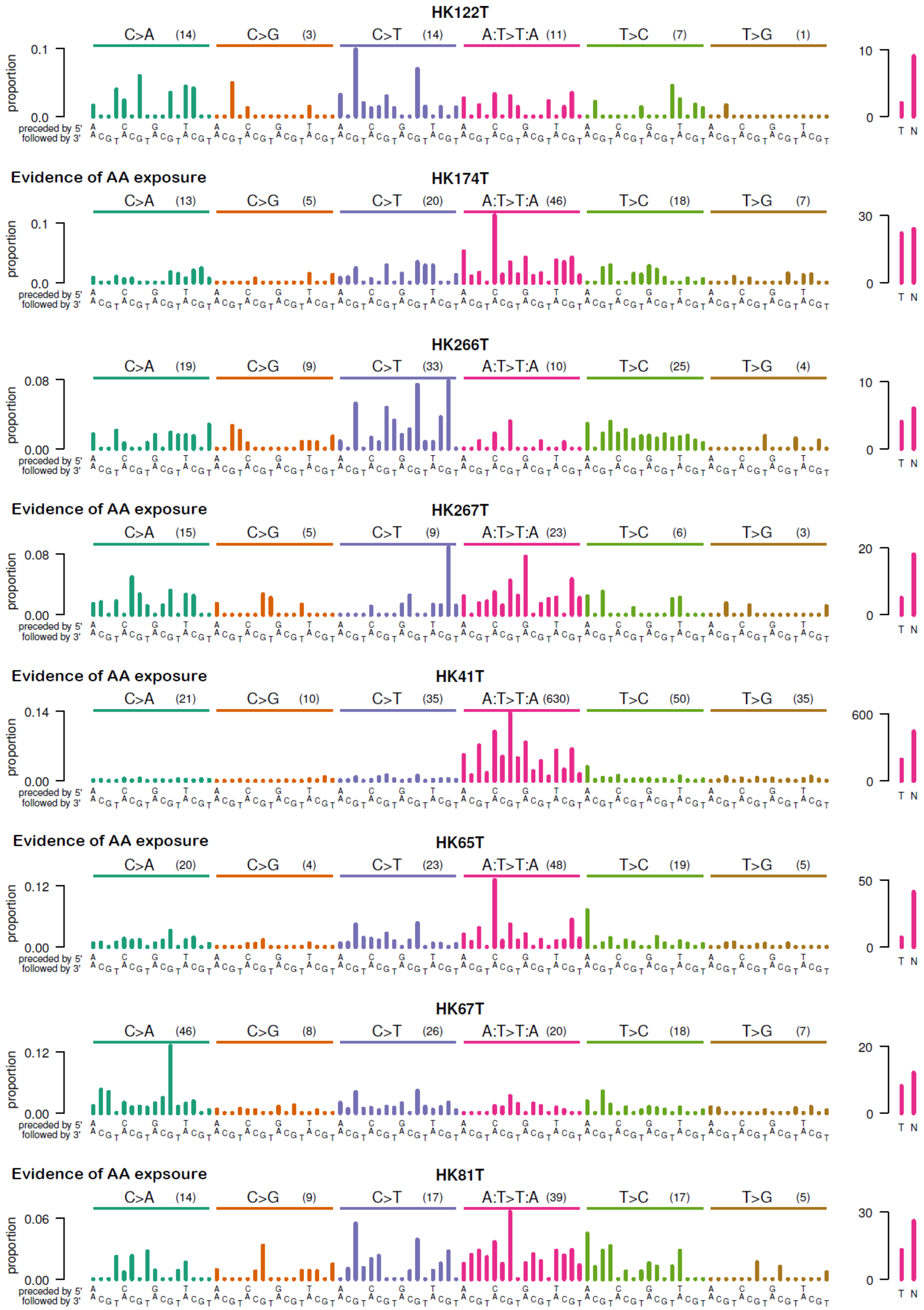

Supplementary Figure S6. The mutation spectra of 11 AA associated and non-AA associated HCCs [4].

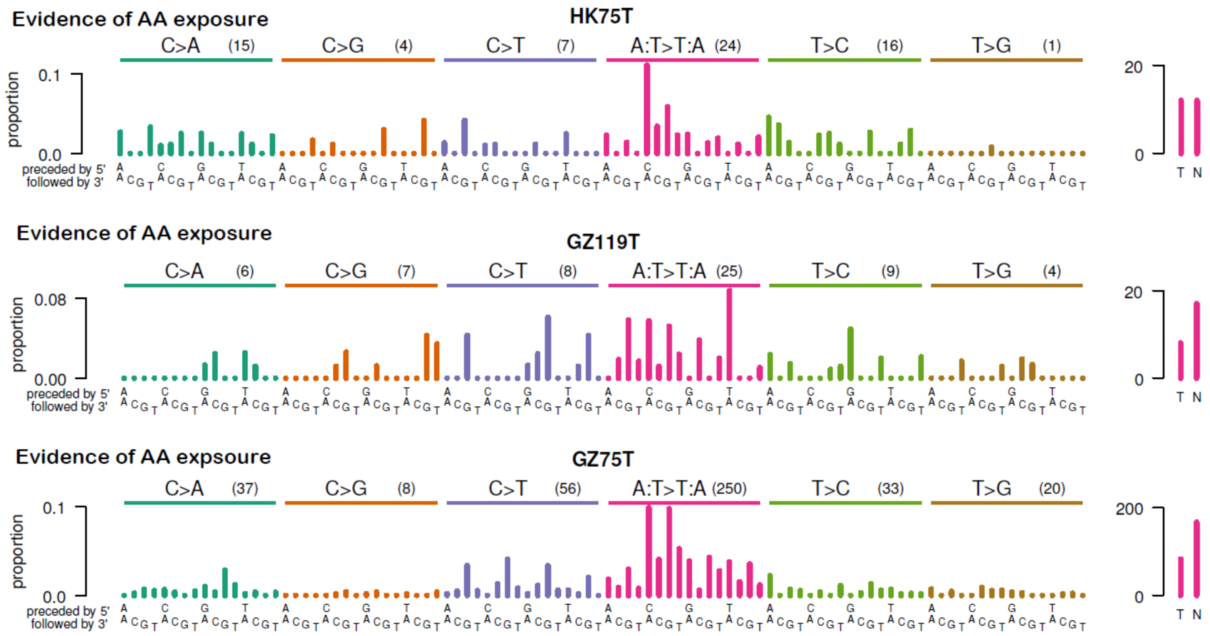

Supplementary Figure S6 continued. The mutation spectra of 11 AA associated and non-AA associated HCCs.

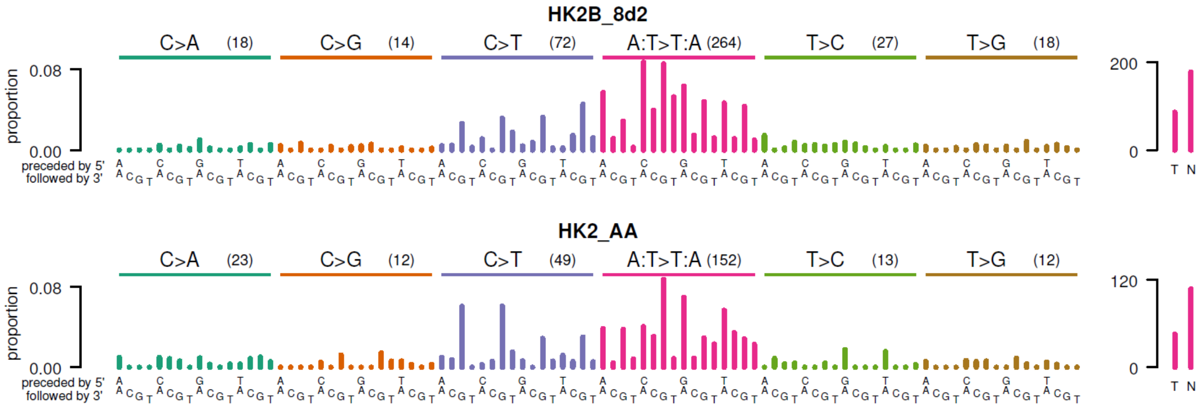

Supplementary Figure S7. The mutation spectra of two AA-treated cell lines [1].

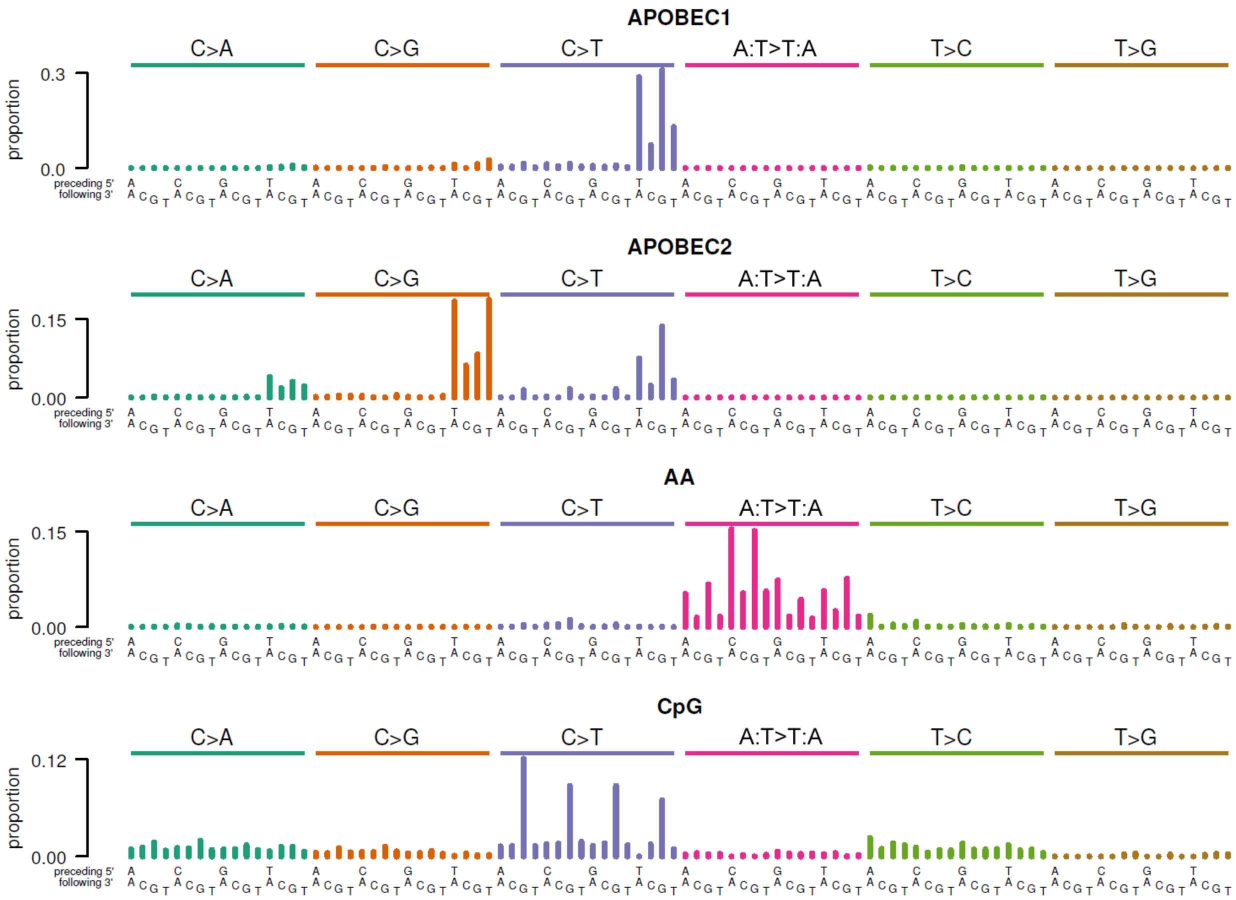

Supplementary Figure S8. Mutation signatures detected by NMF were substantially similar to those detected by EMu.

The AA and CpG signatures detected by NMF were similar to the corresponding signatures detected by EMu (compare Figure 3A). However, NMF split the APOBEC signature into two. Nevertheless, evidence from prior studies [5, 6] indicates that this signature likely represents a single mutational process. Both NMF and EMu ascribe to each tumor the number of somatic mutations due to each signature, and we examined the numbers of mutations in the tumors ascribed by the two methods. We found these to be extremely close for tumors for which the AA-ascribed mutation count was > 30 (Figure S9).

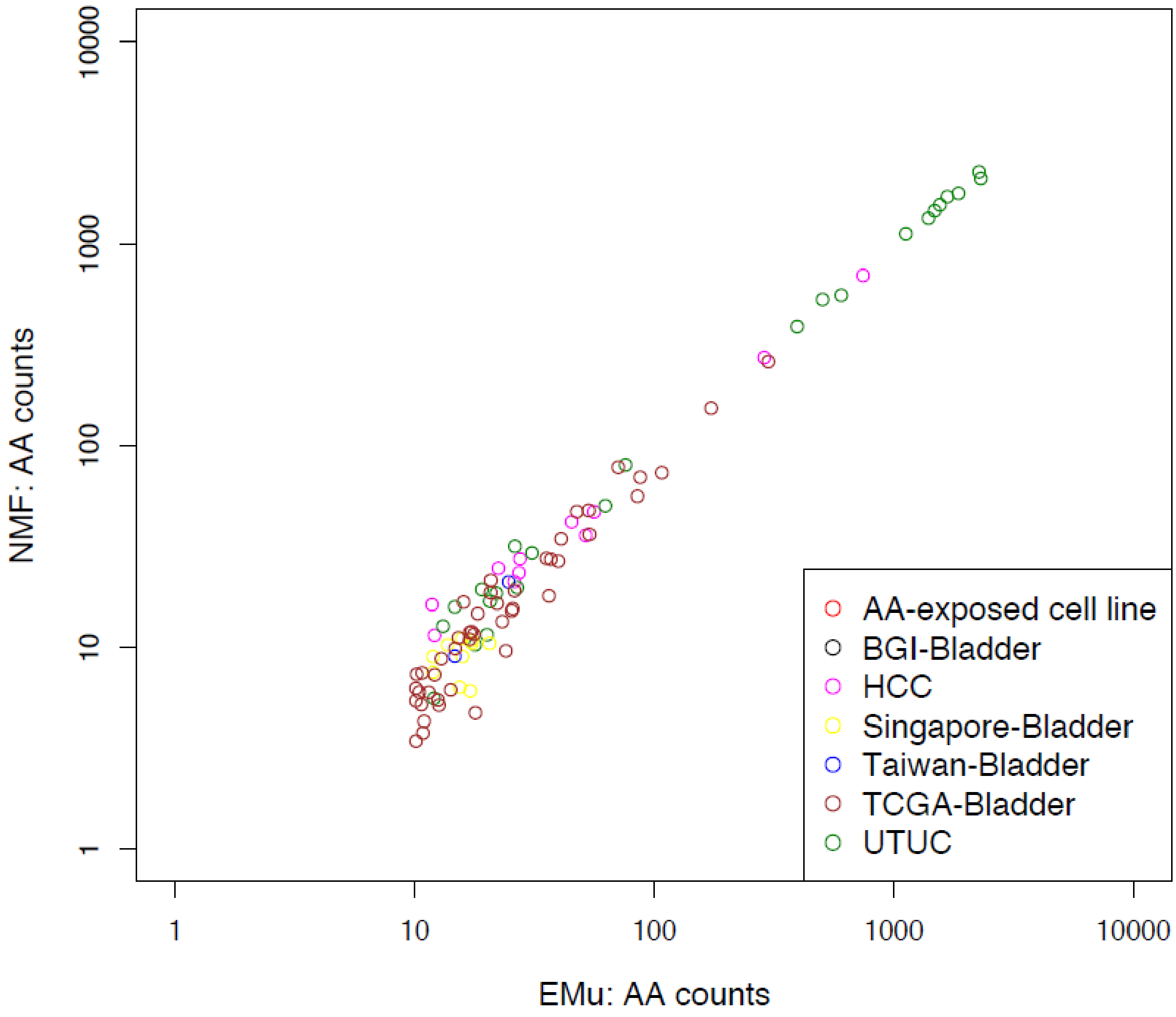

Supplementary Figure S9. The correlation of AA counts between EMu and NMF analyses.

EMu and NMF were run independently to analyze the mutation signature of 386 tumors, including 349 bladder cancers, 24 UTUCs, 2 AA-exposed cell lines, and 11 HCC. Tumors with > 10 mutations attributed to AA by EMu plotted against the AA mutation counts estimated by NMF.

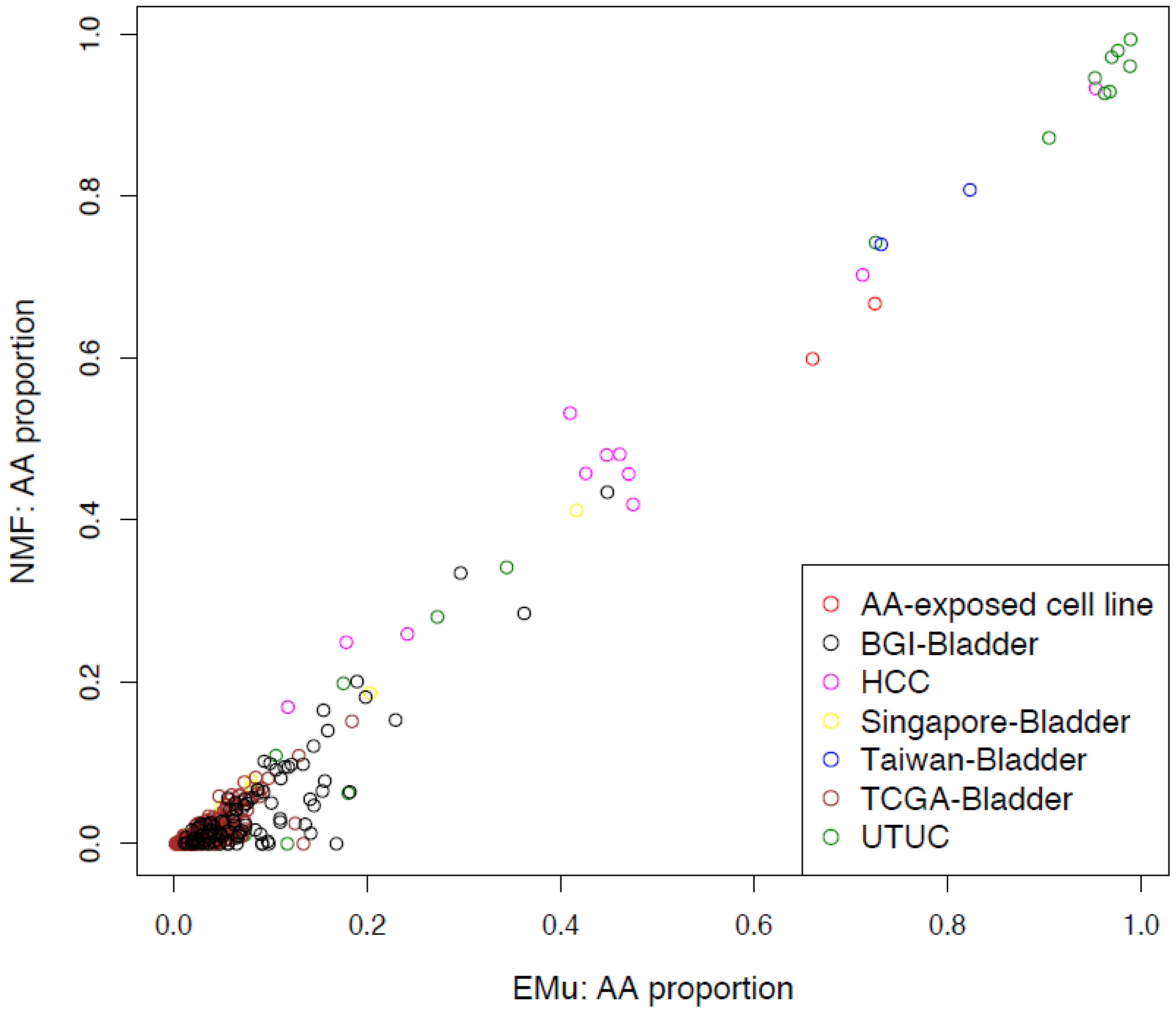

Supplementary Figure S10. The correlation of AA proportion between EMu and NMF analyses.

EMu and NMF were run independently to analyze the mutation signatures of 2 AA-exposed cell lines and 384 tumors, including 349 bladder cancers, 24 UTUCs, and 11 HCC. The proportions of mutations attributed to AA from each method were plotted against each other.

Supplementary Table S1. Clinical characteristics of 13 bladder cancer patients analyzed by whole-genome or whole exome sequencing.

Sample	Age at diagnosis	Characteristic	Grade	Herb intake	History of ESRD	History of UTUC	History of HCC
130T	79	Muscle invasive	High	Yes	No	No	No
136T	76	Muscle invasive	High	Yes	No	No	No
17475125T	80	Non-muscle invasive	Low	Unknown	No	No	No
31085175T	68	Muscle invasive	Low	Unknown	No	No	No
33324197T	80	Non-muscle invasive	Low	Unknown	No	No	No
42011796T	84	Non-muscle invasive	High	Unknown	No	No	No
43368963T	42	Non-muscle invasive	Low	Unknown	No	No	No
48647323T	69	Non-muscle invasive	High	Unknown	No	No	No
61487606T	73	Muscle invasive	High	Unknown	No	No	No
85262131T	76	Non-muscle invasive	High	Unknown	No	No	No
91168215T	76	Non-muscle invasive	High	Unknown	No	No	No
92130677T	83	Muscle invasive	High	Unknown	No	No	No
Z1229T	74	Non-muscle invasive	High	Unknown	No	No	No

UTUC = upper urinary tract urothelial cell carcinoma

HCC = hepatocellular carcinoma

ESRD = end stage renal disease

Supplementary Table S2. Sequence analysis summary of 13 bladder tumors and matched normal tissue.

Sample	Normal or tumor	Genome or exome	Number of somatic mutations identified in targeted region	Average depth per targeted base	% of targeted bases with depth ≥ 1	% of targeted bases with depth ≥ 20
130T	Normal	Genome		15	95.1	29
	Tumor	Genome	1366	22	96	53
136T	Normal	Genome		16	95.1	33
	Tumor	Genome	688	35	96.1	86
17475125T	Normal	Exome		83	95.7	85
	Tumor	Exome	91	96	95.7	86
31085175T	Normal	Exome		121	95.8	88
	Tumor	Exome	404	80	95.5	84
33324197T	Normal	Exome		88	95.6	85
	Tumor	Exome	182	86	95.5	85
42011796T	Normal	Exome		65	95.3	82
	Tumor	Exome	152	69	95.3	82
43368963T	Normal	Exome		91	95.6	86
	Tumor	Exome	273	85	95.6	85
48647323T	Normal	Exome		75	95.4	83
	Tumor	Exome	61	173	96	90
61487606T	Normal	Exome		81	95.7	85
	Tumor	Exome	765	85	95.7	85
85262131T	Normal	Exome		87	95.7	85
	Tumor	Exome	442	81	95.6	84
91168215T	Normal	Exome		90	95.7	85
	Tumor	Exome	411	96	95.8	86
92130677T	Normal	Exome		80	95.5	84
	Tumor	Exome	160	77	95.5	84
Z1229T	Normal	Exome		75	95.6	84
	Tumor	Exome	1127	84	95.6	85

Supplementary Table S3. Frequencies of trinucleotides centered at A in the human exome.

Trinucleotide	Counts of occurrence in SureSelect Version 2		Frequency among trinucleotides centered at "A"
	By strand	Both strands	
5'-AAA-3'	504574	1009430	0.068011618
3'-TTT-5'	504856		
AAC	362548	844502	0.056899386
TTG	481954		
AAG	595845	1180383	0.079529792
TTC	584538		
AAT	409419	720261	0.048528492
TTA	310842		
CAA	480207	843431	0.056827226
GTT	363224		
CAC	538549	1074518	0.072397004
GTG	535969		
CAG	857300	1246212	0.083965104
GTC	388912		
CAT	507156	803862	0.054161215
GTA	296706		
GAA	580247	1181246	0.079587938
CTT	600999		
GAC	387501	1245495	0.083916795
CTG	857994		
GAG	626721	1258310	0.084780222
CTC	631589		
GAT	399895	653191	0.044009567
CTA	253296		
TAA	310437	720305	0.048531457
ATT	409868		
TAC	298659	804205	0.054184325
ATG	505546		
TAG	252941	652624	0.043971364
ATC	399683		
TAT	301181	604048	0.040698495
ATA	302867		

Supplementary References

1. Poon SL, Pang ST, McPherson JR, Yu W, Huang KK, Guan P, Weng WH, Siew EY, Liu Y, Heng HL, et al: **Genome-wide mutational signatures of aristolochic acid and its application as a screening tool.** *Sci Transl Med* 2013, **5:**197ra101.
2. Guo G, Sun X, Chen C, Wu S, Huang P, Li Z, Dean M, Huang Y, Jia W, Zhou Q, et al: **Whole-genome and whole-exome sequencing of bladder cancer identifies frequent alterations in genes involved in sister chromatid cohesion and segregation.** *Nat Genet* 2013, **45:**1459-1463.
3. Cancer Genome Atlas Research Network: **Comprehensive molecular characterization of urothelial bladder carcinoma.** *Nature* 2014, **507:**315-322.
4. Sung WK, Zheng H, Li S, Chen R, Liu X, Li Y, Lee NP, Lee WH, Ariyaratne PN, Tennakoon C, et al: **Genome-wide survey of recurrent HBV integration in hepatocellular carcinoma.** *Nat Genet* 2012, **44:**765-769.
5. Alexandrov LB, Nik-Zainal S, Wedge DC, Aparicio SA, Behjati S, Biankin AV, Bignell GR, Bolli N, Borg A, Borresen-Dale AL, et al: **Signatures of mutational processes in human cancer.** *Nature* 2013, **500:**415-421.
6. Fischer A, Illingworth CJ, Campbell PJ, Mustonen V: **EMu: probabilistic inference of mutational processes and their localization in the cancer genome.** *Genome Biol* 2013, **14:**R39.
